# Multistep Synthesis and In Vitro Anticancer Evaluation of 2-Pyrazolyl-Estradiol Derivatives, Pyrazolocoumarin-Estradiol Hybrids and Analogous Compounds

**DOI:** 10.3390/molecules25184039

**Published:** 2020-09-04

**Authors:** Barnabás Molnár, Mohana Krishna Gopisetty, Dóra Izabella Adamecz, Mónika Kiricsi, Éva Frank

**Affiliations:** 1 Department of Organic Chemistry, Doctoral School of Chemistry, University of Szeged, Dóm tér 8, H-6720 Szeged, Hungary; barnabas.molnar@chem.u-szeged.hu; 2 Department of Biochemistry and Molecular Biology, Doctoral School of Biology, University of Szeged, Közép fasor 52, H-6726 Szeged, Hungary; gmohanakrishna@bio.u-szeged.hu (M.K.G.); doraadamecz@gmail.com (D.I.A.); kiricsim@bio.u-szeged.hu (M.K.)

**Keywords:** steroids, heterocyclic estradiol derivatives, Friedel-Crafts acetylation, arylhydrazones, Vilsmeier-Haack reaction, 4-formyl-pyrazoles, pyrazolocoumarins, anticancer activity

## Abstract

Although the hormone independent cytotoxic activity of several estradiol derivatives endowed with a simple substituent at C-2 has been reported so far, 2-heterocyclic and 2,3-condensed analogs are less investigated from both synthetic and pharmacological points of view. Therefore, novel A-ring-connected 2-pyrazoles of estradiol and, for comparison, their structurally simplified non-steroidal pairs were synthesized from estradiol 3-methyl ether and 6-methoxy-1,2,3,4-tetrahydronaphthalene. Friedel-Crafts acetylation of the protected phenolic compounds and subsequent *O*-demethylation led to *ortho*-substituted derivatives regioselectively, which were converted to arylhydrazones with phenylhydrazine, 4-tolylhydrazine and 4-chloro-phenylhydrazine, respectively, under microwave conditions. The hydrazones were subjected to cyclization with the Vilsmeier-Haack reagent immediately after preparation and the ring closure/formylation sequence resulted in steroidal and non-steroidal 4′-formylpyrazoles in moderate to good yields. During reductive transformations, 4-hydroxymethyl-pyrazoles were obtained, while oxidative lactonization of the 4-formylpyrazole moiety with the phenolic OH in the presence of the Jones reagent afforded A-ring-integrated pyrazolocoumarin hybrids and related analogs. Steroidal pyrazoles, which were produced as C-17 acetates due to acetylation of C-17 OH during the primary Friedel-Crafts reaction, underwent deacetylation in alkaline methanol to furnish 2-heterocyclic estradiol derivatives. Pharmacological studies revealed the overall and cancer cell-specific cytotoxicity of the derivatives and the half maximal inhibitory concentrations were obtained for the most promising compounds.

## 1. Introduction

Natural steroids play an important role in several fields of medicine, including contraception, treatment of inflammation, asthma, cardiovascular disease, osteoporosis, cancer, and other disorders [1,2]. During chemical modifications, steroids are often combined with other drugs or relevant pharmacophores via covalent bonds either acting as an effective agent or inactive carrier, or through domain integration of key structural units [3,4]. These multifunctional hybrid drugs or “chimeras”, which primarily serve to combat drug resistance, reduce unwanted side effects and enrich the arsenal of existing medications, offer novel therapeutic options for many diseases, including cancer, and are usually more active and specific than conventional treatments [5]. Nevertheless, steroids are privileged structures for molecular hybridization approaches due to their optically pure chiral character, conformational diversity with a varying possibility of functionalization, a hydrophobic nature that facilitates cell membrane penetration, and a wide spectrum of bioactivity [6].

In addition to their normal physiological functions, endogenous sex hormones are also involved in the development and progression of breast, ovarian, and prostate cancers [7]. The incorporation or connection of different heterocyclic scaffolds to the relatively rigid sterane framework of natural sex hormones can furnish a dramatic change in the biological properties of the parent compound [7,8,9]. Modulation of the binding ability provided by the heteroring via hydrogen bonding or coordinative interactions is considered a pivotal factor in the development of altered pharmacological effects [10]. Furthermore, certain pharmacokinetic properties, like water solubility, intestinal absorption, and stability against metabolic degradation can also be improved. Some heteroaromatic rings are frequently used as bioisosteres of hydrolysis-sensitive functional groups due to structural mimicry. In contrast to the effect of natural sex hormones on cell proliferation in hormone-dependent tumors, the anticancer properties of several sterane-based molecular hybrids [11,12], most of which have been obtained by modifying the D-ring of the steroid backbone with a heterocycle [7], have been previously reported. Amongst them, the most well investigated heterocyclic steroids, suitable for the treatment of advanced prostate cancer, are the androstane-based inhibitors of CYP17, a key regulatory enzyme involved in the androgen biosynthesis [2,13]. Some D-ring-modified estrone derivatives were also studied in hormone-dependent breast cancers as inhibitors of the aromatase enzyme responsible for estrogen production [14]. Besides enzyme inhibitors, a number of androstane- and estrane-based hybrids containing different heterocyclic motifs connected to, condensed with, or structurally integrated in the D-ring of the sterane framework were reported to exert direct antiproliferative action in tumor cells by affecting the apoptotic machinery [15,16,17]. Nevertheless, whether it is an enzyme inhibitor or a direct cytotoxic agent, the primary hormonal effect of the parent compound is unfavorable and should be reduced or eliminated.

**Figure 1:**
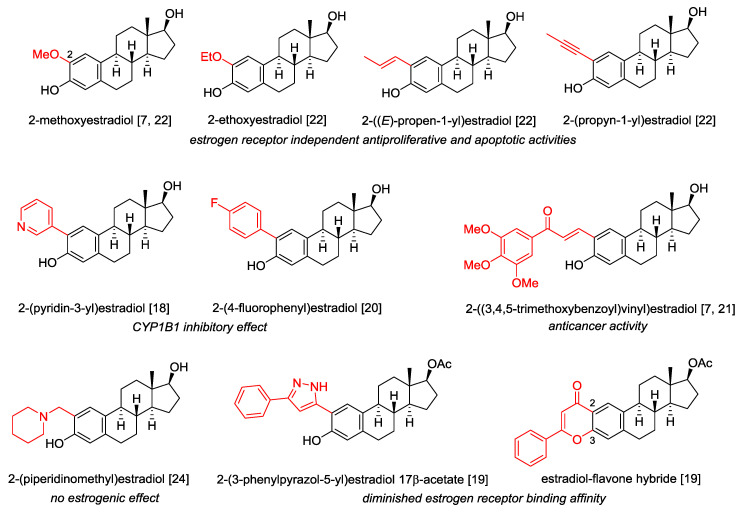
A-ring modified derivatives of estradiol displaying anticancer activity and/or reduced estrogenic action.

Since the functional groups responsible for hormone receptor binding are at C-3 and C-17 in the A- and D-rings of sex hormones, the chemical modifications made here or at nearby positions seem reasonable to eliminate the undesirable side effects. Interestingly, far fewer derivatives have been studied, mainly in the estrone series that contain a heterocyclic moiety attached to the A- instead of the D-ring [18,19]. However, a number of estradiol derivatives having a simple substituent at C-2, including the best-known 2-methoxyestradiol, have been demonstrated to display cytotoxic activity exempt from hormonal effects (Figure 1) [7,20,21,22]. The low hormone receptor-binding affinity of these ligands can be attributed to steric and electronic factors, altered pK_a_ value and disrupted intermolecular H-bonding to the target protein of the phenolic A-ring [23,24]. The incorporation of a heterocycle condensed to the 2,3-position of the estrane skeleton, which permanently abolishes the acidic and H-donor phenolic OH group, is further expected to result in compounds with diminished estrogenic activity. Surprisingly, there are only a few literature examples of this type of compound (Figure 1) [19] in spite of the excellent reactivity of phenols, especially in aromatic electrophilic substitution reactions, which may serve as a basis for heterocycle construction.

In recent years, considerable attention has been devoted to the synthesis of sex hormone-derived pyrazoles, which have proved to be the most promising of all heterocyclic derivatives for inhibiting one of the enzymes of steroidogenesis or influencing the cell cycle and inducing apoptosis in tumor cells [16,25,26,27,28,29,30]. The introduction of such a structural element into the 2-position of estradiol may open new perspectives in the research of anticancer agents, as the pyrazole ring is a very common building block in non-steroidal anticancer compounds as well [31,32]. In addition, the incorporation of a formyl functionalized pyrazole moiety allows intramolecular cyclization with the phenolic 3-OH group of the A-ring, leading to domain-integrated pyrazolocoumarin-estradiol hybrid compounds. Both coumarins and pyrazolocoumarins are valuable heterocyclic units in a number of marketed drugs and experimental agents with anticancer, antimicrobial, and anti-inflammatory activities [33,34,35].

Given the aforementioned literature background, the aim of the current study was to develop an efficient synthetic route for the preparation of novel pyrazole-containing estradiol derivatives by modifying the phenolic A-ring, and thus to extend the chemical space towards the synthesis and investigation of a less studied family of compounds. Structurally similar molecules without C- and D-rings of the sterane core starting from 5,6,7,8-tetrahydro-2-naphthol were also synthesized for pharmacological comparison. To evaluate the anticancer efficiency of the new derivatives, their cytotoxic activity was screened in vitro on two prostate (DU145, PC-3) and two gynecological (HeLa, MCF-7) cell lines as well as on non-cancerous fibroblasts (MRC-5) using MTT assays [36]. Several compounds showing cancer cell-specific cytotoxicity were identified and the half maximal inhibitory concentrations for the most promising compounds were finally determined.

## 2. Results and Discussion

### 2.1. Syntheses

**Scheme 1:**
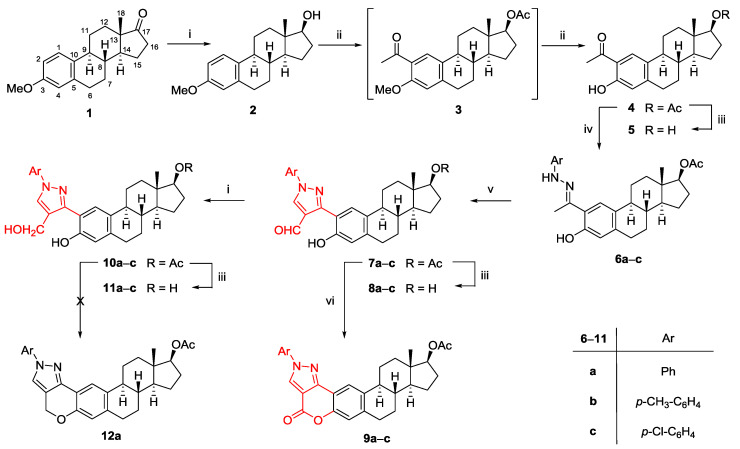
Multistep synthesis of arylpyrazole derivatives and pyrazolocoumarin hybrids of estradiol. *Reagents and conditions:* (i) NaBH_4_, EtOH, rt, 2 h; (ii) AcCl, AlCl_3_ (4 equiv.), DCM, N_2_ atm., 0→25 °C, 4 h; (iii) KOH, MeOH, rt, 2 h (for **4** and **7a**–**c**) or 40 °C, 3 h (for **10a**–**c**); (iv) Ar-NHNH_2_⋅HCl, NaOAc, EtOH, MW, 100 °C, 30 min; (v) POCl_3_, DMF, 0 °C, 30 min, then 60 °C, 3 h (for **6a** and **6b**) or 4 h (for **6c**); (vi) Jones reagent, acetone, rt, 10 min.

For the synthesis of A-ring-modified pyrazole derivatives, 2-acetyl estradiol 17β-acetate (**4**) was first prepared as starting material. Friedel-Crafts regioselective *ortho*-acetylation of the aromatic ring of estradiol 3-methyl ether (**2**), obtained from its precursor **1** by simple reduction, was carried out under mild conditions using a one-pot procedure, further developing the available literature method [37] (Scheme 1). Estradiol was previously reported to undergo diacetylation during treatment with acetyl chloride (AcCl), and AlCl_3_ was found not to be a strong enough Lewis acid to initiate a Fries rearrangement of the resulting diacetate to compound **4**; thus, the more expensive and moisture-sensitive ZrCl_4_ had to be used [38,39]. However, it was also demonstrated by Bubert et al. that the acetylation could be performed in the presence of AlCl_3_ without difficulty when the phenolic OH of estradiol was protected as a methyl ether (**2**) [37]. Thanks to the strong *ortho*-directing effect of the OMe group and because of the sterically less hindered character of C-2 over C-4, the aromatic electrophilic substitution occurred regioselectively to furnish a 3-protected 2-acetyl derivative (**3**) in good yield. After purification, the aromatic methyl ether (**3**) was cleaved in boiling dichloromethane (DCM) using a chloroaluminate ionic liquid reagent [37]. According to our observation, however, the acetylation/deprotection sequence can be carried out from **2** with AlCl_3_ in a single step without isolating the 2-acetyl-3-methyl ether intermediate (**3**) (Scheme 1). Apart from the key role of low reaction temperature during acetylation in order to access high regioselectivity, the amount of the Lewis acid used was found to be of crucial importance; i.e., a twofold molar excess of AlCl_3_ compared to the steroid favored the rapid formation and isolation of **3**, while a fourfold excess was needed for complete conversion of **2** to **4** within 3 h at 0→25 °C. In contrast to the two-step transformation, which required two purification steps and an elevated temperature during deprotection, leading to the desired product (**4**) in a ca. 74% yield, our one-pot method proceeded under milder conditions in a shorter time, resulting in **4** in 80% yield after recrystallization. 

After optimization, the synthesis of the 2-acetylestradiol derivative (**4**) was repeated on a larger scale without significant changes to serve as starting material for heterocycle formation. Since the hydrazones of methyl ketones are suitable precursors of pyrazole-4-carbaldehydes upon treatment with the Vilsmeier-Haack reagent [26,40], compound **4** was next reacted with three arylhydrazines, namely phenylhydrazine, 4-tolylhydrazine, and 4-chlorophenylhydrazine, respectively, differing in the electron demand of their substituents on the aromatic ring (Scheme 1). The reagents were liberated from their stable hydrochloride salts with NaOAc, and the ethanolic mixtures were irradiated by microwave (MW) at 100 °C. Since the carbonyl-C of the acetophenone moiety has a diminished reactivity toward nucleophilic attack due to steric and electronic reasons, hydrazone formation was found to be very sluggish under conventional heating, and only moderate conversion was achieved during 5 h of reflux. However, the MW-assisted condensations took place within 30 min and the crystalline hydrazones (**6a**–**c**) could be filtered off from the mixtures in yields around 75% (Table 1, entries 1–3); higher conversions could not be gained in these cases even by increasing the reaction time. Although the nucleophilicity of the terminal nitrogen of the arylhydrazines is definitely affected by the electron-donating CH_3_ group or electron-withdrawing Cl atom on the aromatic ring, a substituent effect influencing the yield of the products could not be observed under MW conditions.

The crude hydrazones were next treated with the Vilsmeier–Haack reagent generated in situ from dimethylformamide (DMF) and POCl_3_ at 0 °C to afford 1-arylpyrazole-4-carbaldehydes (**7a**–**c**) in 72–78% yields via ring closure and simultaneous incorporation of two carbon atoms from the reagent [40] (Scheme 1 and Table 1, entries 1–3). The transformations were carried out at 60 °C for 3 h for **6a** and **6b**, while 4 h were needed for the conversion of **6c**. Considering the proposed mechanism of the Vilsmeier-Haack reaction of hydrazones [41], the lower reactivity of **6c** may be attributed to the electron-withdrawing effect of the Cl atom, which endows a molecule with reduced nucleophilic character against electrophilic attack. Thereafter, oxidative lactonization of **7a**–**c** with the Jones reagent in acetone occurred rapidly to give pyrazolocoumarin steroid hybrids **9a**–**c**, while reductive transformations led to **10**a–**c** in good yields. Moreover, intramolecular ether formation was also tried for **10a** under different conditions in order to obtain pyrazolopyran **12a**; however, all synthetic efforts failed. Contrarily, 17-OH analogs of **4**, **7a**–**c**, and **10a**–**c** were synthesized without difficulty in alkaline methanol. Since similar deacetylation of lactone **9a** did not result in a single product due to partial ring-opening and parallel methyl ester formation, the idea of preparing 17-OH derivatives was discarded.

**Scheme 2:**
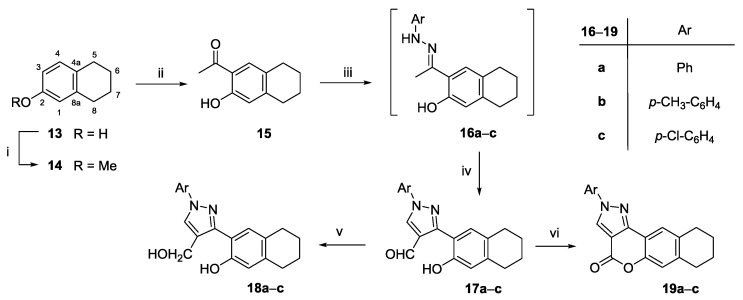
Multistep synthesis of non-steroidal arylpyrazole derivatives and pyrazolocoumarins. *Reagents and conditions:* (i) Me_2_SO_4_, K_2_CO_3_, acetone, reflux, 3 h; (ii) AcCl, AlCl_3_ (4 equiv.), DCM, N_2_ atm., 0→25 °C, 4 h; (iii) Ar-NHNH_2_⋅HCl, NaOAc, EtOH, MW, 100 °C, 20 min; (iv) POCl_3_, DMF, 0 °C, 30 min, then 60 °C, overnight; (v) NaBH_4_, EtOH, rt, 2 h; (vi) Jones reagent, acetone, reflux, 30 min (for **17b** and **17c**).

To gain insights into the synthetic and pharmacological differences and to find structure–activity relationships, the reaction sequence described above was also performed with 5,6,7,8-tetrahydro-2-naphthol (**13**), a simplified molecule that structurally mimics the A- and B-rings of the estrane backbone (Scheme 2). Friedel-Crafts acetylation of the protected starting material (**14**) occurred regioselectively to furnish **15** [42] in 69% yield after purification. In contrast to the similar reaction of the acetylated steroid **4**, the subsequent condensation reactions with arylhydrazines did not result in isolable hydrazones, although high conversions were observed by TLC monitoring; therefore, the intermediates (**16a**–**c**) were subjected to 4-formylpyrazole formation under Vilsmeier-Haack conditions. Consequently, the yield of products **17a**–**c** could only be calculated from the starting material (**15**) for the two consecutive steps. Although both transformations proceeded well, lower pyrazole yields (34–41%) were obtained compared to the reactions on steroids (54–61% for **4**) due to the impossibility of removing excess reagents after the first step, the difficulty of purification, and the formation of byproducts due to the longer reaction times needed for heterocyclizations (Table 1, entries 4–6). Reduction of the formyl group in the heteroaromatic ring of **17a**–**c** with NaBH_4_ in EtOH furnished hydroxymethyl-substituted derivatives **18a** and **18b** in good yields (>80%), while the presence of the electron-withdrawing Cl atom in **17c** allowed the formation of **18c** only in a moderate yield (66%). A similar substituent effect, albeit to a lesser extent, was also observed upon conversion of **7c** to **10c**. The behavior of **17a**–**c** under oxidative conditions was also found to differ from that of the analogous steroidal compounds (**7a**–**c**). The low solubility of **17a**–**c** in acetone at room temperature required heating of the mixtures, which led to the formation of unidentified byproducts, with reduced yields of the desired lactones **19a**–**c**.

Structural analyses of all synthesized compounds were carried out by NMR and ESI-MS measurements. Hydrazones **6a**–**c** and **16a**–**c** were not characterized but were transformed immediately after formation due to their instability and/or difficulty of isolation. However, the signals in the deshielded region (>6.8 ppm) of the ^1^H NMR spectra of **7a**–**c** and **17a**–**c** confirmed the 2,3-disubstituted character of the condensed aromatic A-ring (two singlets for 1-H and 4-H and the appearance of the phenolic OH), the heterocyclization (a singlet for 5′-H at around 8.5 ppm), and the incorporation of a formyl group into the pyrazole moiety (4′-CHO peak at around 10.2 ppm) under the Vilsmeier-Haack conditions (Table 2). The ^13^C NMR spectra and the determined molecular weights also provided evidence for the chemical structures. Reduction of the formyl group by NaBH_4_ led to derivatives (**10a**–**c** and **18a**–**c**) containing a hydroxymethyl group instead of a formyl group on their heteroaromatic ring, so instead of a signal of formyl proton, a singlet corresponding to two equivalent protons (CH_2_OH) could be observed around 4.86 ppm in the ^1^H NMR spectra. Oxidative lactonization is associated with the disappearance of both CHO and phenolic OH proton signals in **9a**–**c** and **19a**–**c**, while a negative carbonyl signal around 158.5 ppm in the ^13^C NMR spectra (J-MOD) confirmed the cyclic ester formation.

### 2.2. Evaluation of In Vitro AnticancerActivity

**Figure 2:**
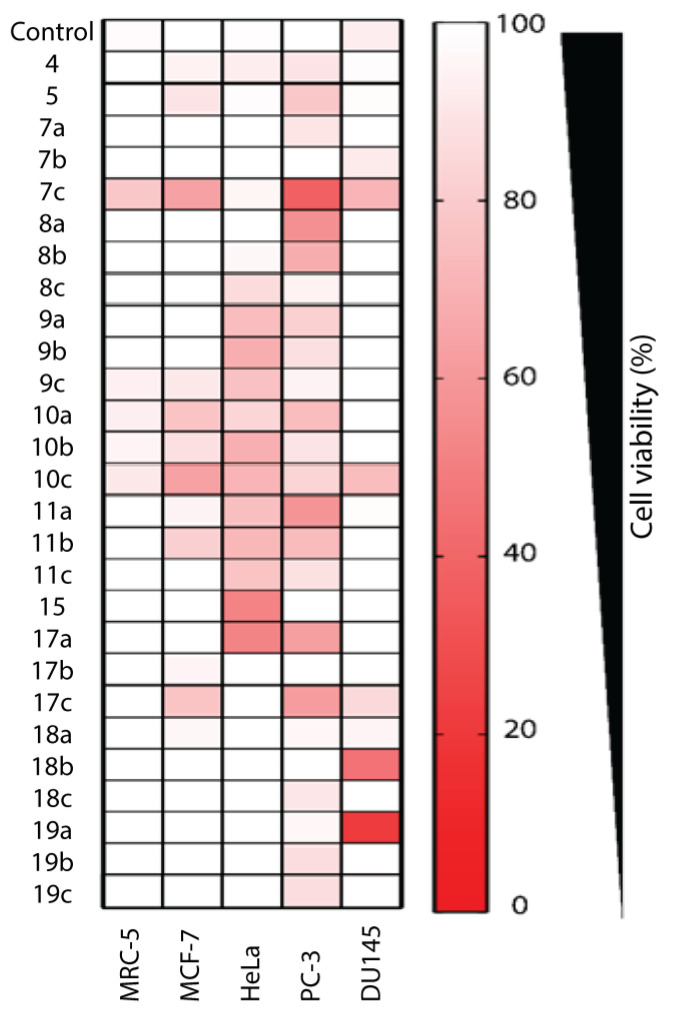
Heat map representation of the primary cytotoxic effects of novel A-ring-modified estradiol derivatives and their structurally simplified non-steroidal pairs on different cell lines (compound concentration = 2.5 μM; incubation time = 72 h). Control represents the viability of untreated cells.

Cytotoxicity screens were performed to assess the in vitro anticancer activity of the synthesized A-ring-linked 2-pyrazoles of estradiol, the pyrazolocoumarin-estradiol hybrids, and their structurally simplified non-steroidal analogs on various cancerous cell lines such as MCF-7, PC-3, DU145, and HeLa and on non-cancerous MRC-5 fibroblasts. The 2-acetyl-phenol starting materials (**4**, **5**, and **15**) were also included for comparison. All compounds were applied in 2.5 µM concentration for 72 h. The obtained cell viability data were used to construct a heat map (Figure 2) in order to select compounds that exert cancer cell line-specific cytotoxicity. Overall, 10–15 potential molecules (steroids and non-steroids) were found to be competent against cisplatin-resistant PC-3 and a similar number of compounds were active on HeLa cells (Figure 2). Furthermore, some compounds discriminated only DU145 cells and could eliminate these type of prostate cancer cells.

Based on the heat map, two steroidal 4-formylpyrazoles (**7c** and **8a**), the reduced derivative of the latter compound (**11a**), and some tetrahydronaphthol-derived molecules (**15**, **17a**, **17c**, **18b**, and **19a**) were identified that exhibited a minimum of 40% cytotoxicity on at least one cancer cell line and had no or very mild effect on non-cancerous fibroblasts. To understand structure-function relationships, we also included the structural analogs of the selected compounds, namely **7a** (C-17 acetate of **8a**), **8c** (C-17 OH counterpart of **7c**), and **9a** (steroidal pair of **19a**) into subsequent analyses. All these 11 compounds (**7a**, **7c**, **8a**, **8c**, **9a**, **11a**, **15**, **17a**, **17c**, **18b**, and **19a**) were further examined to determine their IC_50_ concentrations on all the cell lines previously mentioned and their efficacy was compared to the reference drug cisplatin. For this, compounds were applied on MCF-7, PC-3, DU145, and HeLa and on non-cancerous MRC-5 fibroblasts in various concentrations for 72 h or were treated with cisplatin at different concentrations. On the viability data, dose–response curves were fitted (Figure S1) and IC_50_ values were calculated accordingly (Table 3). In agreement with the primary cytotoxicity screen (Table S1), the obtained IC_50_ concentrations clearly indicated which compound was selectively effective on one or more cancer cell lines. Non-steroid small molecules **15** and **19a** were very potent and selective on HeLa or DU145 cells, respectively; however, the steroidal compounds **8a** and **8c** exhibited a similar selective effect on PC-3 and on HeLa cells, respectively. On the other hand, we identified compounds, like **7c**, which were effective on three cancer cell lines, i.e., on PC-3, DU145, and MCF-7 cells, in significantly lower concentrations than cisplatin, albeit affecting non-cancerous fibroblasts as well. When the activity of structurally related molecules was compared, we realized that often the simplified small molecules themselves were able to induce significant toxicity (**17a**, **17c**); however, when the same structural motif was incorporated into an estrane backbone, the resulting compounds were either more effective on the cancer cells (**8a**, PC-3) or were able to target a different cell line (**8c**, HeLa). Typically, acetylated steroidal pyrazoles were less favorable than deacetylated counterparts due to reduced cancer cell selectivity (**7c**, **10a**). As a whole, potent and tumor cell-selective compounds were found on cervical and prostate cancer cell lines (**9a**—HeLa, **11a**—PC-3, and **19a**—DU145), each of which is a phenyl-containing derivative on the pyrazole ring, i.e., the substitution of the aromatic ring proved to be unfavorable in terms of biological effect or selectivity.

## 3. Materials and Methods

### 3.1. Chemistry

#### 3.1.1. General

Chemicals, reagents, and solvents were purchased from commercial suppliers (Sigma-Aldrich Corporation, St. Louis, MO, USA; TCI, Tokyo, Japan and Alfa Aesar, Haverhill, MA, USA) and used without further purification. Melting points (Mp) were determined on an SRS Optimelt digital apparatus (Stanford Research Systems Inc., Sunnyvale, CA, USA) and are uncorrected. For MW-assisted syntheses, a CEM Discover SP (CEM Corporation, Matthews, NC, USA) laboratory MW reactor was used with a maximum power of 200 W (running a dynamic control program). Elementary analysis data were obtained with a PerkinElmer CHN analyzer model 2400 (PerkinElmer Inc, Waltham, MA, USA). The transformations were monitored by TLC using 0.25 mm thick Kieselgel-G plates (Si 254 F, Merck KGaA, Darmstadt, Germany). The compound spots were detected by spraying with 5% phosphomolybdic acid in 50% aqueous phosphoric acid. Flash chromatographic purifications were carried out on silica gel 60, 40–63 μm (Merck KGaA, Darmstadt, Germany). NMR spectra were recorded with a Bruker DRX 500 (Bruker, Billerica, MA, USA) instrument at room temperature in CDCl_3_ or DMSO-*d*_6_ using residual solvent signals as an internal reference. Chemical shifts are reported in ppm (*δ* scale), and coupling constants (*J*) are given in Hz. Multiplicities of the ^1^H signals are indicated as a singlet (s), a broad singlet (bs), a doublet (d), a triplet (t), or a multiplet (m). ^13^C NMR spectra are ^1^H-decoupled and the J-MOD pulse sequence was used for multiplicity editing. In this spin-echo type experiment, the signal intensity is modulated by the different coupling constants *J* of carbons depending on the number of attached protons. Both protonated and unprotonated carbons can be detected (CH_3_ and CH carbons appear as positive signals, while CH_2_ and C carbons as negative signals).

Automated flow injection analyses were performed with an HPLC/MSD system. System accessories: a micro-well plate autoinjector, an Agilent 1100 micro vacuum degasser (Agilent Technologies, Santa Clara, CA, USA), a quaternary pump, and a 1946A MSD equipped with an electrospray ion source (ESI) operated in positive ion mode. ESI parameters were: nebulizing gas N_2_, at 35 psi; drying gas N_2_, at 350 °C and 12 L/min; capillary voltage 3000 V; fragmentor voltage 70 V. The MSD was operated with a mass range of *m/z* 60−620 in scan mode. Samples (0.2 μL) were injected directly into the solvent flow (0.3 mL/min) of acetonitrile/H_2_O = 70:30 (*v*/*v*) with the simultaneous addition of 0.1% formic acid with an automated needle wash. Agilent LC/MSD Chemstation (C.01.08, Agilent Technologies Inc., Santa Clara, CA, USA) was used as software to control the system.

#### 3.1.2. General Procedure for the Friedel-Crafts Acetylation/Demethylation of Estradiol 3-Methyl Ether (**2**) or 6-Methoxy-1,2,3,4-Tetrahydronaphthalene (**14**)

Anhydrous AlCl_3_ (10.5 g, 78.6 mmol) was suspended in dry CH_2_Cl_2_ (75 mL) under N_2_ atmosphere, and the mixture was cooled to 0 °C. Acetyl chloride (3.1 mL, 43.6 mmol) was added slowly, then the mixture was stirred for 15 min. A solution of **2** (5 g, 17.5 mmol) or **14** (2.6g, 17.6 mmol) in dry CH_2_Cl_2_ (25 mL) was added dropwise to the mixture over a period of 10 min, then stirred for 15 min at 0 °C, after which it was allowed to warm to room temperature and stirred for another 3 h. The reaction mixture was poured onto crushed ice and stirred vigorously for 10 min. The organic layer was separated, and the remaining aqueous layer was extracted with CH_2_Cl_2_ (2 × 50 mL). The combined organic layers were washed with brine, dried with anhydrous Na_2_SO_4_, and concentrated in vacuo.

2-Acetyl-estra-1,3,5(10)-triene-3,17β-diol-17-acetate (**4**). The crude product was recrystallized from MeOH. Yield: 4.95 g (80%, white solid); Mp 200–202 °C (198–200 °C [37]); Anal. Calcd. for C_22_H_28_O_4_ (356.46) C, 74.13; H, 7.92; O, 17.95. Found C 74.25; H 7.86. ^1^H NMR (CDCl_3_, 500 MHz): *δ* 0.84 (s, 3H, 18-CH_3_), 1.27–1.58 (overlapping m, 7H), 1.76 (m, 1H), 1.87–1.92 (overlapping m, 2H), 2.07 (s, 3H, AcO-CH_3_), 2.15–2.32 (overlapping m, 3H), 2.60 (s, 3H, 2-Ac-CH_3_), 2.87 (m, 2H, 6-H_2_), 4.70 (t, 1H, *J* = 8.5 Hz, 17-H), 6.69 (s, 1H, 4-H), 7.59 (s, 1H, 1-H), 12.04 (s, 1H, OH); ^13^C NMR (CDCl_3_, 125 MHz): *δ* 12.2 (C-18), 21.4 (AcO-CH_3_), 23.4 (CH_2_), 26.3 (CH_2_), 26.7 (2-Ac-CH_3_), 26.9 (CH_2_), 27.7 (CH_2_), 30.0 (CH_2_), 36.8 (CH_2_), 38.4 (CH), 43.0 (C-13), 43.5 (CH), 49.9 (CH), 82.7 (C-17), 117.7 (C-4), 118.0 (C-2), 127.4 (C-1), 131.5 (C-10), 147.3 (C-5), 160.2 (C-3), 171.4 (AcO-CO), 204.2 (2-Ac-CO); ESI-MS 357 [M + H]^+^.

3-Acetyl-5,6,7,8-tetrahydro-2-naphthol (**15**). The crude product was purified by column chromatography with hexane/CH_2_Cl_2_ = 1:1. Yield: 2.29 g (69%, white solid); Mp 70−72 °C (71−72 °C [42]); Anal. Calcd. for C_12_H_14_O_2_ (190.24) C 75.76; H 7.42. Found C 75.62; H 7.35. ^1^H NMR (CDCl_3_, 500 MHz): *δ* 1.78 (t-like m, 4H, 6-H_2_ and 7-H_2_), 2.59 (s, 3H, Ac-CH_3_), 2.71 (m, 2H) and 2.76 (m, 2H): 5-H_2_ and 8-H_2_, 6.67 (s, 1H, 1-H), 7.40 (s, 1H, 4-H), 11.97 (s, 1H, OH); ^13^C NMR (CDCl_3_, 125 MHz): *δ* 22.8 and 23.3: C-6 and C-7, 26.7 (Ac-CH_3_), 28.7 and 30.1: C-5 and C-8, 117.7 (C-1), 118.1 (C-3), 128.0 (C-4a), 131.0 (C-4), 147.6 (C-8a), 160.0 (C-2), 204.1 (Ac-CO); ESI-MS 191 [M + H]^+^.

#### 3.1.3. General Procedure for the MW-Assisted Synthesis of Arylhydrazones (**6a**–**c**) and Their Conversion to Steroidal 4-Formylpyrazoles (**7a**–**c**) under Vilsmeier–Haack Conditions

To a solution of **4** (713 mg, 2.0 mmol) or **15** (380 mg, 2.0 mmol) in EtOH (5 mL), anhydrous NaOAc (246 mg, 3.0 mmol) and (*p*-substituted) phenylhydrazine hydrochloride (3.0 mmol) were added, and the mixture was irradiated at 100 °C for 30 min in a closed tube. Hydrazones **6a**–**c** can be obtained by chilling the reaction vessel, filtering off the yellow precipitate and washing it with ice-cold methanol. **16a**–**c** could not be isolated; thus, the reaction mixture was evaporated to dryness and was used as-is in the next step. POCl_3_ (10.73 mmol, 1.0 mL) was added to DMF (10 mL) cooled to 0 °C. After stirring for 15 min, a solution of **6a**–**c** or **16a**–**c** (obtained in the previous step) in DMF (5 mL) was added dropwise to the reaction mixture and was then heated to 60 °C and stirred for another 3 h (**7a**–**c**) or 16 h (**17a**–**c**). The mixture was then poured onto crushed ice, extracted with EtOAc (3 × 25 mL), and the combined organic phases were washed with brine, dried with anhydrous Na_2_SO_4_, and concentrated in vacuo. The crude products were purified by column chromatography with EtOAc/CH_2_Cl_2_ = 1:99 (**7a**–**c**) or hexane/CH_2_Cl_2_ = 2:8 (**17a**–**c**).

##### 2-(4′-Formyl-1′-phenyl-1′*H*-pyrazol-3′-yl)-estra-1,3,5(10)-triene-3,17β-diol-17-acetate (**7a**)

According to the general procedure, phenylhydrazine hydrochloride (435 mg) was used. Yield of the phenylhydrazone (**6a**, yellow solid): 678 mg (76%). Yield of **7a** (yellow solid): 550 mg (75%). Mp 230−232 °C; Anal. Calcd. for C_30_H_32_N_2_O_4_ (484.60) C 74.36; H 6.66. Found C 74.48; H 6.52. ^1^H NMR (CDCl_3_, 500 MHz): *δ* 0.85 (s, 3H, 18-CH_3_), 1.26–1.61 (overlapping m, 7H), 1.76 (m, 1H), 1.92 (m, 2H), 2.07 (s, 3H, Ac-CH_3_), 2.20–2.30 (overlapping m, 2H), 2.39 (m, 1H), 2.90 (m, 2H, 6-H_2_), 4.69 (t, 1H, *J* = 8.4 Hz, 17-H), 6.83 (s, 1H, 4-H), 7.43 (t-like m, 1H, 4″-H), 7.54 (t-like m, 2H, 3″-H and 5″-H), 7.88 (d, 2H, *J* = 7.9 Hz, 2″-H and 6″-H), 7.88 (s, 1H, 1-H), 8.56 (s, 1H, 5′-H), 9.84 (bs, 1H, 3-OH), 10.19 (s, 1H, CHO); ^13^C NMR (CDCl_3_, 125 MHz): *δ* 12.2 (C-18), 21.4 (Ac-CH_3_), 23.4 (CH_2_), 26.5 (CH_2_), 27.3 (CH_2_), 27.8 (CH_2_), 29.7 (CH_2_), 36.9 (CH_2_), 38.6 (CH), 43.0 (C-13), 43.9 (CH), 49.9 (CH), 82.9 (C-17), 113.2 (C-4′), 117.1 (C-4), 119.7 (2C, C-2″ and C-6″), 123.1 (C-2), 126.9 (C-1), 128.5 (C-4″), 130.0 (2C, C-3″ and C-5″), 132.2 (C-10), 132.7 (C-5′), 138.4 (C-1″), 140.7 (C-5), 153.2 (C-3′), 153.8 (C-3), 171.4 (Ac-CO), 184.5 (CHO); ESI-MS 485 [M + H]^+^.

##### 2-(4′-Formyl-1′-(4″-tolyl)-1′*H*-pyrazol-3′-yl)-estra-1,3,5(10)-triene-3,17β-diol-17-acetate (**7b**)

According to the general procedure, 4-tolylhydrazine hydrochloride (475 mg) was used. Yield of the 4-tolylhydrazone (**6b**, yellow solid): 717 mg (78%). Yield of **7b** (white solid): 607 mg (78%). Mp 229−231 °C; Anal. Calcd. for C_31_H_34_N_2_O_4_ (498.62) C 74.67; H 6.87. Found C 74.53; H 6.99. ^1^H NMR (CDCl_3_, 500 MHz): *δ* 0.85 (s, 3H, 18-CH_3_), 1.27–1.59 (overlapping m, 7H), 1.76 (m, 1H), 1.91 (m, 2H), 2.07 (s, 3H, Ac-CH_3_), 2.20–2.29 (overlapping m, 2H), 2.39 (m, 1H), 2.43 (s, 3H, 4″-CH_3_), 2.90 (m, 2H, 6-H_2_), 4.69 (t, 1H, *J* = 8.4 Hz, 17-H), 6.82 (s, 1H, 4-H), 7.32 (d, 2H, *J* = 8.4 Hz, 3″-H and 5″-H), 7.59 (d, 2H, *J* = 8.8 Hz, 2″-H and 6″-H), 7.88 (s, 1H, 1-H), 8.51 (s, 1H, 5′-H), 9.89 (bs, 1H, 3-OH), 10.17 (s, 1H, CHO); ^13^C NMR (CDCl_3_, 125 MHz): *δ* 12.2 (C-18), 21.2 (4″-CH_3_), 21.4 (Ac-CH_3_), 23.4 (CH_2_), 26.4 (CH_2_), 27.3 (CH_2_), 27.8 (CH_2_), 29.7 (CH_2_), 36.9 (CH_2_), 38.6 (CH), 43.0 (C-13), 43.9 (CH), 49.9 (CH), 82.9 (C-17), 113.2 (C-4′), 117.1 (C-4), 119.6 (2C, C-2″ and C-6″), 122.9 (C-2), 126.9 (C-1), 130.5 (2C, C-3″ and C-5″), 132.1 (C-10), 132.6 (C-5′), 136.1 (C-4″), 138.6 (C-1″), 140.6 (C-5), 153.0 (C-3′), 153.8 (C-3), 171.4 (Ac-CO), 184.5 (CHO); ESI-MS 499 [M + H]^+^.

##### 2-(1′-(4″-Chlorophenyl)-4′-formyl-1′H-pyrazol-3′-yl)-estra-1,3,5(10)-triene-3,17β-diol-17-acetate (**7c**)

According to the general procedure, 4-chlorophenylhydrazine hydrochloride (539 mg) was used. Yield of the 4-chlorophenylhydrazone (**6c**, white solid): 722 mg (75%). Yield of **7c** (white solid): 560 mg (72%). Mp 268–270 °C; Anal. Calcd. for C_30_H_31_ClN_2_O_4_ (519.04) C 69.42; H 6.02. Found C 69.29; H 6.11. ^1^H NMR (CDCl_3_, 500 MHz): *δ* 0.85 (s, 3H, 18-CH_3_), 1.26–1.59 (overlapping m, 7H), 1.76 (m, 1H), 1.92 (m, 2H), 2.07 (s, 3H, Ac-CH_3_), 2.20–2.29 (overlapping m, 2H), 2.37 (m, 1H), 2.90 (m, 2H, 6-H_2_), 4.69 (t, 1H, *J* = 8.4 Hz, 17-H), 6.83 (s, 1H, 4-H), 7.51 (d, 2H, *J* = 8.8 Hz, 3″-H and 5″-H), 7.67 (d, 2H, *J* = 8.8 Hz, 2″-H and 6″-H), 7.84 (s, 1H, 1-H), 8.53 (s, 1H, 5′-H), 9.65 (bs, 1H, 3-OH), 10.18 (s, 1H, CHO); ^13^C NMR (CDCl_3_, 125 MHz): δ 12.2 (C-18), 21.4 (Ac-CH_3_), 23.4 (CH_2_), 26.5 (CH_2_), 27.2 (CH_2_), 27.8 (CH_2_), 29.7 (CH_2_), 36.9 (CH_2_), 38.6 (CH), 43.0 (C-13), 43.8 (CH), 49.9 (CH), 82.9 (C-17), 113.0 (C-4′), 117.2 (C-4), 120.9 (2C, C-2″ and C-6″), 123.3 (C-2), 126.9 (C-1), 130.2 (2C, C-3″ and C-5″), 132.3 (C-10), 132.6 (C-5′), 134.3 (C-4″), 136.9 (C-1″), 140.9 (C-5), 153.4 (C-3′), 153.7 (C-3), 171.4 (Ac-CO), 184.5 (CHO); ESI-MS 519 [M + H]^+^.

##### 3′-(3-Hydroxy-5,6,7,8-tetrahydronaphthalen-2-yl)-1′-phenyl-1′H-pyrazole-4′-carbaldehyde (**17a**)

According to the general procedure, phenylhydrazine hydrochloride (435 mg) was used. Phenylhydrazone (**16a**) could not be isolated. Yield of **17a** (white solid): 242 mg (38%). Mp 137–138 °C; Anal. Calcd. for C_20_H_18_N_2_O_2_ (318.38) C 75.45; H 5.70. Found C 75.32; H 5.55. ^1^H NMR (CDCl_3_, 500 MHz): *δ* 1.82 (t-like m, 4H, 6-H_2_ and 7-H_2_), 2.79 (d-like m, 5-H_2_ and 8-H_2_), 6.83 (s, 1H, 4-H), 7.42 (t-like m, 1H, 4″-H), 7.53 (m, 3H, 1-H, 3″-H and 5″-H), 7.72 (d, 2H, *J* = 7.7 Hz, 2″-H and 6″-H), 8.56 (s, 1H, 5′-H), 9.68 (bs, 1H, 3-OH), 10.20 (s, 1H, CHO); ^13^C NMR (CDCl_3_, 125 MHz): *δ* 23.1 and 23.5: C-6 and C-7, 28.8 and 29.6: C-5 and C-8, 113.3 (C-4′), 117.2 (C-4), 119.7 (2C, C-2″ and C-6″), 123.0 (C-2), 128.5 (C-1), 128.8 (C-8a), 130.0 (2C, C-3″ and C-5″), 130.1 (C-4″), 132.0 (C-5′), 138.4 (C-1″), 140.7 (C-4a), 153.1 (C-3′), 153.6 (C-3), 184.9 (CHO); ESI-MS 319 [M + H]^+^.

##### 3′-(3-Hydroxy-5,6,7,8-tetrahydronaphthalen-2-yl)-1′-(4″-tolyl)-1′H-pyrazole-4′-carbaldehyde (**17b**)

According to the general procedure, 4-tolylhydrazine hydrochloride (475 mg) was used. 4-tolylhydrazone (**16b**) could not be isolated. Yield of **17b** (white solid): 273 mg (41%). Mp 151–153 °C; Anal. Calcd. for C_21_H_20_N_2_O_2_ (332.40) C 75.88; H 6.06. Found C 76.02; H 5.95. ^1^H NMR (CDCl_3_, 500 MHz): *δ* 1.82 (t-like m, 4H, 6-H_2_ and 7-H_2_), 2.42 (s, 3H, 4″-CH_3_), 2.79 (d-like m, 5-H_2_ and 8-H_2_), 6.82 (s, 1H, 4-H), 7.32 (d, 2H, *J* = 8.5 Hz, 3″-H and 5″-H), 7.55 (s, 1H, 1-H), 7.59 (d, 2H, *J* = 8.9 Hz, 2″-H and 6″-H), 8.50 (s, 1H, 5′-H), 9.69 (bs, 1H, 3-OH), 10.19 (s, 1H, CHO); ^13^C NMR (CDCl_3_, 125 MHz): *δ* (4″-CH_3_), 23.2 and 23.6: C-6 and C-7, 28.8 and 29.7: C-5 and C-8, 113.5 (C-4′), 117.2 (C-4), 119.7 (2C, C-2″ and C-6″), 122.9 (C-2), 128.8 (C-8a), 130.1 (C-1), 130.5 (2C, C-3″ and C-5″), 131.9 (C-5′), 136.2 (C-4″), 138.6 (C-1″), 140.9 (C-4a), 153.0 (C-3′), 153.7 (C-3), 184.8 (CHO); ESI-MS 333 [M + H]^+^.

##### 1′-(4″-Chlorophenyl)-3′-(3-hydroxy-5,6,7,8-tetrahydronaphthalen-2-yl)-1′H-pyrazole-4″-carbaldehyde (**17c**)

According to the general procedure, 4-chlorophenylhydrazine hydrochloride (540 mg) was used. 4-chlorophenylhydrazone (**16c**) could not be isolated. Yield of **17c** (white solid): 240 mg (34%). Mp 182–184 °C; Anal. Calcd. for C_20_H_17_ClN_2_O_2_ (352.82) C 68.09; H 4.86. Found C 67.95; H 4.95. ^1^H NMR (CDCl_3_, 500 MHz): *δ* 1.82 (t-like m, 4H, 6-H_2_ and 7-H_2_), 2.79 (d-like m, 5-H_2_ and 8-H_2_), 6.82 (s, 1H, 4-H), 7.49 (d, 2H, *J* = 8.9 Hz, 3″-H and 5″-H), 7.51 (s, 1H, 1-H), 7.67 (d, 2H, *J* = 8.9 Hz, 2″-H and 6″-H), 8.52 (s, 1H, 5′-H), 9.45 (bs, 1H, 3-OH), 10.18 (s, 1H, CHO); ^13^C NMR (CDCl_3_, 125 MHz): δ 23.1 and 23.5: C-6 and C-7, 28.8 and 29.7: C-5 and C-8, 113.3 (C-4′), 117.3 (C-4), 120.9 (2C, C-2″ and C-6″), 123.4 (C-2), 129.0 (C-8a), 130.1 (C-1), 130.2 (2C, C-3″ and C-5″), 132.0 (C-5′), 134.3 (C-4″), 137.0 (C-1″), 141.3 (C-4a), 153.4 (C-3′), 153.6 (C-3), 184.8 (CHO); ESI-MS 353 [M + H]^+^.

#### 3.1.4. General Procedure for the Reduction of Steroidal and Non-Steroidal 4-Formylpyrazoles (**7a**–**c** and **17a**–**c**)

4-formylpyrazole (**7a**–**c**, 0.30 mmol or **17a**–**c**, 0.50 mmol) was suspended in EtOH (10 mL), and NaBH_4_ (45 mg, 1.20 mmol) was added. The solution was stirred at room temperature for 2 h, then poured into water and neutralized with diluted HCl. The resulting precipitate was filtered, washed with water, dried, and purified by flash chromatography if necessary.

##### 3-(4′-(Hydroxymethyl)-1′-phenyl-1′H-pyrazol-3′-yl)-estra-1,3,5(10)-triene-3,17β-diol-17-acetate (**10a**)

According to the general procedure, compound **7a** (145 mg) was used. After purification with EtOAc/CH_2_Cl_2_ = 5:95 as eluent, 10a was obtained as a white solid (127 mg, 87%). Mp 257–259 °C; Anal. Calcd. for C_30_H_34_N_2_O_4_ (486.61) C 74.05; H 7.04. Found C 73.91; H 7.18. ^1^H NMR (CDCl_3_, 500 MHz): *δ* 0.84 (s, 3H, 18-CH_3_), 1.28–1.57 (overlapping m, 7H), 1.76 (m, 1H), 1.90 (m, 2H), 2.07 (s, 3H, Ac-CH_3_), 2.20–2.27 (overlapping m, 2H), 2.37 (m, 1H), 2.87 (m, 2H, 6-H_2_), 4.69 (t, 1H, *J* = 8.4 Hz, 17-H), 4.87 (s, 2H, CH_2_-OH), 6.80 (s, 1H, 4-H), 7.31 (t-like m, 1H, 4″-H), 7.47 (t-like m, 2H, 3″-H and 5″-H), 7.65 (d, 2H, *J* = 8.0 Hz, 2″-H and 6″-H), 7.68 (s, 1H, 1-H), 8.02 (s, 1H, 5′-H), 10.56 (3-OH); ^13^C NMR (CDCl_3_, 125 MHz): *δ* 12.2 (C-18), 21.4 (Ac-CH_3_), 23.4 (CH_2_), 26.4 (CH_2_), 27.4 (CH_2_), 27.7 (CH_2_), 29.6 (CH_2_), 37.1 (CH_2_), 38.7 (CH), 43.0 (C-13), 44.0 (CH), 49.9 (CH), 56.7 (CH_2_-OH), 82.9 (C-17), 114.4 (C-2), 116.8 (C-4), 118.7 (2C, C-2″ and C-6″), 120.9 (C-4′), 125.0 (C-4″), 126.9 (C-1), 127.9 (C-5′), 129.8 (2C, C-3″ and C-5″), 131.6 (C-10), 138.8 (C-5), 139.1 (C-1″), 150.6 (C-3′), 153.9 (C-3), 171.4 (Ac-CO); ESI-MS 487 [M + H]^+^.

##### 3-(4′-(Hydroxymethyl)-1′-(4″-tolyl)-1′H-pyrazol-3′-yl)-estra-1,3,5(10)-triene-3,17β-diol-17-acetate (**10b**)

According to the general procedure, compound **7b** (150 mg) was used. After purification with EtOAc/CH_2_Cl_2_ = 5:95 as eluent, **10b** was obtained as a white solid (128 mg, 85%). Mp 254–256 °C; Anal. Calcd. for C_31_H_36_N_2_O_4_ (500.64) C 74.37; H 7.25. Found C 74.50; H 7.32. ^1^H NMR (CDCl_3_, 500 MHz): *δ* 0.84 (s, 3H, 18-CH_3_), 1.26–1.57 (overlapping m, 7H), 1.74 (m, 1H), 1.89 (m, 2H), 2.06 (s, 3H, Ac-CH_3_), 2.16–2.25 (m, 2H), 2.36 (m, 1H), 2.39 (s, 3H, 4″-CH_3_), 2.87 (m, 2H, 6-H_2_), 4.68 (t, 1H, *J* = 8.5 Hz, 17-H), 4.84 (d-like m, 2H, CH_2_OH), 6.79 (s, 1H, 4-H), 7.26 (d, 2H, *J* = 8.4 Hz, 3″-H and 5″-H), 7.52 (d, 2H, *J* = 8.4 Hz, 2″-H and 6″-H), 7.67 (s, 1H, 1-H), 7.96 (s, 1H, 5′-H); ^13^C NMR (CDCl_3_, 125 MHz): *δ* 12.2 (C-18), 21.1 (4″-CH_3_), 21.4 (Ac-CH_3_), 23.4 (CH_2_), 26.3 (CH_2_), 27.3 (CH_2_), 27.7 (CH_2_), 29.6 (CH_2_), 37.0 (CH_2_), 38.7 (CH), 43.0 (C-13), 44.0 (CH), 49.9 (CH), 56.7 (CH_2_-OH), 82.9 (C-17), 114.5 (C-2), 116.8 (C-4), 118.7 (2C, C-2″ and C-6″), 120.6 (C-4′), 125.0 (C-1), 127.8 (C-5′), 130.2 (2C, C-3″ and C-5″), 131.6 (C-4″), 136.8 (C-10), 136.9 (C-1″), 138.7 (C-5), 150.2 (C-3′), 153.8 (C-3), 171.4 (Ac-CO); ESI-MS 501 [M + H]^+^.

##### 3-(1′-(4″-chlorophenyl)-4′-(hydroxymethyl)-1′H-pyrazol-3′-yl)-estra-1,3,5(10)-triene-3,17β-diol-17-acetate (**10c**)

According to the general procedure, compound **7c** (156 mg) was used. After purification with EtOAc/CH_2_Cl_2_ = 5:95 as eluent, **10c** was obtained as a white solid (120 mg, 77%). Mp 288–290 °C; Anal. Calcd. for C_30_H_33_ClN_2_O_4_ C 69.15; H 6.38. Found C 69.03; H 6.27. ^1^H NMR (DMSO-*d*_6_, 500 MHz): *δ* 0.79 (s, 3H, 18-CH_3_), 1.28–1.43 (overlapping m, 6H), 1.49 (m, 1H), 1.69 (m, 1H), 1.77 (m, 1H), 1.82 (m, 1H), 2.01 (s, 3H, Ac-CH_3_), 2.11 (m, 1H), 2.19 (m, 1H), 2.30 (m, 1H), 2.79 (m, 2H, 6-H_2_), 4.51 (d-like m, 2H, CH_2_OH), 4.61 (t, 1H, *J* = 8.4 Hz, 17-H), 5.15 (t-like m, 1H, CH_2_OH), 6.65 (s, 1H, 4-H), 7.56 (s, 1H, 1-H), 7.57 (d, 2H, *J* = 8.9 Hz, 3″-H and 5″-H), 7.87 (d, 2H, *J* = 8.9 Hz, 2″-H and 6″-H), 8.54 (s, 1H, 5′-H), 9.93 (3-OH); ^13^C NMR (DMSO-*d*_6_, 125 MHz): *δ* 12.0 (C-18), 20.9 (Ac-CH_3_), 22.8 (CH_2_), 25.8 (CH_2_), 26.8 (CH_2_), 27.2 (CH_2_), 28.9 (CH_2_), 36.5 (CH_2_), 38.2 (CH), 42.5 (C-13), 43.3 (CH), 49.1 (CH), 54.7 (CH_2_-OH), 81.9 (C-17), 115.5 (C-2), 115.8 (C-4), 119.6 (2C, C-2″ and C-6″), 123.2 (C-4′), 126.5 (C-1), 128.3 (C-5′), 129.6 (2C, C-3″ and C-5″), 130.2 and 130.8: C-4″ and C-10, 137.9 (2C): C-5 and C-1″, 150.0 (C-3′), 152.9 (C-3), 171.4 (Ac-CO); ESI-MS 521 [M + H]^+^.

##### 3-(4′-(Hydroxymethyl)-1′-phenyl-1′H-pyrazol-3′-yl)-5,6,7,8-tetrahydro-2-naphthol (**18a**)

According to the general procedure, compound **17a** (159 mg) was used. After purification with EtOAc/CH_2_Cl_2_ = 2:98 as eluent, **18a** was obtained as a white solid (133 mg, 83%). Mp 135–137 °C; Anal. Calcd. for C_20_H_20_N_2_O_2_ (320.39) C 74.98; H 6.29. Found C 75.12; H 6.16. ^1^H NMR (CDCl_3_, 500 MHz): *δ* 1.80 (m, 4H, 6-H_2_ and 7-H_2_), 2.76 (d-like m, 4H, 5-H_2_ and 8-H_2_), 4.87 (s, 2H, CH_2_-OH), 6.79 (s, 1H, 1-H), 7.31 (t-like m, 1H, 4″-H), 7.37 (s, 1H, 4-H), 7.46 (t-like m, 2H, 3″-H and 5″-H), 7.64 (d, 2H, *J* = 7.9 Hz, 2″-H and 6″-H), 8.00 (s, 1H, 5′-H); ^13^C NMR (CDCl_3_, 125 MHz): δ 23.3 and 23.6: C-6 and C-7, 29.0 and 29.5: C-5 and C-8, 56.8 (CH_2_-OH), 114.6 (C-3), 116.9 (C-1), 118.7 (2C, C-2″ and C-6″), 121.1 (C-4′), 126.9 (C-4), 127.8 (C-5′), 128.1 (C-4″), 128.2 (C-4a), 129.7 (2C, C-3″ and C-5″), 139.1 (C-1″), 139.2 (C-8a), 150.1 (C-3′), 153.7 (C-2); ESI-MS 321 [M + H]^+^.

##### 3-(4′-(Hydroxymethyl)-1′-(4″-tolyl)-1′H-pyrazol-3-yl)-5,6,7,8-tetrahydro-2-naphthol (**18b**)

According to the general procedure, compound **17b** (166 mg) was used. After purification with EtOAc/CH_2_Cl_2_ = 2:98 as eluent, **18b** was obtained as a white solid (139 mg, 83%). Mp 163–165 °C; Anal. Calcd. for C_21_H_22_N_2_O_2_ (334.42) C 75.42; H 6.63. Found C 75.55; H 6.51. ^1^H NMR (CDCl_3_, 500 MHz): *δ* 1.80 (m, 4H, 6-H_2_ and 7-H_2_), 2.39 (s, 3H, 4″-CH_3_), 2.76 (d-like m, 4H, 5-H_2_ and 8-H_2_), 4.86 (s, 2H, CH_2_-OH), 6.79 (s, 1H, 1-H), 7.26 (d, 2H, *J* = 8.5 Hz, 3″-H and 5″-H), 7.37 (s, 1H, 4-H), 7.52 (d, 2H, *J* = 8.5 Hz, 2″-H and 6″-H), 7.96 (s, 1H, 5′-H); ^13^C NMR (CDCl_3_, 125 MHz): *δ* 21.1 (4″-CH_3_), 23.3 and 23.7: C-6 and C-7, 29.0 and 29.6: C-5 and C-8, 56.8 (CH_2_-OH), 114.8 (C-3), 116.9 (C-1), 118.8 (2C, C-2″ and C-6″), 120.8 (C-4′), 127.8 (C-4), 128.1 (C-5′), 128.2 (C-4a), 130.2 (2C, C-3″ and C-5″), 136.8 (C-4″), 137.0 (C-1″), 139.1 (C-8a), 149.9 (C-3′), 153.8 (C-2); ESI-MS 335 [M + H]^+^. 

##### 3-(1′-(4″-Chlorophenyl)-4′-(hydroxymethyl)-1′H-pyrazol-3′-yl)-5,6,7,8-tetrahydro-2-naphthol (**18c**)

According to the general procedure, compound **17c** (176 mg) was used. After purification with EtOAc/CH_2_Cl_2_ = 2:98 as eluent, **18c** was obtained as a white solid (117 mg, 66%). Mp 154–156 °C; Anal. Calcd. for C_20_H_19_ClN_2_O_2_ (354.83) C 67.70; H 5.40. Found C 67.82; H 5.27. ^1^H NMR (CDCl_3_, 500 MHz): *δ* 1.80 (m, 4H, 6-H_2_ and 7-H_2_), 2.76 (d-like m, 4H, 5-H_2_ and 8-H_2_), 4.86 (s, 2H, CH_2_-OH), 6.79 (s, 1H, 1-H), 7.34 (s, 1H, 4-H), 7.43 (d, 2H, *J* = 9.0 Hz, 3″-H and 5″-H), 7.57 (d, 2H, *J* = 9.0 Hz, 2″-H and 6″-H), 7.97 (s, 1H, 5′-H); ^13^C NMR (CDCl_3_, 125 MHz): δ 23.3 and 23.7: C-6 and C-7, 29.0 and 29.6: C-5 and C-8, 56.8 (CH_2_-OH), 114.5 (C-3), 117.0 (C-1), 119.9 (2C, C-2″ and C-6″), 121.6 (C-4′), 127.7 (C-4), 128.2 (C-5′), 128.4 (C-4a), 129.9 (2C, C-3″ and C-5″), 132.4 (C-4″), 137.8 (C-1″), 139.5 (C-8a), 150.4 (C-3′), 153.8 (C-2); ESI-MS 355 [M + H]^+^.

#### 3.1.5. General Procedure for the Oxidative Lactonization of Steroidal and Non-steroidal 4-Formylpyrazoles (**7a**–**c** and **17a**–**c**)

4-formylpyrazole (**7a**–**c**, 0.30 mmol or **17a**–**c**, 0.50 mmol) was suspended in acetone (10 mL) and Jones reagent was added dropwise into the solution until its color remained. The mixture was then stirred at room temperature for 10 min (**7a**–**c**) or kept at reflux temperature for 30 min (**17a**–**c**). After the given reaction time, the solution was diluted with water (15 mL). The precipitate that formed was extracted with EtOAc (3 × 10 mL), and the combined organic phases were washed with brine (10 mL), then dried over anhydrous Na_2_SO_4_ and concentrated in vacuo. The crude product (**9a**–**c** or **19a**–**c**) was purified by column chromatography with EtOAc/hexane = 20:80.

##### 11β-Acetoxy-2-phenylpyrazolo [3,4:4,5]pyrano [2,3:3,2]estra-1,3,5(10)-triene-4(2H)-one (**9a**)

According to the general procedure, compound **7a** (145 mg) was used. Compound **9a** was obtained as a white solid (106 mg, 73%). Mp 198–200 °C; Anal. Calcd. for C_30_H_30_N_2_O_4_ (482.58) C 74.67; H 6.27. Found C 74.80; H 6.18. ^1^H NMR (CDCl_3_, 500 MHz): *δ* 0.86 (s, 3H, 11a-CH_3_), 1.28–1.64 (overlapping m, 7H), 1.78 (m, 1H), 1.95 (m, 2H), 2.07 (s, 3H, 11-OAc-CH_3_), 2.19–2.35 (overlapping m, 2H), 2.51 (m, 1H), 2.97 (m, 2H, 7-H_2_), 4.71 (t, 1H, *J* = 8.5 Hz, 11-H), 7.10 (s, 1H, 6-H), 7.44 (t-like m, 1H, 4′-H), 7.55 (t-like m, 2H, 3′-H and 5′-H), 7.84 (d, 2H, *J* = 8.0 Hz, 2′-H and 6′-H), 8.05 (s, 1H, 14-H), 8.65 (s, 1H, 3-H); ^13^C NMR (CDCl_3_, 125 MHz): *δ* 12.2 (11a-CH_3_), 21.4 (11-OAc-CH_3_), 23.4 (CH_2_), 26.4 (CH_2_), 27.1 (CH_2_), 27.7 (CH_2_), 29.9 (CH_2_), 36.9 (CH_2_), 38.3 (CH), 43.0 (C-11a), 44.1 (CH), 50.0 (CH), 82.8 (C-11), 109.4 (C-3a), 112.1 (C-14a), 117.3 (C-6), 119.6 (C-4′), 120.6 (2C, C-2′ and C-6′), 128.6 (C-14), 129.6 (C-3), 130.0 (2C, C-3′ and C-5′), 137.5 (C-13b), 139.4 (C-1′), 140.7 (C-6a), 150.4 and 151.3 (C-5a, C-14b), 158.5 (C-4), 171.4 (11a-OAc-CO); ESI-MS 483 [M + H]^+^.

##### 11β-Acetoxy-2-(4′-tolyl)-pyrazolo[3,4:4,5]pyrano[2,3:3,2]estra-1,3,5(10)-triene-4(2H)-one (**9b**)

According to the general procedure, compound **7b** (150 mg) was used. Compound **9b** was obtained as a white solid (103 mg, 69%). Mp 264–266 °C; Anal. Calcd. for C_31_H_32_N_2_O_4_ (496.61) C 74.98; H 6.50. Found C 75.12; H 6.38. ^1^H NMR (CDCl_3_, 500 MHz): *δ* 0.86 (s, 3H, 11a-CH_3_), 1.28–1.65 (overlapping m, 7H), 1.77 (m, 1H), 1.95 (m, 2H), 2.07 (s, 3H, 11-OAc-CH_3_), 2.20–2.33 (overlapping m, 2H), 2.43 (s, 3H, 4′-CH_3_), 2.50 (m, 1H), 2.97 (m, 2H, 7-H_2_), 4.71 (t, 1H, *J* = 8.5 Hz, 11-H), 7.09 (s, 1H, 6-H), 7.34 (d, 2H, *J* = 8.4 Hz, 3′-H and 5′-H), 7.71 (d, 2H, *J* = 8.4 Hz, 2′-H and 6′-H), 8.04 (s, 1H, 14-H), 8.60 (s, 1H, 3-H); ^13^C NMR (CDCl_3_, 125 MHz): 12.2 (11a-CH_3_), 21.2 (4′-CH_3_), 21.4 (11-OAc-CH_3_), 23.4 (CH_2_), 26.4 (CH_2_), 27.1 (CH_2_), 27.7 (CH_2_), 29.9 (CH_2_), 36.9 (CH_2_), 38.3 (CH), 43.0 (C-11a), 44.1 (CH), 50.0 (CH), 82.8 (C-11), 109.2 (C-3a), 112.1 (C-14a), 117.2 (C-6), 119.6 (C-14), 120.4 (2C, C-2′ and C-6′), 129.4 (C-3), 130.4 (2C, C-3′ and C-5′), 137.1 (C-4′), 137.4 (C-13b), 138.8 (C-1′), 140.5 (C-6a), 150.3 and 151.3 (C-5a, C-14b), 158.5 (C-4), 171.4 (11a-OAc-CO); ESI-MS 497 [M + H]^+^.

##### 11β-Acetoxy-2-(4′-chlorophenyl)-pyrazolo[3,4:4,5]pyrano[2,3-3,2]estra-1,3,5(10)-triene-4(2H)-one (**9c**)

According to the general procedure, compound **7c** (156 mg) was used. Compound **9c** was obtained as a white solid (112 mg, 72%). Mp 283–285 °C; Anal. Calcd. for C_30_H_29_ClN_2_O_4_ (517.02) C 69.69; H 5.65. Found C 69.81; H 5.52. ^1^H NMR (CDCl_3_, 500 MHz): *δ* 0.86 (s, 3H, 11a-CH_3_), 1.27–1.65 (overlapping m, 7H), 1.78 (m, 1H), 1.95 (m, 2H), 2.06 (s, 3H, 11-OAc-CH_3_), 2.21–2.34 (overlapping m, 2H), 2.49 (m, 1H), 2.97 (m, 2H, 7-H_2_), 4.71 (t, 1H, *J* = 8.5 Hz, 11-H), 7.09 (s, 1H, 6-H), 7.52 (d, 2H, *J* = 8.8 Hz, 3′-H and 5′-H), 7.79 (d, 2H, *J* = 8.8 Hz, 2′-H and 6′-H), 8.03 (s, 1H, 14-H), 8.62 (s, 1H, 3-H); ^13^C NMR (CDCl_3_, 125 MHz): δ 12.2 (11a-CH_3_), 21.4 (11-OAc-CH_3_), 23.4 (CH_2_), 26.4 (CH_2_), 27.0 (CH_2_), 27.7 (CH_2_), 29.9 (CH_2_), 36.9 (CH_2_), 38.3 (CH), 43.0 (C-11a), 44.1 (CH), 50.0 (CH), 82.7 (C-11), 109.4 (C-3a), 111.8 (C-14a), 117.3 (C-6), 119.6 (C-14), 121.6 (2C, C-2′ and C-6′), 129.5 (C-3), 130.1 (2C, C-3′ and C-5′), 134.3 (C-4′), 137.6 (C-13b), 137.9 (C-1′), 140.9 (C-6a), 150.6 and 151.3 (C-5a, C-14b), 158.2 (C-4), 171.4 (11a-OAc-CO); ESI-MS 518 [M + H]^+^.

##### 2-Phenyl-7,8,9,10-tetrahydrobenzo[6,7]chromeno[4,3-c]pyrazol-4(2H)-one (**19a**)

According to the general procedure, compound **17a** (159 mg) was used. Compound **19a** was obtained as a white solid (101 mg, 64%). Mp 221–223 °C; Anal. Calcd. for C_20_H_16_N_2_O_2_ (316.36) C 75.93; H 5.10. Found C 76.09; H 5.19. ^1^H NMR (CDCl_3_, 500 MHz): δ 1.84 (m, 4H, 8-H_2_ and 9-H_2_), 2.87 (d-like m, 4H, 7-H_2_ and 10-H_2_), 7.09 (s, 1H, 6-H), 7.44 (t-like m, 1H, 4′-H), 7.55 (t-like m, 2H, 3′-H and 5′-H), 7.83 (d, 2H, *J* = 7.9 Hz, 2′-H and 6′-H), 7.85 (s, 1H, 11-H), 8.64 (s, 1H, 3-H); ^13^C NMR (CDCl_3_, 125 MHz): δ 22.9 and 23.2: C-8 and C-9, 29.0 and 29.9: C-7 and C-10, 109.5 (C-3a), 112.0 (C-11a), 117.4 (C-6), 117.4 (2C, C-2′ and C-6′), 122.8 (C-4′), 128.6 (C-11), 129.6 (C-3), 129.9 (2C, C-3′ and C-5′), 134.1 (C-10a), 139.4 (C-1′), 140.9 (C-6a), 150.3 (C-5a), 151.2 (C-11b), 158.5 (C-4); ESI-MS 317 [M + H]^+^.

##### 2-(4′-Tolyl)-7,8,9,10-tetrahydrobenzo[6,7]chromeno[4,3-c]pyrazol-4(2H)-one (**19b**)

According to the general procedure, compound **17b** (166 mg) was used. Compound **19b** was obtained as a white solid (89 mg, 54%). Mp 224–226 °C; Anal. Calcd. for C_21_H_18_N_2_O_2_ (330.39) C 76.34; H 5.49. Found C 76.48; H 5.36. ^1^H NMR (CDCl_3_, 500 MHz): δ1.84 (m, 4H, 8-H_2_ and 9-H_2_), 2.43 (s, 3H, 4′-CH_3_), 2.86 (d-like m, 4H, 7-H_2_ and 10-H_2_), 7.08 (s, 1H, 6-H), 7.33 (d, 2H, *J* = 8.5 Hz, 3′-H and 5′-H), 7.69 (d, 2H, *J* = 8.5 Hz, 2′-H and 6′-H), 7.84 (s, 1H, 11-H), 8.59 (s, 1H, 3-H); ^13^C NMR (CDCl_3_, 125 MHz): δ21.2 (4′-CH_3_), 23.0 and 23.2: C-8 and C-9, 29.0 and 29.9: C-7 and C-10, 109.3 (C-3a), 112.2 (C-11a), 117.3 (C-6), 120.4 (2C, C-2′ and C-6′), 122.8 (C-11), 129.4 (C-3), 130.4 (2C, C-3′ and C-5′), 134.0 (C-10a), 137.2 (C-4′), 138.7 (C-1′), 140.8 (C-6a), 150.2 (C-5a), 151.2 (C-11b), 158.5 (C-4); ESI-MS 331 [M + H]^+^.

##### 2-(4′-Chlorophenyl)-7,8,9,10-tetrahydrobenzo[6,7]chromeno[4,3-c]pyrazol-4(2H)-one (**19c**)

According to the general procedure, compound 17c (176 mg) was used. Compound 19c was obtained as a white solid (89 mg, 51%). Mp 291–293 °C; Anal. Calcd. for C_20_H_15_ClN_2_O_2_ (350.80) C 68.48; H 4.31. Found C 68.60; H 4.22. ^1^H NMR (CDCl_3_, 500 MHz): δ1.85 (m, 4H, 8-H_2_ and 9-H_2_), 2.87 (d-like m, 4H, 7-H_2_ and 10-H_2_), 7.09 (s, 1H, 6-H), 7.52 (d, 2H, *J* = 9.0 Hz, 3′-H and 5′-H), 7.79 (d, 2H, *J* = 9.0 Hz, 2′-H and 6′-H), 7.83 (s, 1H, 11-H), 8.61 (s, 1H, 3-H); ^13^C NMR (CDCl_3_, 125 MHz): δ 22.9 and 23.2: C-8 and C-9, 29.0 and 30.0: C-7 and C-10, 109.9 (C-3a), 111.9 (C-11a), 117.4 (C-6), 121.6 (2C, C-2′ and C-6′), 122.9 (C-11), 129.5 (C-3), 130.1 (2C, C-3′ and C-5′), 134.2 (C-10a), 134.4 (C-4′), 138.0 (C-1′), 141.2 (C-6a), 150.5 (C-5a), 151.3 (C-11b), 158.3 (C-4); ESI-MS 351 [M + H]^+^.

#### 3.1.6. General Procedure for the Deacetylation of Compounds **4**, **7a**–**c** and **10a**–**c**

Compound **4** (0.50 mmol), **7a**–**c** or **10a**–**c** (0.30 mmol) was dissolved in MeOH (10 mL), and KOH (50 mg, 0.89 mmol) was added. The solution was stirred at room temperature for 2 h (for **4** and **7a**–**c**) or 40 °C for 3 h (for **10a**–**c**), then poured into water and neutralized with diluted HCl. The resulting precipitate was filtered off, washed with water, and dried.

##### 2-Acetyl-estra-1,3,5(10)-triene-3,17β-diol (**5**)

According to the general procedure, compound **4** (178 mg) was used. Yield (**5**): 141 mg (90%, white solid); Mp 189–191 °C (192–195 °C [38]); Anal. Calcd. for C_20_H_26_O_3_ (314.43) C 76.40; H 8.34. Found C 76.56; H 8.25. ^1^H NMR (CDCl_3_, 500 MHz): *δ* 0.79 (s, 3H, 18-CH_3_), 1.17–1.59 (overlapping m, 7H), 1.71 (m, 1H), 1.89 (m, 1H), 1.99 (m, 1H), 2.10–2.19 (overlapping m, 2H), 2.34 (m, 1H), 2.60 (s, 3H, Ac-CH_3_), 2.85 (m, 2H, 6-H_2_), 3.74 (t, 1H, *J* = 8.6 Hz, 17-H), 6.69 (s, 1H, 4-H), 7.61 (s, 1H, 1-H), 12.04 (s, 1H, OH); ^13^C NMR (CDCl_3_, 125 MHz): δ 11.2 (C-18), 23.2 (CH_2_), 26.4 (CH_2_), 26.6 (Ac-CH_3_), 26.9 (CH_2_), 30.1 (CH_2_), 30.7 (CH_2_), 36.7 (CH_2_), 38.7 (CH), 43.3 (C-13), 43.6 (CH), 50.2 (CH), 81.9 (C-17), 117.7 (C-4), 118.0 (C-2), 127.4 (C-1), 131.7 (C-10), 147.4 (C-5), 160.1 (C-3), 204.2 (Ac-CO); ESI-MS 315 [M + H]^+^.

##### 2-(4′-Formyl-1′-phenyl-1′H-pyrazol-3′-yl)-estra-1,3,5(10)-trien-3,17β-diol (**8a**)

According to the general procedure, compound **7a** (145 mg) was used. Yield (**8a**): 118 mg (89%, white solid); Mp 172–174 °C; Anal. Calcd. for C_28_H_30_N_2_O_3_ (442.56) C 75.99; H 6.83. Found C 76.08; H 6.74. ^1^H NMR (CDCl_3_, 500 MHz): *δ* 0.81 (s, 3H, 18-CH_3_), 1.30–1.43 (overlapping m, 4H), 1.45–1.63 (overlapping m, 3H), 1.72 (m, 1H), 1.92 (m, 1H), 1.99 (m, 1H), 2.13 (m, 1H), 2.25 (m, 1H), 2.43 (m, 1H), 2.90 (m, 2H, 6-H_2_), 3.76 (t, 1H, *J* = 8.5 Hz, 17-H), 6.83 (s, 1H, 4-H), 7.43 (t-like m, 1H, 4″-H), 7.55 (t-like m, 2H, 3″-H and 5″-H), 7.73 (d, 2H, *J* = 7.9 Hz, 2″-H and 6″-H), 7.90 (s, 1H, 1-H), 8.57 (s, 1H, 5′-H), 10.20 (s, 1H, CHO); ^13^C NMR (CDCl_3_, 125 MHz): δ 11.1 (C-18), 23.2 (CH_2_), 26.5 (CH_2_), 27.2 (CH_2_), 29.6 (CH_2_), 30.6 (CH_2_), 36.7 (CH_2_), 38.8 (CH), 43.3 (C-13), 43.9 (CH), 50.1 (CH), 81.9 (C-17), 113.0 (C-4′), 117.0 (C-4), 119.6 (2C, C-2″ and C-6″), 123.0 (C-2), 126.8 (C-1), 128.4 (C-4″), 129.9 (2C, C-3″ and C-5″), 132.2 (C-10), 132.7 (C-5′), 138.2 (C-1″), 140.7 (C-5), 153.1 (C-3′), 153.7 (C-3), 184.4 (CHO); ESI-MS 443 [M + H]^+^.

##### 2-(4′-Formyl-1′-(4″-tolyl)-1′H-pyrazol-3′-yl)-estra-1,3,5(10)-trien-3,17β-diol (**8b**)

According to the general procedure, compound **7b** (150 mg) was used. Yield (**8b**): 123 mg (91%, white solid); Mp > 190 °C (decomp.); Anal. Calcd. for C_29_H_32_N_2_O_3_ (456.59) C 76.29; H 7.06. Found C 76.43; H 6.95. ^1^H NMR (CDCl_3_, 500 MHz): *δ* 0.80 (s, 3H, 18-CH_3_), 1.30–1.43 (overlapping m, 4H), 1.44–1.62 (overlapping m, 3H), 1.71 (m, 1H), 1.91 (m, 1H), 1.99 (m, 1H), 2.13 (m, 1H), 2.25 (m, 1H), 2.42 (m, 1H), 2.43 (s, 3H, 4″-CH_3_), 2.90 (m, 2H, 6-H_2_), 3.75 (t, 1H, *J* = 8.5 Hz, 17-H), 6.83 (s, 1H, 4-H), 7.32 (d, 2H, *J* = 8.5 Hz, 3″-H and 5″-H), 7.59 (d, 2H, *J* = 8.5 Hz, 2″-H and 6″-H), 7.90 (s, 1H, 1-H), 8.52 (s, 1H, 5′-H), 9.90 (bs, 1H, 3-OH), 10.20 (s, 1H, CHO); ^13^C NMR (CDCl_3_, 125 MHz): *δ* 11.2 (C-18), 21.2 (4″-CH_3_), 23.3 (CH_2_), 26.6 (CH_2_), 27.3 (CH_2_), 29.7 (CH_2_), 30.7 (CH_2_), 36.8 (CH_2_), 38.9 (CH), 43.4 (C-13), 44.0 (CH), 50.2 (CH), 82.0 (C-17), 113.2 (C-4′), 117.1 (C-4), 119.6 (2C, C-2″ and C-6″), 122.9 (C-2), 126.9 (C-1), 130.5 (2C, C-3″ and C-5″), 132.2 (C-10), 132.6 (C-5′), 136.1 (C-4″), 138.7 (C-1″), 140.7 (C-5), 153.1 (C-3′), 153.8 (C-3), 184.6 (CHO); ESI-MS 457 [M + H]^+^.

##### 2-(1′-(4″-Chlorophenyl)-1′H-pyrazol-3′-yl)-estra-1,3,5(10)-trien-3,17β-diol (**8c**)

According to the general procedure, compound **7c** (156 mg) was used. Yield (**8c**): 124 mg (87%, white solid); Mp > 190 °C (decomp.); Anal. Calcd. for C_28_H_29_ClN_2_O_3_ (477.00) C 70.50; H 6.13. Found C 70.62; H 6.04. ^1^H NMR (CDCl_3_, 500 MHz): *δ* 0.80 (s, 3H, 18-CH_3_), 1.30–1.43 (overlapping m, 4H), 1.44–1.62 (overlapping m, 3H), 1.72 (m, 1H), 1.91 (m, 1H), 1.99 (m, 1H), 2.13 (m, 1H), 2.24 (m, 1H), 2.41 (m, 1H), 2.90 (m, 2H, 6-H_2_), 3.75 (t, 1H, *J* = 8.5 Hz, 17-H), 6.83 (s, 1H, 4-H), 7.50 (d, 2H, *J* = 8.9 Hz, 3″-H and 5″-H), 7.66 (d, 2H, *J* = 8.9 Hz, 2″-H and 6″-H), 7.85 (s, 1H, 1-H), 8.53 (s, 1H, 5′-H), 9.66 (bs, 1H, 3-OH), 10.18 (s, 1H, CHO); ^13^C NMR (CDCl_3_, 125 MHz): δ 11.2 (C-18), 23.3 (CH_2_), 26.6 (CH_2_), 27.3 (CH_2_), 29.7 (CH_2_), 30.7 (CH_2_), 36.8 (CH_2_), 38.9 (CH), 43.4 (C-13), 44.0 (CH), 50.2 (CH), 82.0 (C-17), 113.0 (C-4′), 117.2 (C-4), 120.8 (2C, C-2″ and C-6″), 123.3 (C-2), 126.9 (C-1), 130.2 (2C, C-3″ and C-5″), 132.4 (C-10), 132.7 (C-5′), 134.2 (C-4″), 136.9 (C-1″), 141.0 (C-5), 153.5 (C-3′), 153.7 (C-3), 184.5 (CHO); ESI-MS 478 [M + H]^+^.

##### 3-(4′-(Hydroxymethyl)-1′-phenyl-1′H-pyrazol-3′-yl)-estra-1,3,5(10)-trien-3,17β-diol (**11a**)

According to the general procedure, compound **10a** (146 mg) was used. Yield (**11a**): 121 mg (91%, white solid); Mp 141–143 °C; Anal. Calcd. for C_28_H_32_N_2_O_3_ (444.58) C 75.65; H 7.26. Found C 75.74; H 7.13. ^1^H NMR (DMSO-*d*_6_, 500 MHz): *δ* 0.68 (s, 3H, 18-CH_3_), 1.10–1.44 (overlapping m, 7H), 1.59 (m, 1H), 1.80–1.90 (overlapping m, 3H), 2.14 (m, 1H), 2.33 (m, 1H), 2.77 (m, 2H, 6-H_2_), 3.54 (m, 1H, 17-H), 4.51 (d, 1H, *J* = 4.8 Hz, 17-OH), 4.56 (d, 2H, *J* = 4.9 Hz, CH_2_-OH), 5.15 (t, 1H, *J* = 4.9 Hz, CH_2_-OH), 6.65 (s, 1H, 4-H), 7.31 (t-like m, 1H, 4″-H), 7.53 (t-like m, 2H, 3″-H and 5″-H), 7.65 (s, 1H, 1-H), 7.83 (d, 2H, *J* = 8.0 Hz, 2″-H and 6″-H), 8.60 (s, 1H, 5′-H), 10.14 (3-OH); ^13^C NMR (DMSO-*d*_6_, 125 MHz): *δ* 11.3 (C-18), 22.8 (CH_2_), 26.0 (CH_2_), 26.9 (CH_2_), 28.9 (CH_2_), 29.9 (CH_2_), 36.6 (CH_2_), 38.6 (CH), 42.8 (C-13), 43.6 (CH), 49.6 (CH), 54.7 (CH_2_-OH), 80.1 (C-17), 115.2 (C-2), 115.8 (C-4), 118.0 (2C, C-2″ and C-6″), 122.6 (C-4′), 126.2 (2C, C-4″ and C-1), 128.4 (C-5′), 129.7 (2C, C-3″ and C-5″), 131.1 (C-10), 137.9 (C-5), 139.0 (C-1″), 149.8 (C-3′), 152.9 (C-3); ESI-MS 445 [M + H]^+^.

##### 3-(4′-(Hydroxymethyl)-1′-(4″-tolyl)-1′H-pyrazol-3′-yl)-estra-1,3,5(10)-trien-3,17β-diol (**11b**)

According to the general procedure, compound **10b** (150 mg) was used. Yield (**11b**): 122 mg (89%, white solid); Mp 201–203 °C; Anal. Calcd. for C_29_H_34_N_2_O_3_ (458.60) C 75.95; H 7.47. Found C 76.07; H 7.39. ^1^H NMR (DMSO-*d*_6_, 500 MHz): *δ* 0.69 (s, 3H, 18-CH_3_), 1.11–1.43 (overlapping m, 7H), 1.60 (m, 1H), 1.81–1.94 (overlapping m, 3H), 2.16 (m, 1H), 2.34 (m, 1H), 2.36 (s, 3H, 4″-CH_3_), 2.78 (m, 2H, 6-H_2_), 3.55 (m, 1H, 17-H), 4.52 (d, 1H, *J* = 4.9 Hz, 17-OH), 4.56 (d, 2H, *J* = 4.9 Hz, CH_2_OH), 5.18 (t, 1H, *J* = 4.9 Hz, CH_2_OH), 6.65 (s, 1H, 4-H), 7.34 (d, 2H, *J* = 8.5 Hz, 3″-H and 5″-H), 7.67 (s, 1H, 1-H), 7.72 (d, 2H, *J* = 8.5 Hz, 2″-H and 6″-H), 7.96 (s, 1H, 5′-H), 9.95 (3-OH); ^13^C NMR (DMSO-*d*_6_, 125 MHz): δ 11.3 (C-18), 20.4 (4″-CH_3_), 22.8 (CH_2_), 26.0 (CH_2_), 26.9 (CH_2_), 28.9 (CH_2_), 29.9 (CH_2_), 36.6 (CH_2_), 38.6 (CH), 42.8 (C-13), 43.6 (CH), 49.6 (CH), 54.7 (CH_2_-OH), 80.1 (C-17), 115.2 (C-2), 115.8 (C-4), 117.9 (2C, C-2″ and C-6″), 122.2 (C-4′), 126.1 (C-1), 128.3 (C-5′), 130.1 (2C, C-3″ and C-5″), 131.0 (C-4″), 135.6 (C-10), 136.8 (C-1″), 137.8 (C-5), 149.5 (C-3′), 152.9 (C-3); ESI-MS 459 [M + H]^+^.

##### 3-(1′-(4″-Chlorophenyl)-4′-(hydroxymethyl)-1′H-pyrazol-3′-yl)-estra-1,3,5(10)-trien-3,17β-diol (**11c**)

According to the general procedure, compound **10c** (156 mg) was used. Yield (**11c**): 126 mg (88%, white solid); Mp 240–242 °C; Anal. Calcd. for C_28_H_31_ClN_2_O_3_ (479.02) C 70.21; H 6.52. Found C 70.09; H 6.43. ^1^H NMR (DMSO-*d*_6_, 500 MHz): *δ* 0.68 (s, 3H, 18-CH_3_), 1.10–1.44 (overlapping m, 7H), 1.59 (m, 1H), 1.79–1.93 (overlapping m, 3H), 2.14 (m, 1H), 2.32 (m, 1H), 2.77 (m, 2H, 6-H_2_), 3.54 (m, 1H, 17-H), 4.50 (d, 1H, *J* = 4.9 Hz, 17-OH), 4.53 (d, 2H, *J* = 4.9 Hz, CH_2_OH), 5.18 (t, 1H, *J* = 4.9 Hz, CH_2_OH), 6.64 (s, 1H, 4-H), 7.57 (d, 2H, *J* = 9.0 Hz, 3″-H and 5″-H), 7.59 (s, 1H, 1-H), 7.87 (d, 2H, *J* = 8.9 Hz, 2″-H and 6″-H), 8.55 (s, 1H, 5′-H), 9.95 (3-OH); ^13^C NMR (DMSO-*d*_6_, 125 MHz): δ 11.3 (C-18), 22.8 (CH_2_), 26.0 (CH_2_), 26.9 (CH_2_), 28.9 (CH_2_), 29.9 (CH_2_), 36.6 (CH_2_), 38.6 (CH), 42.8 (C-13), 43.6 (CH), 49.6 (CH), 54.7 (CH_2_-OH), 80.1 (C-17), 115.3 (C-2), 115.8 (C-4), 119.6 (2C, C-2″ and C-6″), 123.1 (C-4′), 126.4 (C-1), 128.4 (C-5′), 129.6 (2C, C-3″ and C-5″), 130.2 and 131.1: C-4″ and C-10, 137.9 (C) and 138.0 (C): C-5 and C-1″, 150.0 (C-3′), 152.9 (C-3); ESI-MS 479 [M + H]^+^.

### 3.2. Pharmacology

To evaluate the in vitro pharmacological effects of the synthesized molecules, each compound was first dissolved in cell culture grade DMSO (Sigma) to a final concentration of 10 mM. Using MTT colorimetric assay [36], the cytotoxicity of these compounds was evaluated on cancerous (MCF-7, HeLa, DU145, PC-3) and non-cancerous (MRC-5) cell lines. Briefly, 5000 cells/well of each cell line were seeded in 96-well plates and were left to grow overnight in a 5% CO_2_ atmosphere at 37 °C. On the next day, cells were treated with each derivative separately, in case of the primary screen, or only with the selected compounds upon IC_50_ determination. Cells were incubated with the test compounds for 72 h in a 5% CO_2_ atmosphere at 37 °C. End point was measured by incubating cells for 1 h with 0.5 mg/mL of MTT reagent (Serva) dissolved in serum free culture medium. The formazan crystals formed by metabolically active cells were later dissolved in DMSO. Optical density of the obtained solutions was measured at 570 nm using a Synergy HTX plate reader. For the overall screen, all compounds were used at 2.5 µM concentration; for the determination of IC_50_ values, the selected compounds were applied in 1, 2, 3, 4, 5, 6, and 7 µM concentrations, and the positive control cisplatin was applied for 24 h in 20, 40, 80, 160, and 330 µM concentrations. The analysis and conclusions were made based on three independent experiments. Normalized data were analyzed and the heat map was constructed using GraphPad Prism 7 software.

## 4. Conclusions

In summary, an AlCl_3_-induced one-pot process has been developed for the regioselective *ortho*-acetylation/demethylation of *O*-protected tetracyclic steroidal and bicyclic non-steroidal phenols under mild conditions. The acetylated phenols were then successfully subjected to MW-assisted arylhydrazone formation with three arylhydrazines having different *p*-substituted groups, which were then treated with the Vilsmeier-Haack reagent to give pyrazolyl estradiol and tetrahydronaphthol derivatives via cyclization and subsequent formylation. The resulting 4-formylpyrazoles were further converted by reduction to give 4-hydroxymethylpyrazoles, while pyrazolocoumarins were obtained by lactonization under oxidative conditions. Significant differences were observed during the similar reactions of the steroid and the smaller analogue modelling the A- and B-rings of estradiol, and a substituent effect mainly due to the presence of an electron-withdrawing Cl atom was also noticed in certain reaction steps. Among the synthesized molecules (steroid and non-steroid), we found 10–15 potent cancer cell-selective molecules, many of them proved to be competent against HeLa, DU145, or cisplatin-resistant PC-3 cells. Structure-activity considerations suggested that several simplified small molecules were able to induce significant toxicity; however, when the same structural motif was incorporated into an estrane framework, the resulting compounds were either more effective on the cancer cells or were able to target a different cell line. Moreover, acetylated steroidal pyrazoles proved to be less advantageous than their deacetylated counterparts due to reduced cancer cell selectivity. The unsubstituted phenyl ring on the pyrazole moiety proved to be the most favorable for selective antiproliferative effect.

## Figures and Tables

**Table 1:** Synthesis of arylhydrazones from steroidal and non-steroidal methyl ketones and their conversion to 4-formylpyrazoles.

Entry	Starting Material	R	Arylhydrazone	Yield ^1^ (%)	4-Formylpyrazole	Yield ^2^ (%)
1	**4**	Ph	**6a**	76	**7a**	75 ^3^
2	**4**	*p*-CH_3_-C_6_H_4_	**6b**	78	**7b**	78 ^3^
3	**4**	*p*-Cl-C_6_H_4_	**6c**	75	**7c**	72 ^3^
4	**15**	Ph	**16a**	- ^4^	**17a**	38 ^5^
5	**15**	*p*-CH_3_-C_6_H_4_	**16b**	- ^4^	**17b**	41 ^5^
6	**15**	*p*-Cl-C_6_H_4_	**16c**	- ^4^	**17c**	34 ^5^

^1^ Crude isolated products without purification; ^2^ After chromatographic purification; ^3^ Yields refer to hydrazones **6a**–**c**; ^4^ Could not be isolated; ^5^ Yields refer to **15**.

**Table 2:**
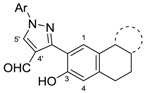
Selected ^1^H-NMR spectral parameters for steroidal (**7a**–**c**) and non-steroidal 4-formylpyrazoles (**17a**–**c**) in CDCl_3_ at 500 MHz.

Compound	Ar	*δ* (ppm)				
		4-H (s)^1^	1-H (s)^1^	5′-H (s)^1^	3-OH (bs)^2^	4′-CHO (s)^1^
**7a**	Ph	6.83	7.88	8.56	9.84	10.19
**7b**	*p*-CH_3_-C_6_H_4_	6.82	7.88	8.51	9.89	10.17
**7c**	*p*-Cl-C_6_H_4_	6.83	7.84	8.53	9.65	10.18
**17a**	Ph	6.83	7.53	8.56	9.68	10.20
**17b**	*p*-CH_3_-C_6_H_4_	6.82	7.55	8.50	9.69	10.19
**17c**	*p*-Cl-C_6_H_4_	6.82	7.51	8.52	9.45	10.18

^1^ Singlet; ^2^ Broad singlet.

**Table 3:** IC_50_ values (µM ± SD) of the selected compounds determined on various cancer cell lines and on non-cancerous MRC-5 cells.

	MRC-5	PC-3	DU145	MCF-7	HeLa
**7a**	5.90 ± 1.0	3.63 ± 1.0	6.93 ± 1.0	4.49 ± 1.0	4.18 ± 1.0
**7c**	4.76 ± 1.1	2.14 ± 1.1	3.37 ± 1.0	1.99 ± 1.1	5.32 ± 1.1
**8a**	5.12 ± 1.0	3.05 ± 1.0	6.46 ± 1.0	5.03 ± 1.0	4.09 ± 1.0
**8c**	5.53 ± 1.0	5.08 ± 1.0	4.82 ± 1.0	5.34 ± 1.0	3.39 ± 1.0
**9a**	6.41 ± 1.1	4.22 ± 1.0	5.66 ± 1.0	4.94 ± 1.0	1.55 ± 1.0
**11a**	>7.00 ± 1.0	2.90 ± 1.3	>7.00 ± 1.0	6.80 ± 1.0	4.30 ± 1.0
**15**	>7.00 ± 1.2	>7.00 ± 1.4	>7.00 ± 1.2	>7.00 ± 1.2	3.60 ± 1.2
**17a**	6.26 ± 1.1	4.30 ± 1.0	5.62 ± 1.0	5.17 ± 1.0	2.20 ± 1.1
**17c**	5.72 ± 1.1	4.39 ± 1.0	7.45 ± 1.0	4.12 ± 1.0	6.23 ± 1.0
**18b**	>7.00 ± 1.2	>7.00 ± 1.5	2.70 ± 1.1	>7.00 ± 1.0	>7.00 ± 1.1
**19a**	>7.00 ± 1.5	4.70 ± 1.0	1.41 ± 1.1	6.83 ± 1.1	>7.00 ± 1.1
**cisplatin**	34.3 ± 1.02	>330 ± 1.01	116.1 ± 1.06	>330 ± 1.03	297.1 ± 1.02

## Supplementary Materials

The following are available online, ^1^H NMR and ^13^C NMR spectra of the synthesized compounds, Table S1: Mean ± SD values of primary growth inhibitory screen (given as cell viability) used to construct the heat map, Figure S1: Dose–response curves used to evaluate IC_50_ concentrations of selected compounds.
