# Supplementary material for: Multistep Synthesis and In Vitro Anticancer Evaluation of 2-Pyrazolyl-Estradiol Derivatives, Pyrazolocoumarin-Estradiol Hybrids and Analogous Compounds
Source: Molecules. 2020 Sep 4;25(18):4039. doi: 10.3390/molecules25184039 (PMC7571145; doi:10.3390/molecules25184039)
Supplement: Supplementary file 1 [file molecules-25-04039-s001.pdf]

Supplementary material

for

Multistep Synthesis and *In Vitro* Anticancer Evaluation of 2-Pyrazolyl-Estradiol Derivatives, Pyrazolocoumarin-Estradiol Hybrids and Analogous Compounds

Barnabás Molnár [1], Mohana Krishna Gopisetty [2], Dóra Izabella Adamecz [2], Mónika Kiricsi [2] and Éva Frank [1,*]

[1] *Department of Organic Chemistry, Doctoral School of Chemistry, University of Szeged, Dóm tér 8, H-6720 Szeged, Hungary*

[2] *Department of Biochemistry and Molecular Biology, Doctoral School of Biology, University of Szeged, Közép fasor 52, H-6726, Szeged, Hungary*

*Corresponding author. E-mail address: frank@chem.u-szeged.hu

Table of Contents

^{1}H NMR and ^{13}C NMR spectra of the synthesized compounds S2-S28

Mean±SD values of primary growth inhibitory screen (given as cell viability) used to construct the heat map (Table S1) S29

Dose response curves used to evaluate IC50 concentrations of selected compounds. (Figure S1) S30

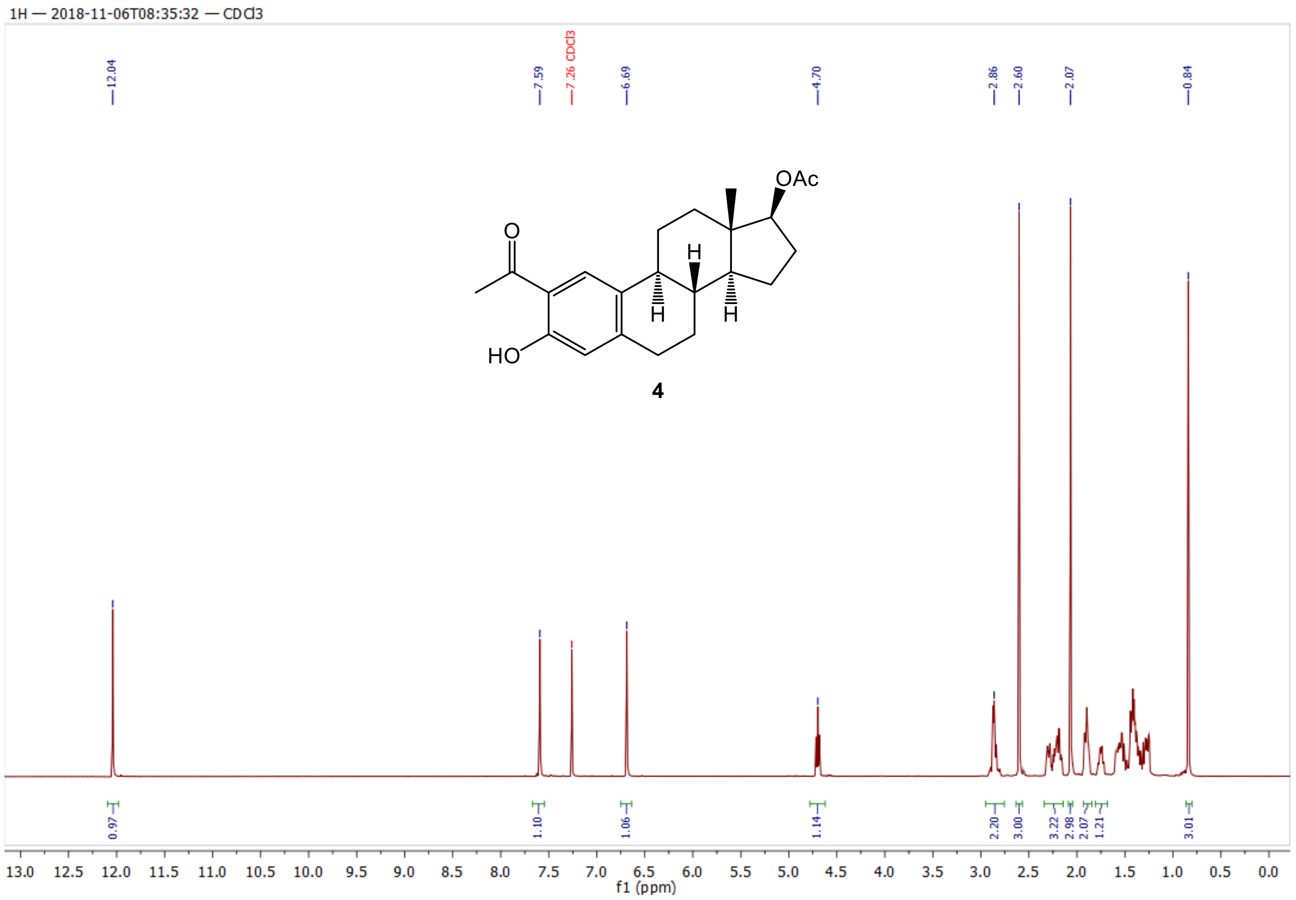
1H — 2018-11-06T08:35:32 — CDCl3
12.04
7.59
7.26 CDCl3
6.69
4.70
2.86
2.60
2.07
0.84
OAc
O
H
H
H
HO
4
0.97
1.10
1.06
1.14
2.20
3.00
3.22
2.98
2.07
1.21
3.01
f1 (ppm)

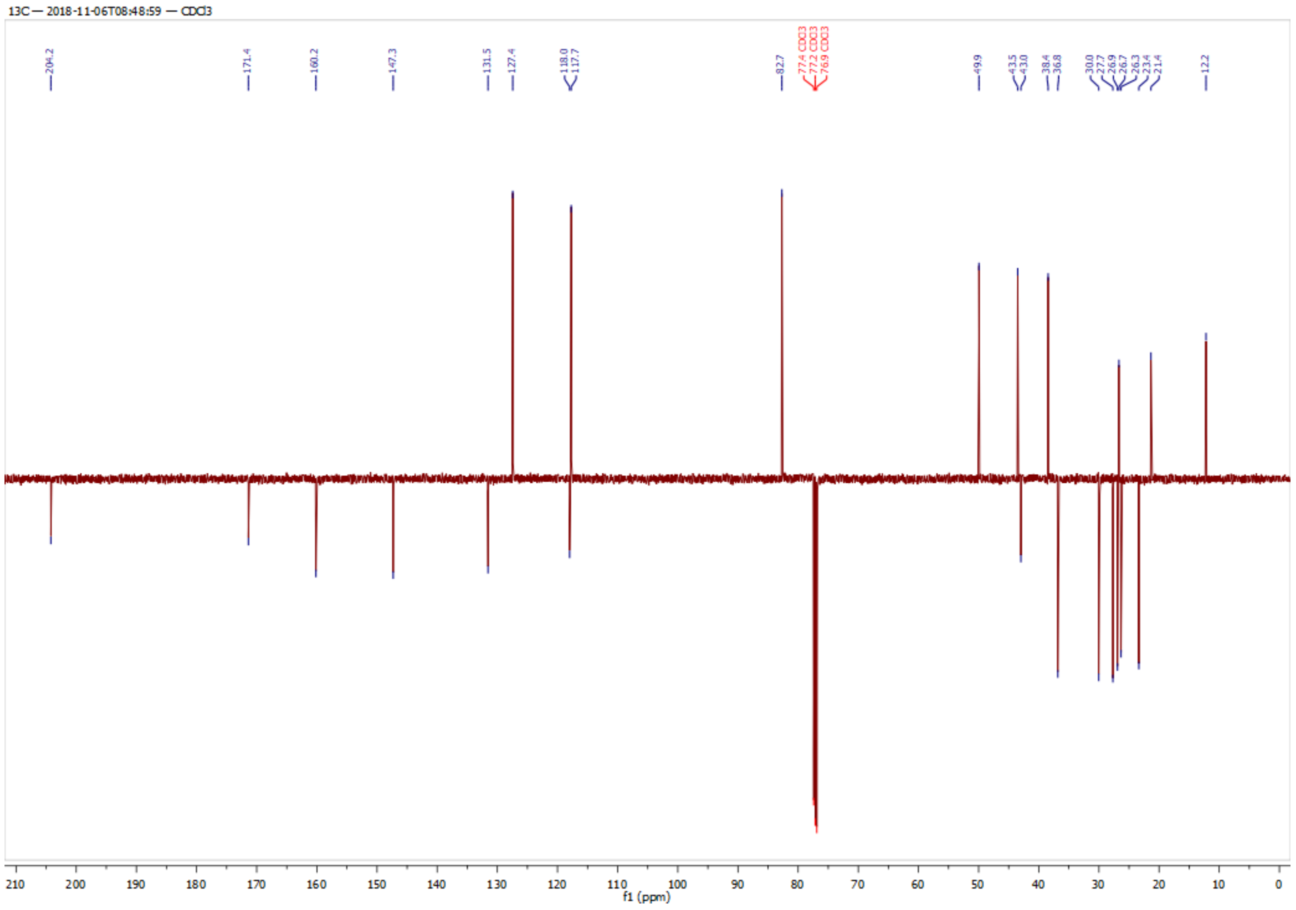
13C — 2018-11-06T08:48:59 — CDCl3
204.2
171.4
160.2
147.3
131.5
127.4
118.0
117.7
82.7
77.4 CDCl3
77.2 CDCl3
76.9 CDCl3
49.9
43.5
43.0
38.4
36.8
30.0
27.7
26.9
26.7
26.3
23.4
21.4
12.2
f1 (ppm)

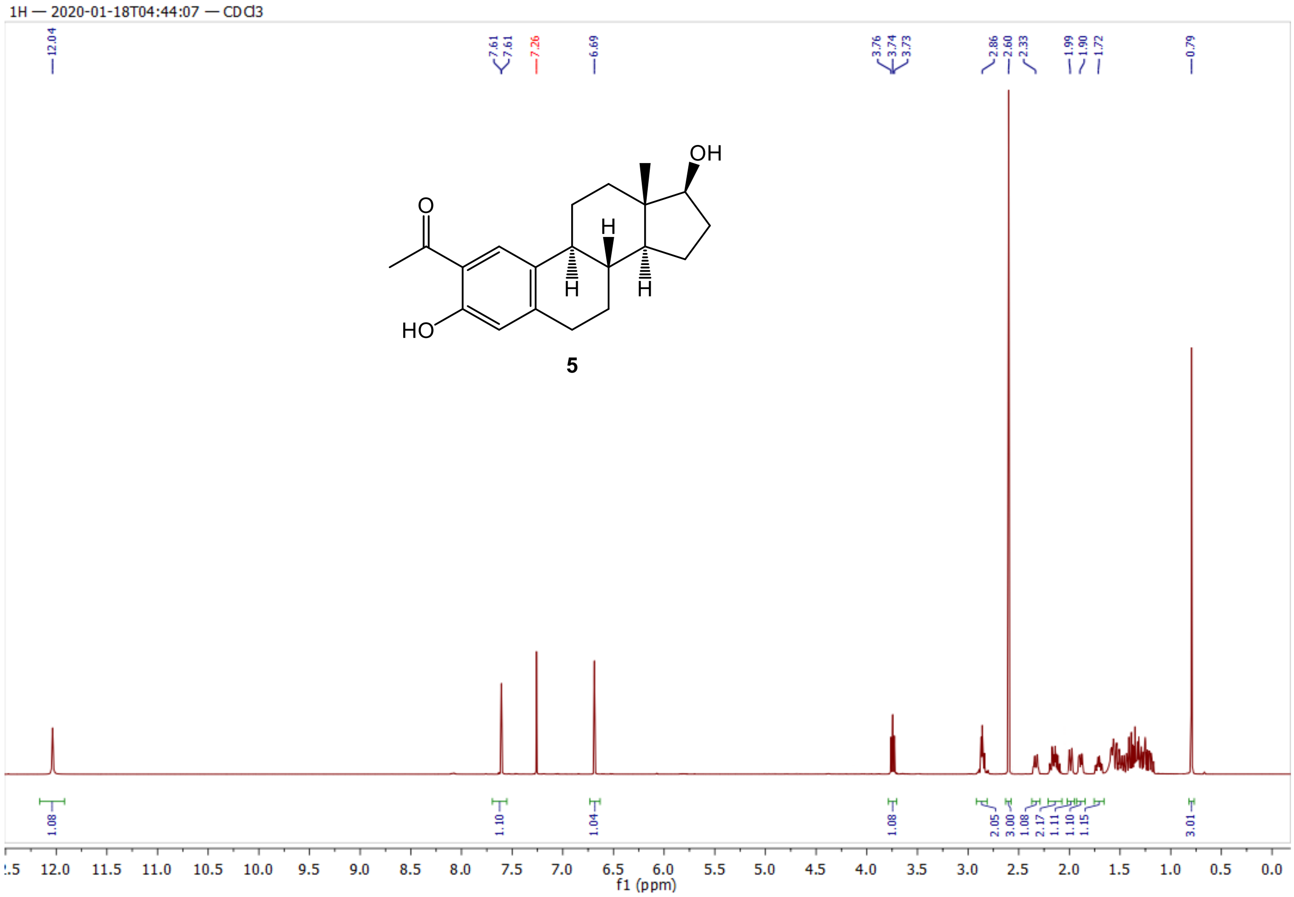
1H — 2020-01-18T04:44:07 — CDCl3
12.04
7.61
7.61
7.26
6.69
3.76
3.74
3.73
2.86
2.60
2.33
1.99
1.90
1.72
0.79
OH
O
H
H
H
HO
5
1.08
1.10
1.04
1.08
2.05
3.00
1.08
2.17
1.11
1.10
1.15
3.01
f1 (ppm)

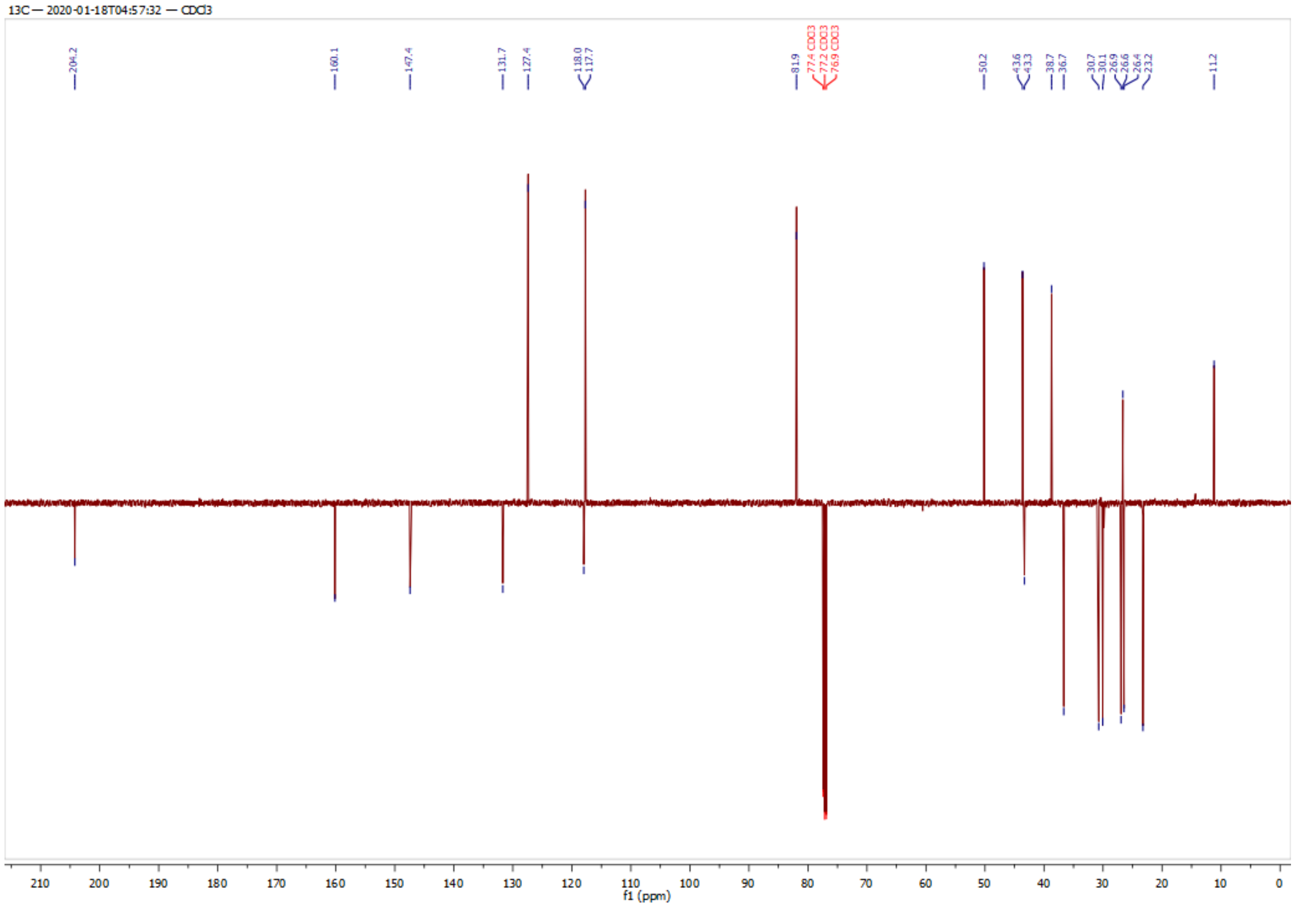
13C — 2020-01-18T04:57:32 — CDCl3
204.2
160.1
147.4
131.7
127.4
118.0
117.7
81.9
77.4 CDCl3
77.2 CDCl3
76.9 CDCl3
50.2
43.6
43.3
38.7
36.7
30.7
30.1
26.9
26.6
26.4
23.2
11.2
f1 (ppm)

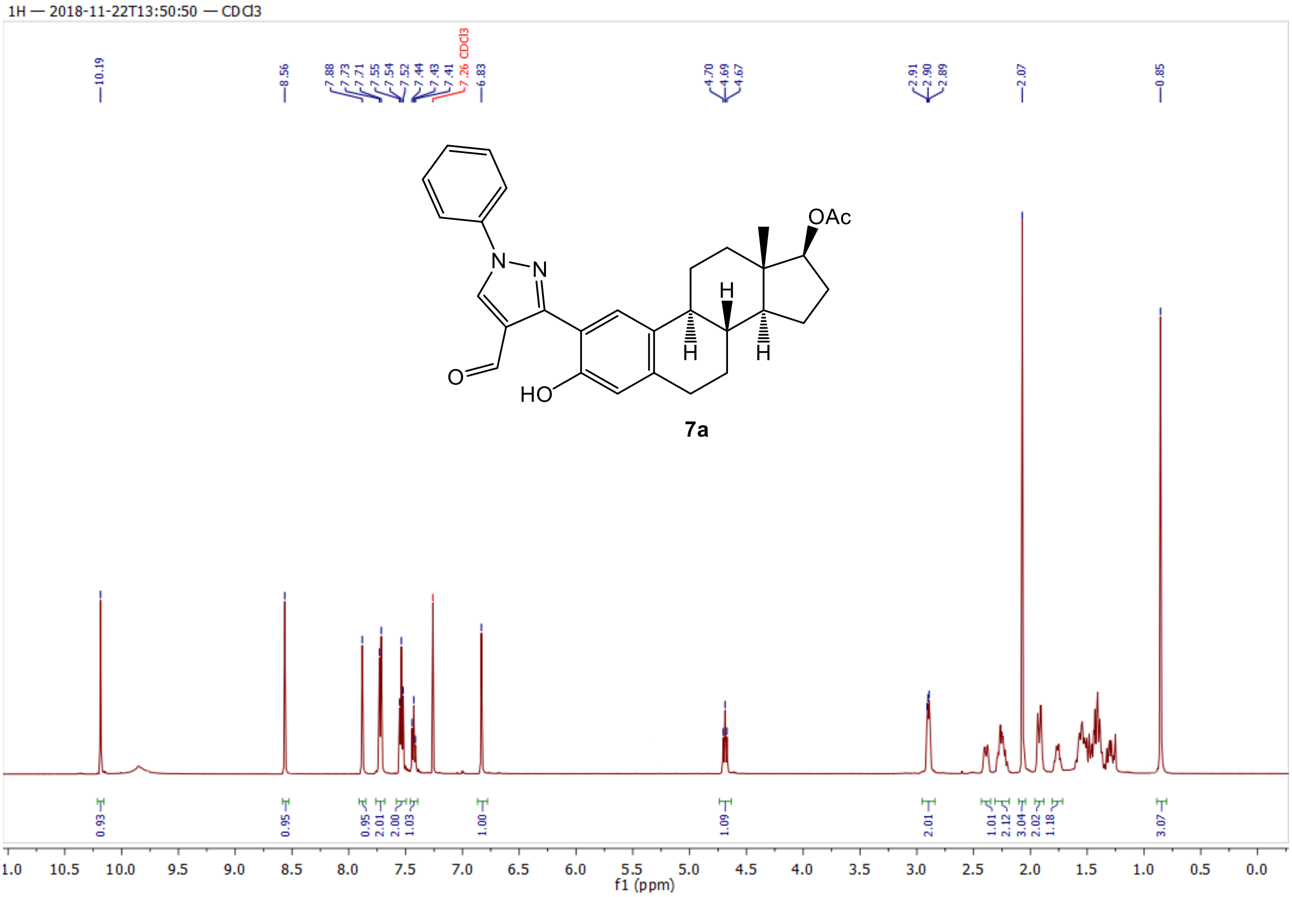
1H — 2018-11-22T13:50:50 — CDCl3
10.19
8.56
7.88
7.73
7.71
7.55
7.54
7.52
7.44
7.43
7.41
7.26 CDCl3
6.83
4.70
4.69
4.67
2.91
2.90
2.89
2.07
0.85
OAc
N
N
O
HO
H
H
H
7a
0.93
0.95
0.95
2.01
2.00
1.03
1.00
1.09
2.01
1.01
2.12
3.04
2.02
1.18
3.07
f1 (ppm)

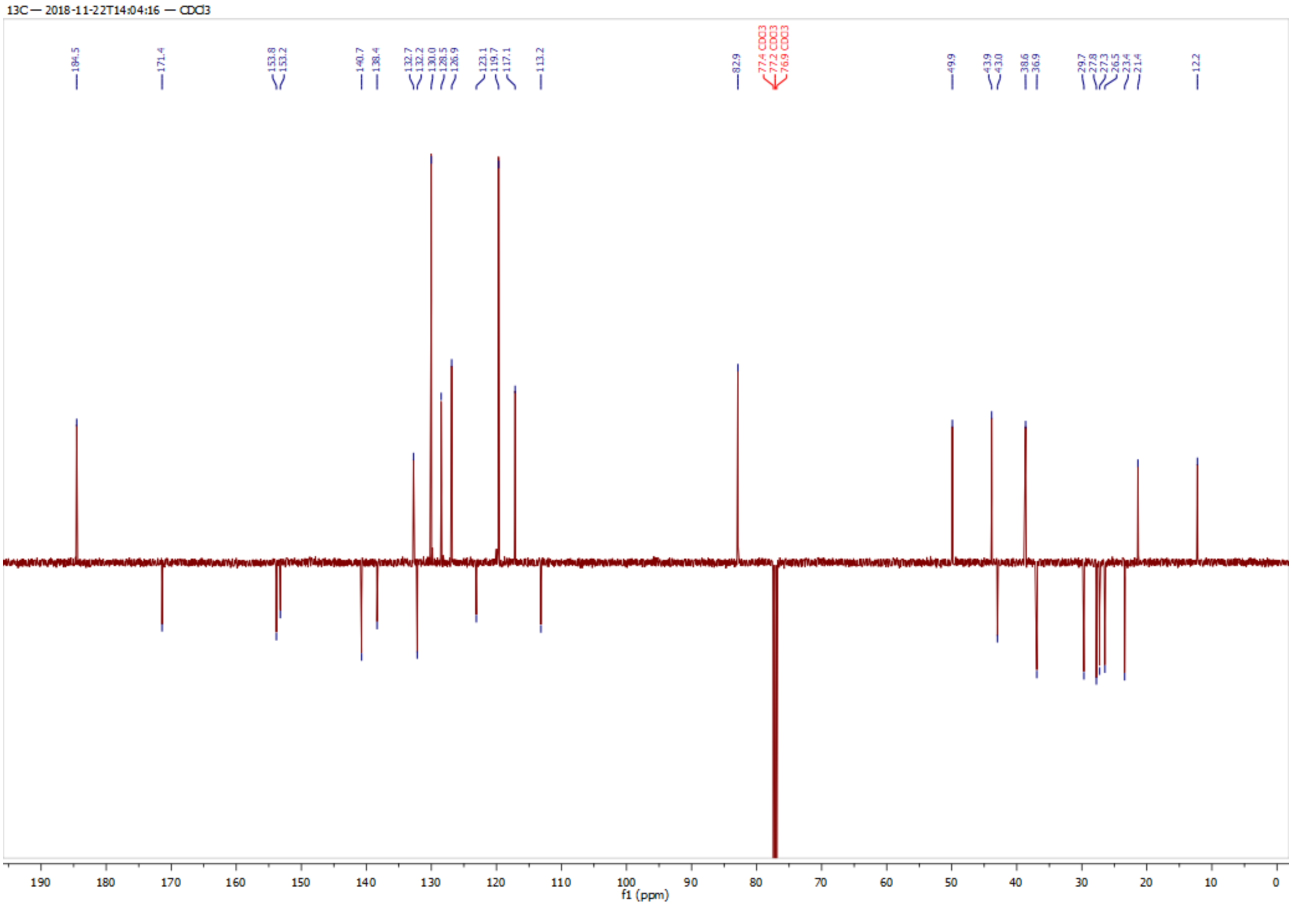
13C — 2018-11-22T14:04:16 — CDCl3
184.5
171.4
153.8
153.2
140.7
138.4
132.7
132.2
130.0
128.5
126.9
123.1
119.7
117.1
113.2
82.9
77.4 CDCl3
77.2 CDCl3
76.9 CDCl3
49.9
43.9
43.0
38.6
36.9
29.7
27.8
27.3
26.5
23.4
21.4
12.2
f1 (ppm)

1H — 2019-03-29T09:18:36 — CDCl3

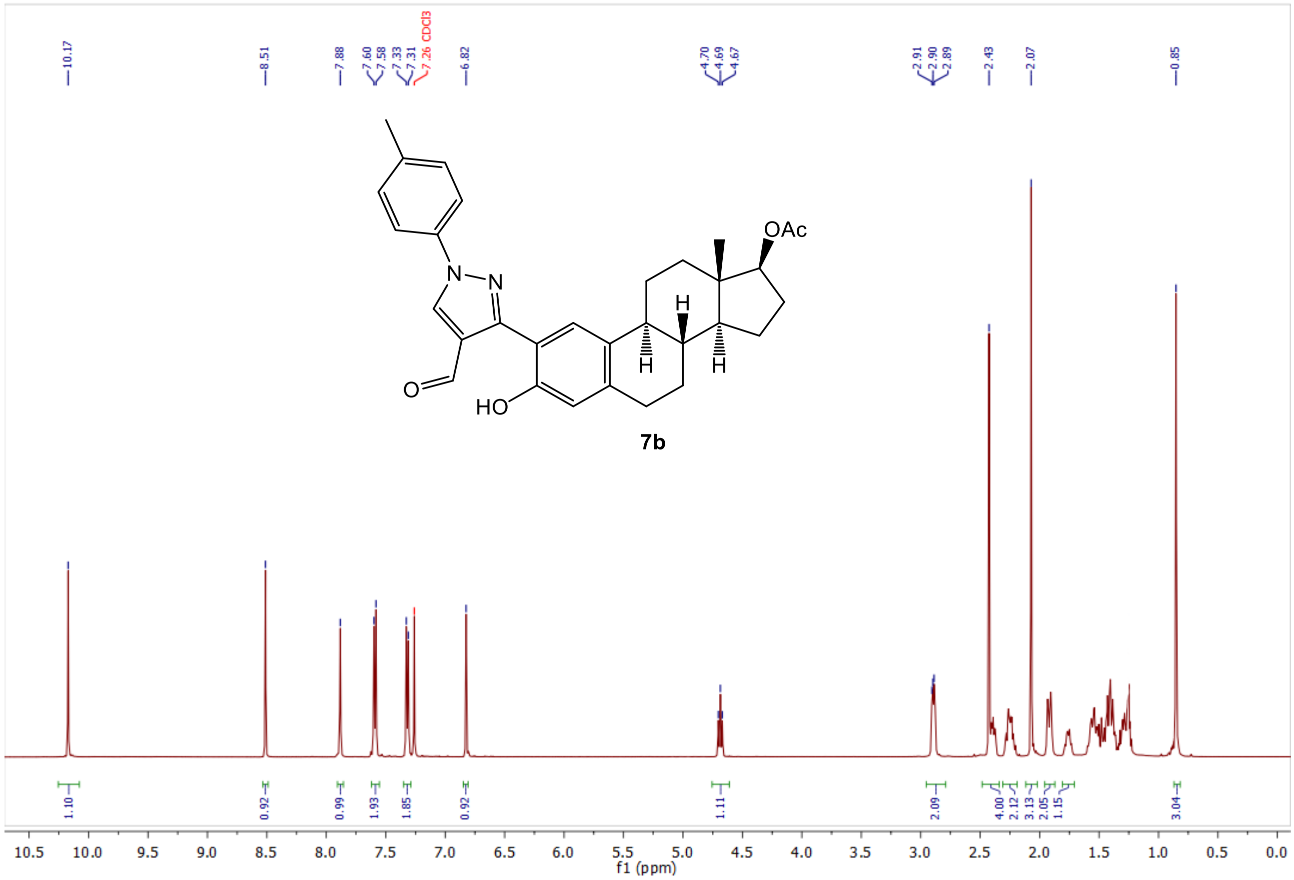

13C — 2019-03-29T09:32:00 — CDCl3

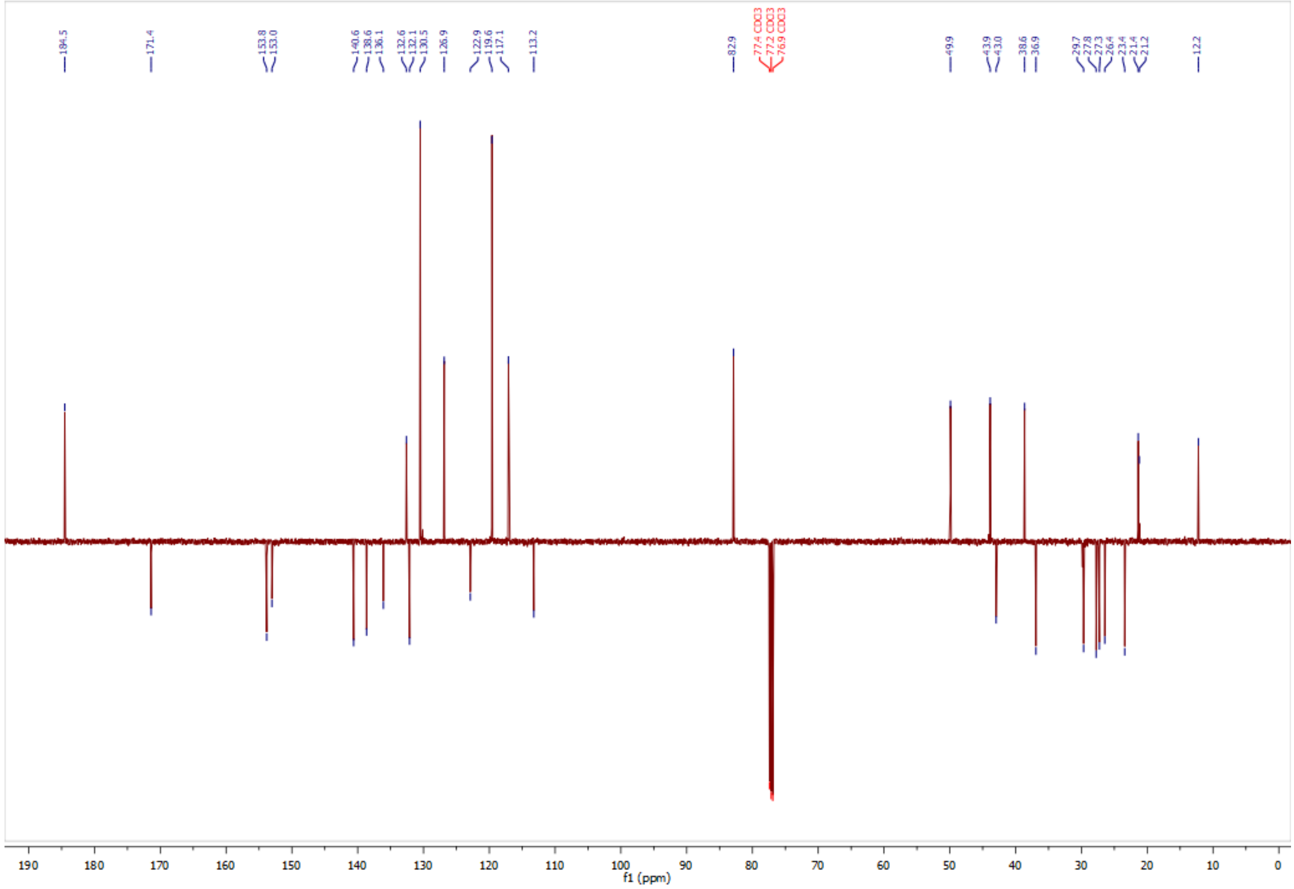

1H — 2019-03-15T04:13:12 — CDCl3

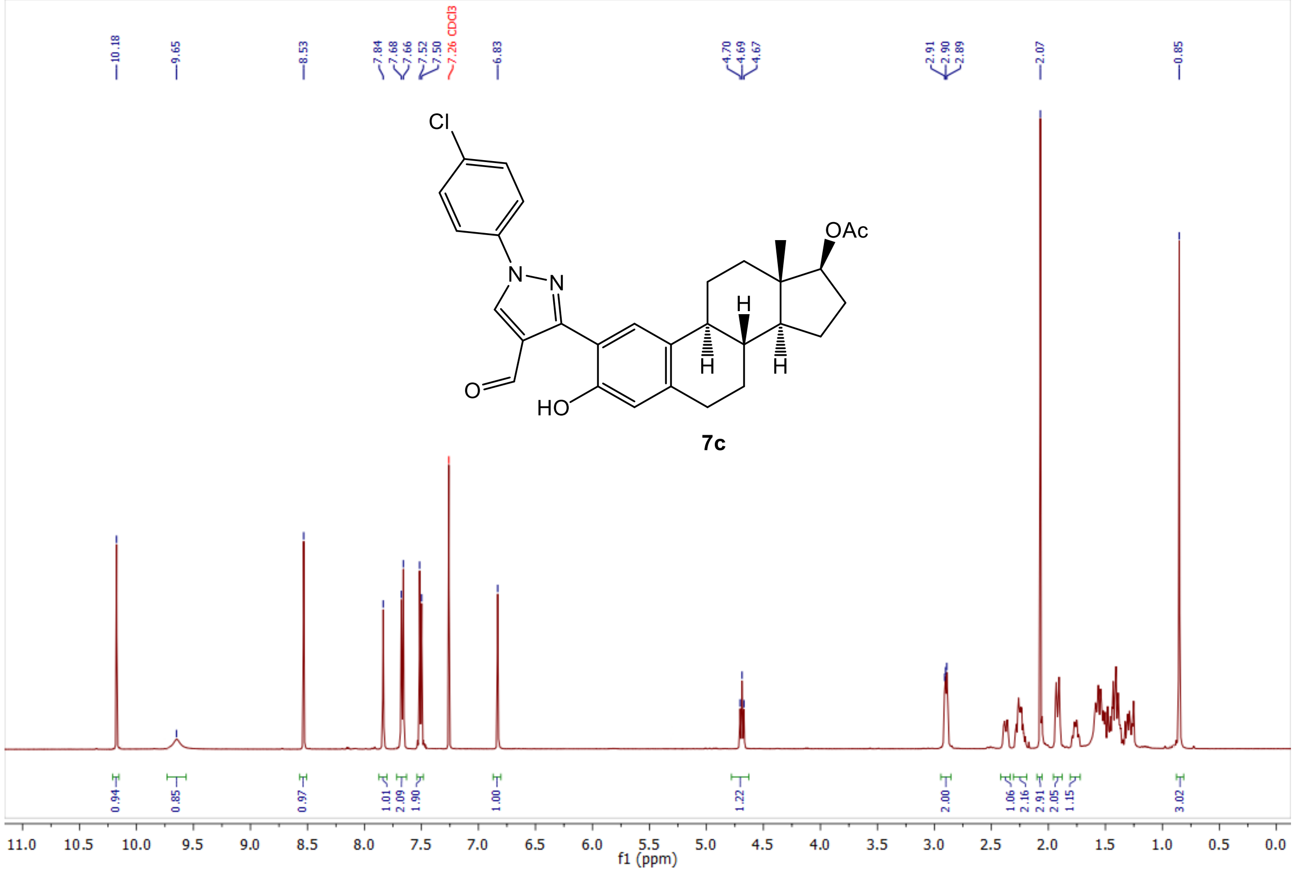

13C — 2019-03-15T04:26:36 — CDCl3

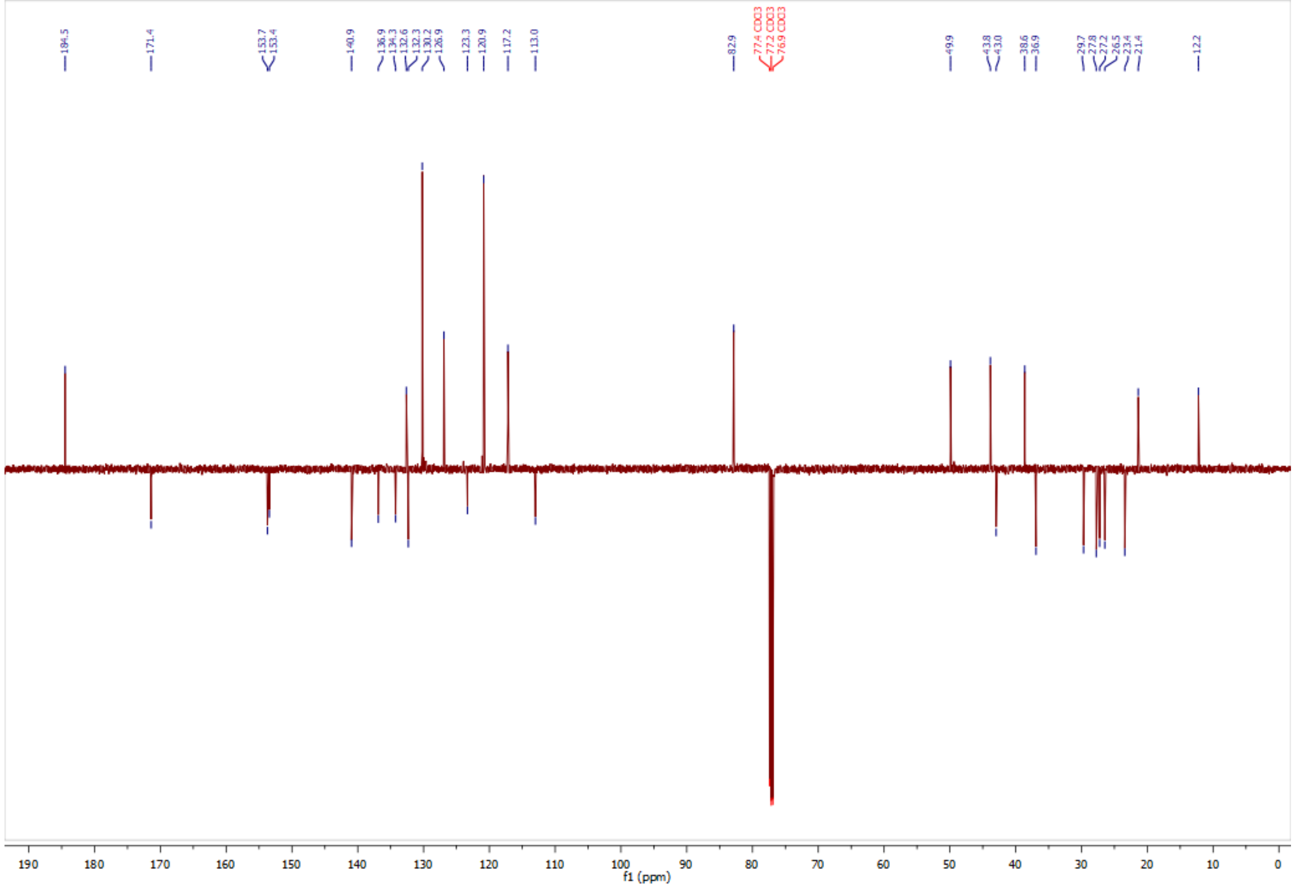

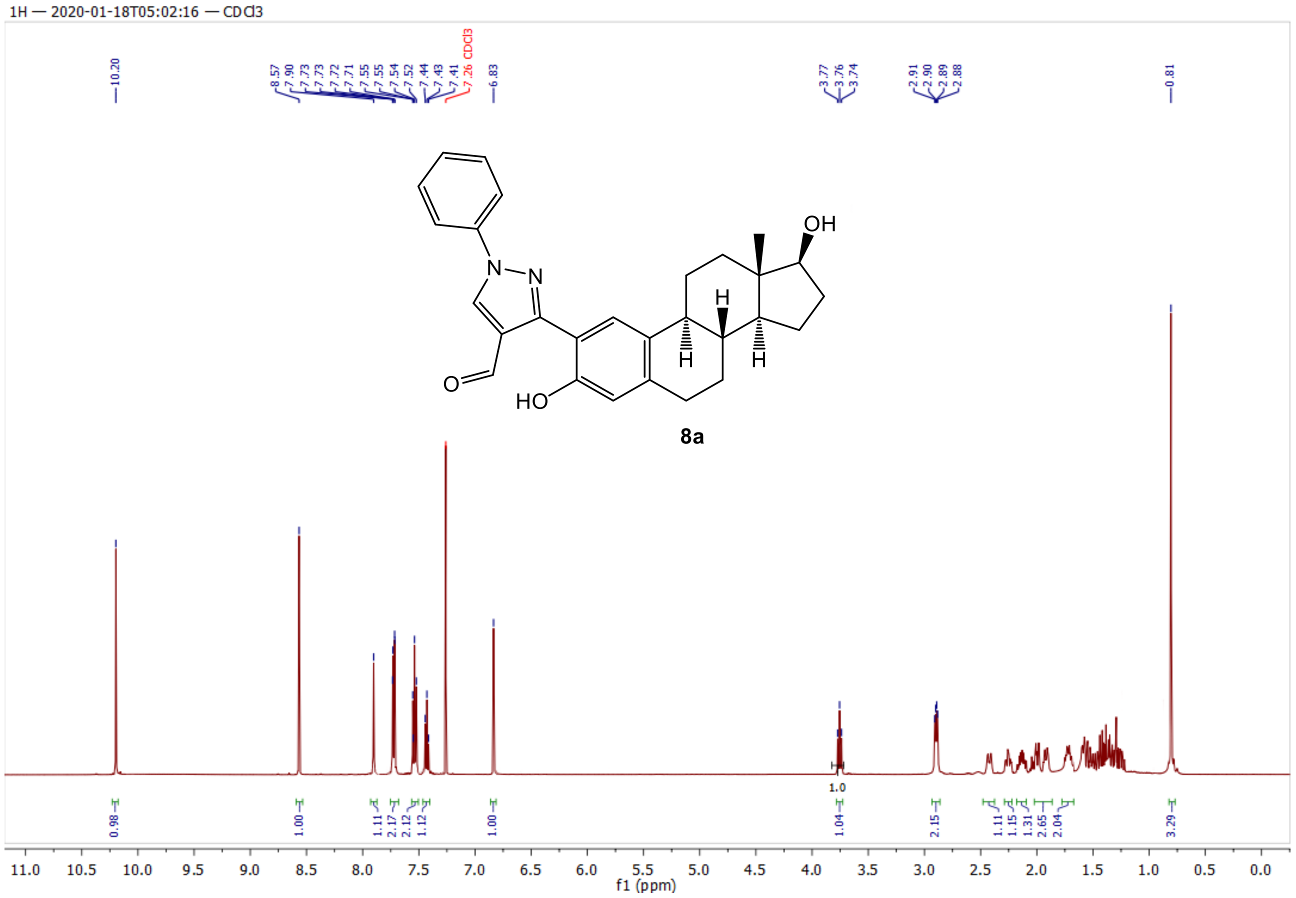
1H — 2020-01-18T05:02:16 — CDCl3
10.20
8.57
7.90
7.73
7.73
7.72
7.71
7.55
7.55
7.54
7.52
7.44
7.43
7.41
7.26 CDCl3
6.83
3.77
3.76
3.74
2.91
2.90
2.89
2.88
0.81
OH
N
N
H
H
H
O
HO
8a
1.0
0.98
1.00
1.11
2.17
2.12
1.12
1.00
1.04
2.15
1.11
1.15
1.31
2.65
2.04
3.29
f1 (ppm)

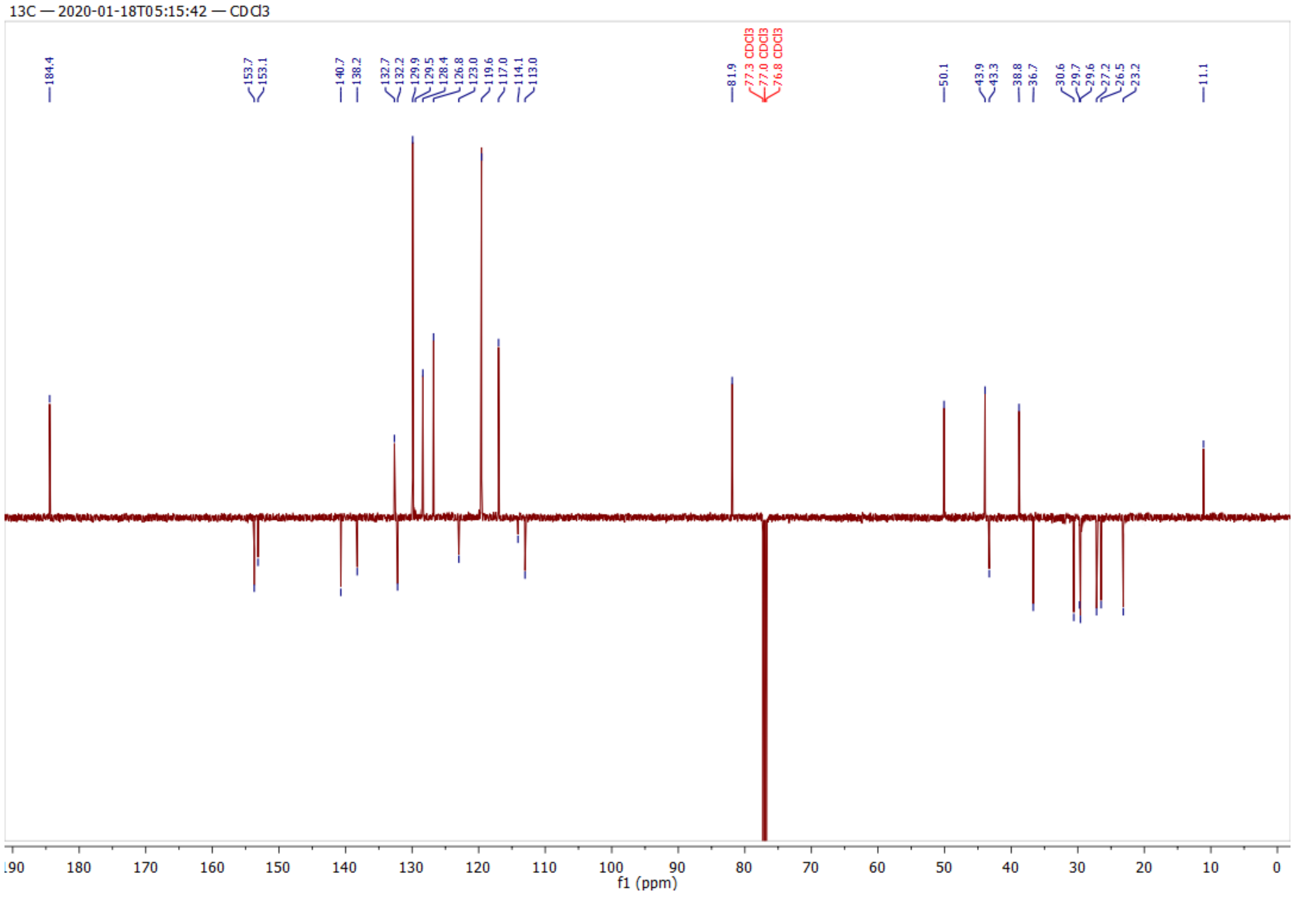
13C — 2020-01-18T05:15:42 — CDCl3
184.4
153.7
153.1
140.7
138.2
132.7
132.2
129.9
129.5
128.4
126.8
123.0
119.6
117.0
114.1
113.0
81.9
77.3 CDCl3
77.0 CDCl3
76.8 CDCl3
50.1
43.9
43.3
38.8
36.7
30.6
29.7
29.6
27.2
26.5
23.2
11.1
f1 (ppm)

1H — 2020-01-18T05:20:28 — CDCl3

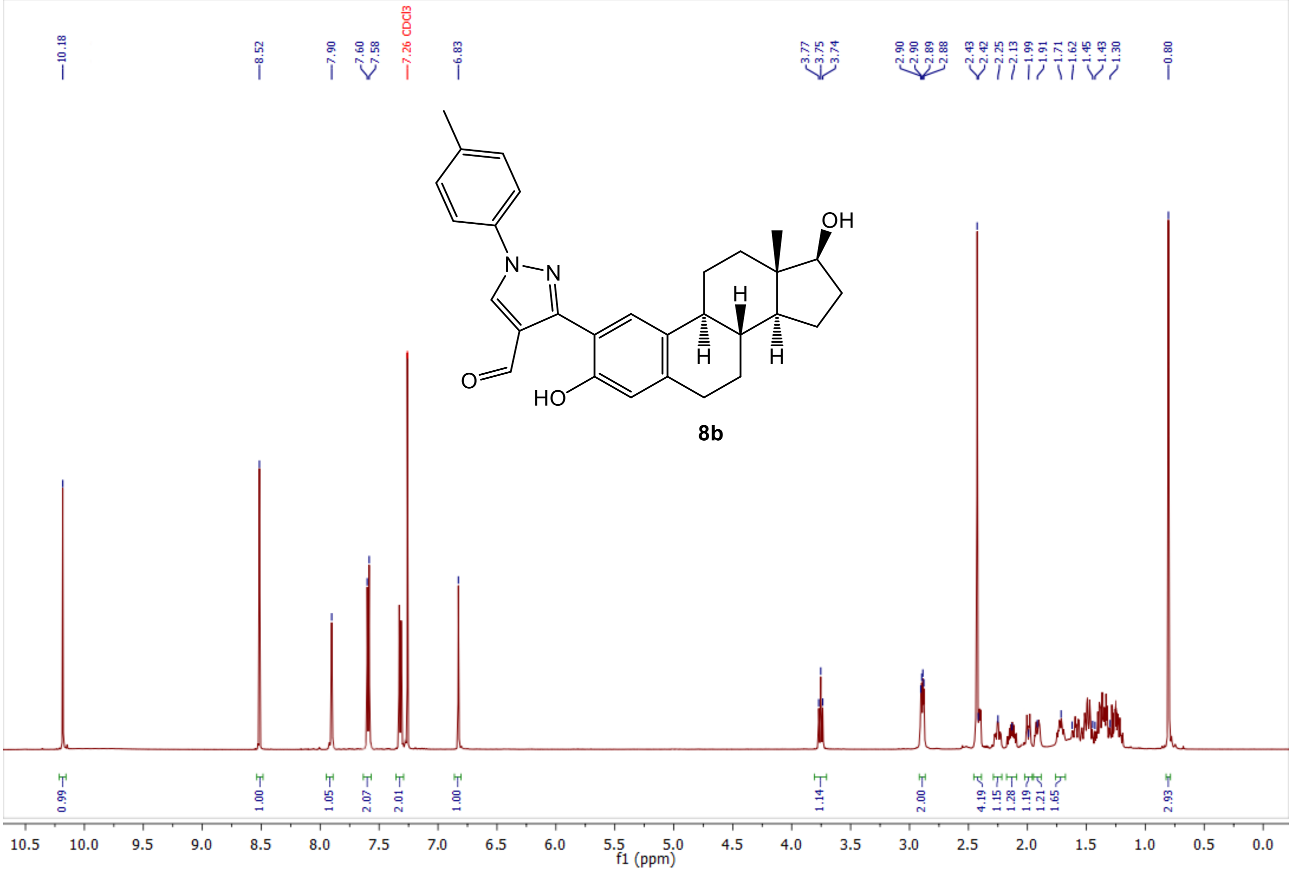

13C — 2020-01-18T05:33:53 — CDCl3

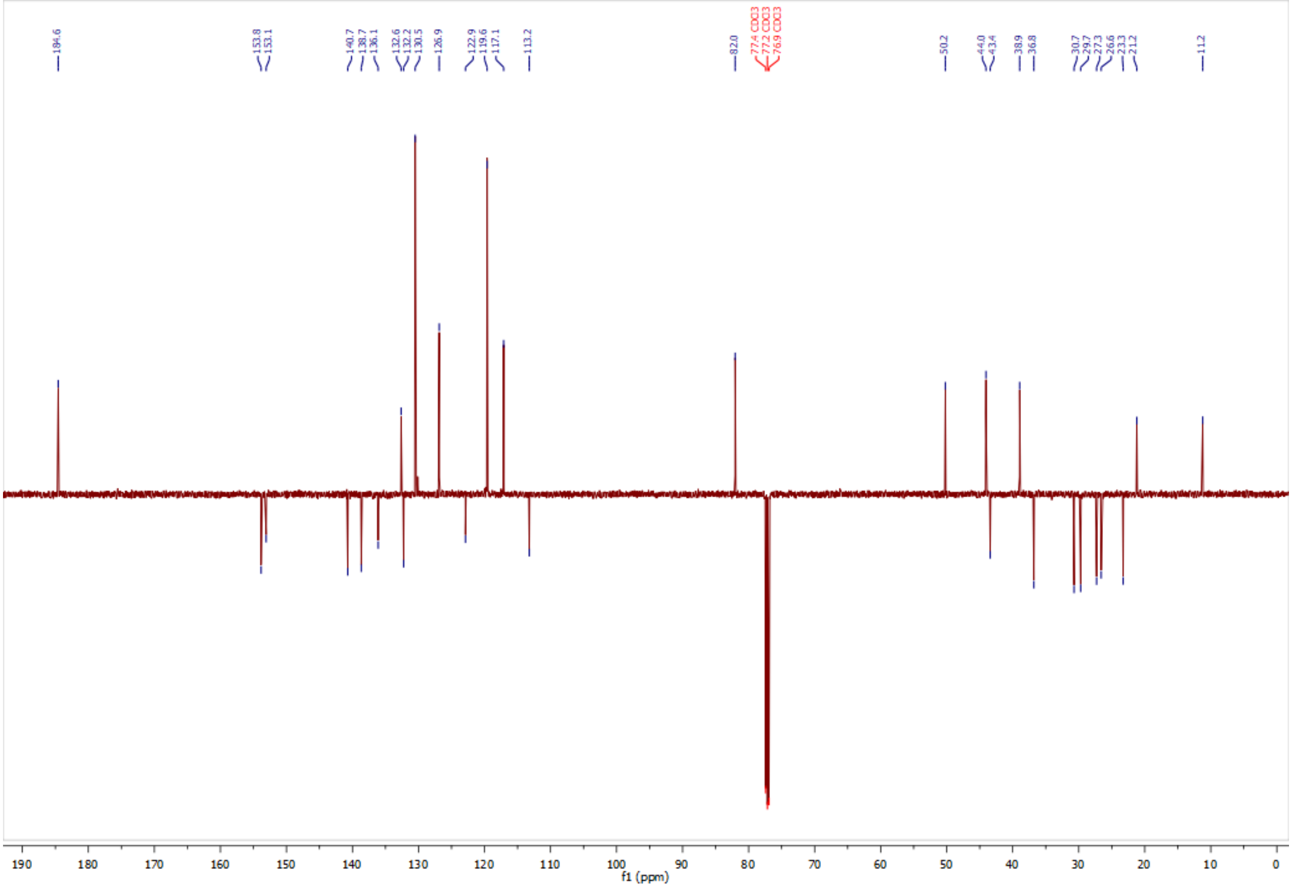

1H — 2020-01-18T05:38:54 — CDCl3

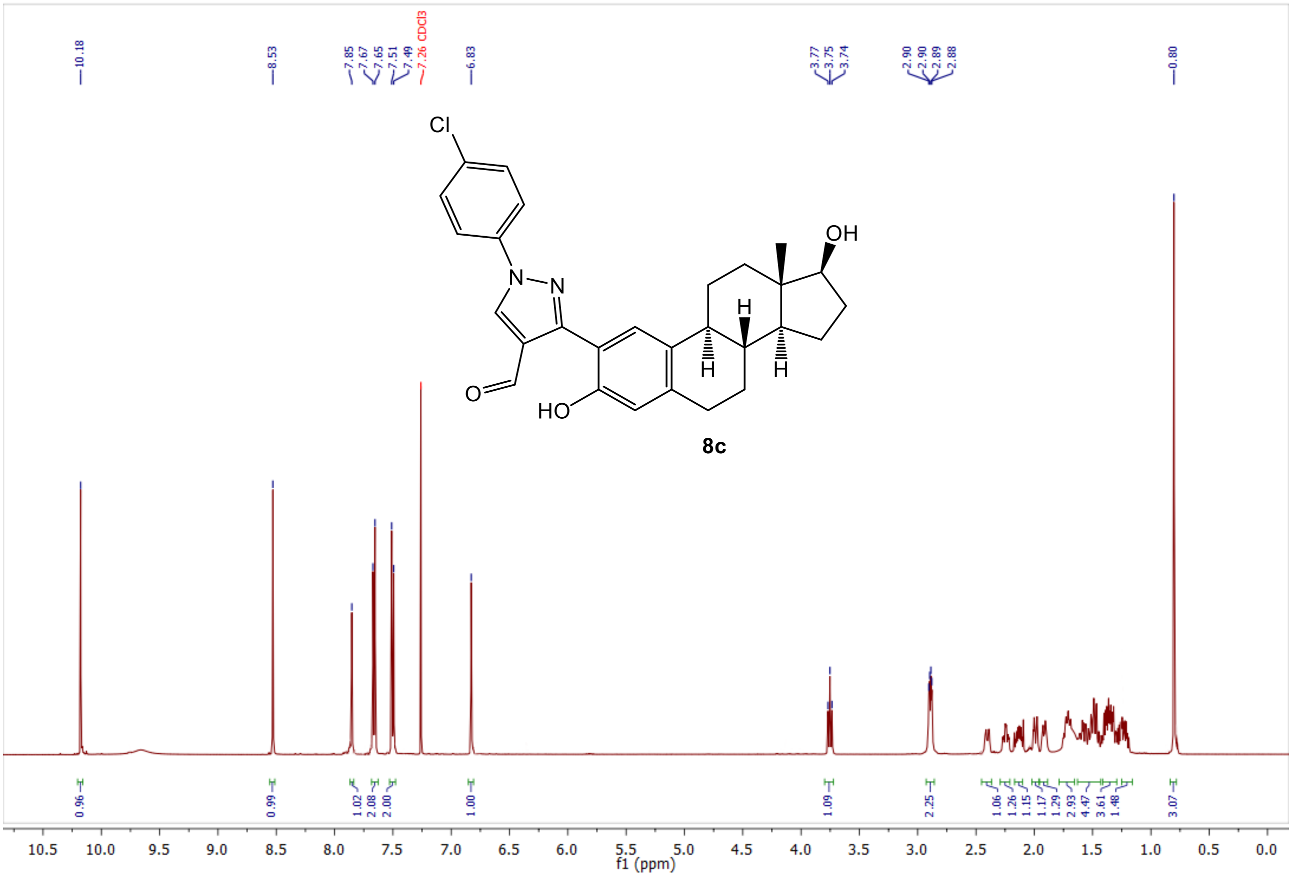

13C — 2020-01-18T05:52:19 — CDCl3

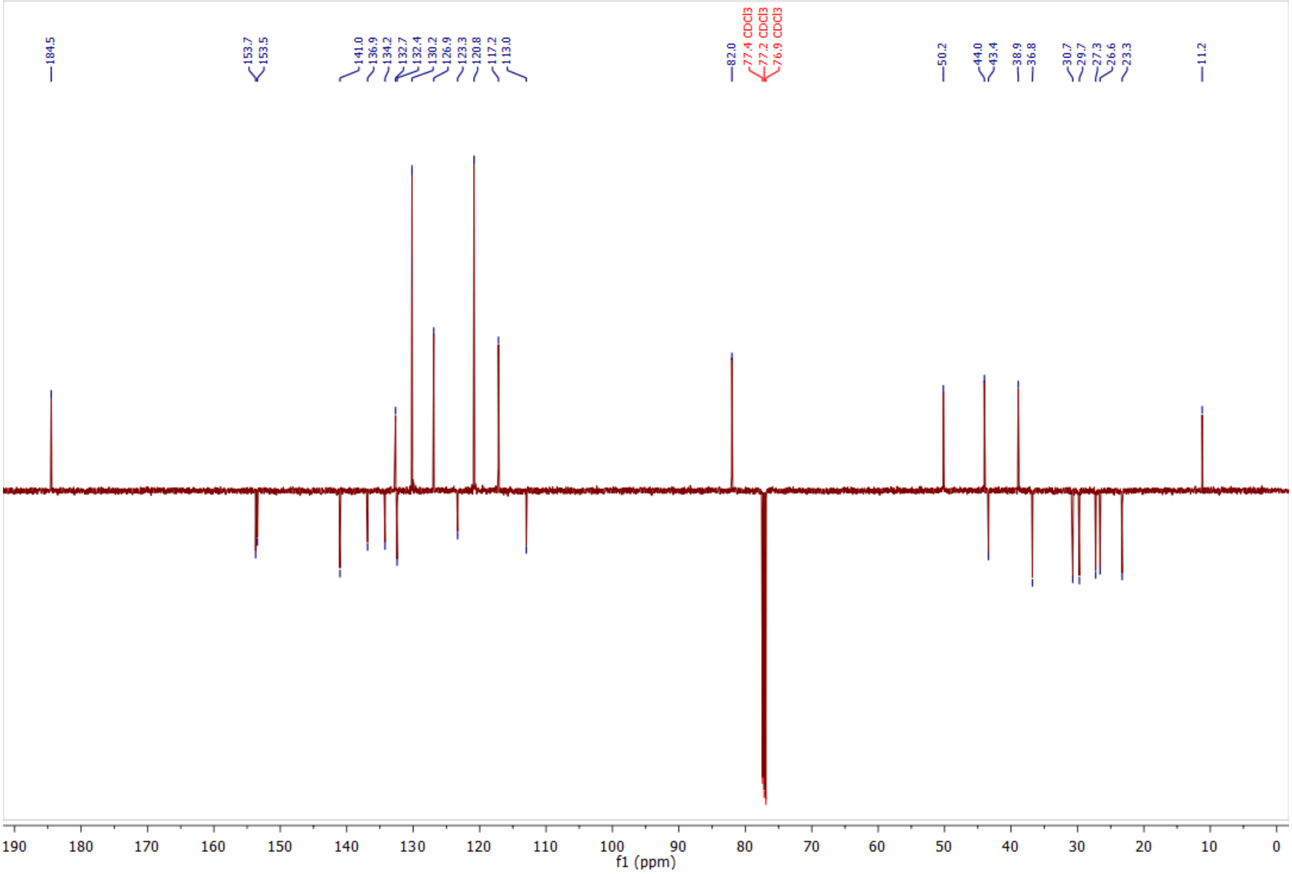

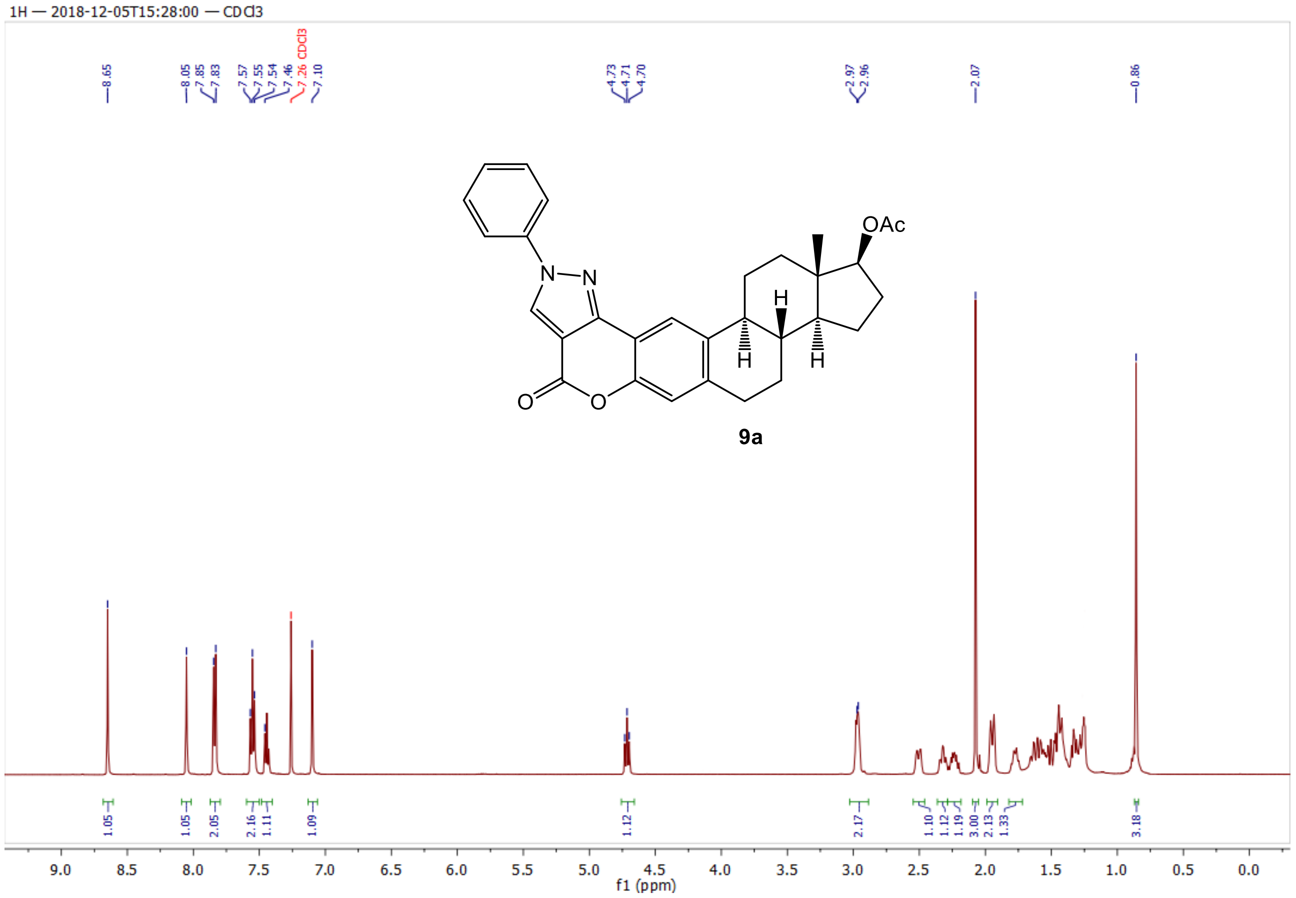
1H — 2018-12-05T15:28:00 — CDCl3
8.65
8.05
7.85
7.83
7.57
7.55
7.54
7.46
7.26 CDCl3
7.10
4.73
4.71
4.70
2.97
2.96
2.07
0.86
OAc
N
N
H
H
H
O
O
9a
1.05
1.05
2.05
2.16
1.11
1.09
1.12
2.17
1.10
1.12
1.19
3.00
2.13
1.33
3.18
9.0 8.5 8.0 7.5 7.0 6.5 6.0 5.5 5.0 4.5 4.0 3.5 3.0 2.5 2.0 1.5 1.0 0.5 0.0
f1 (ppm)

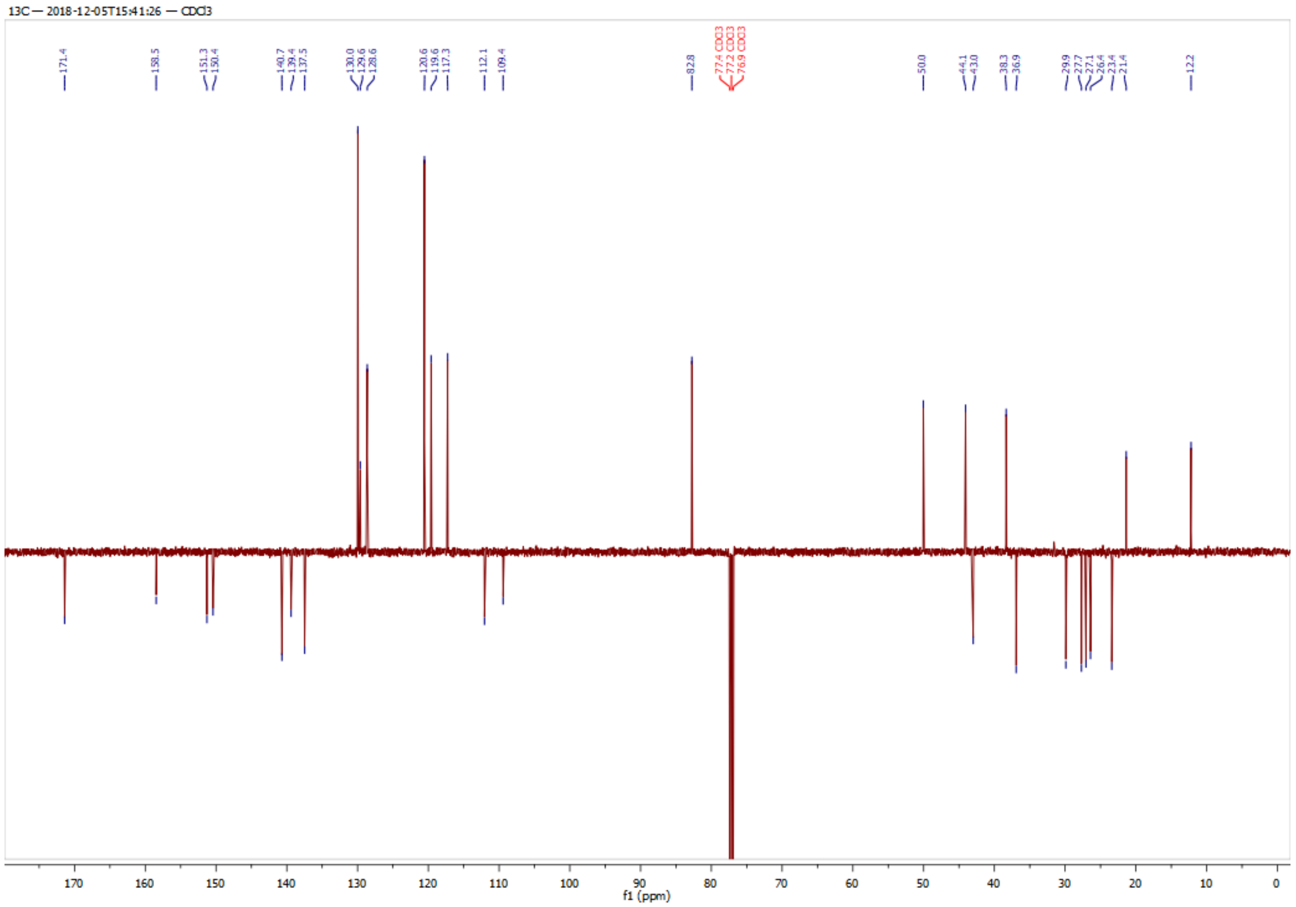
13C — 2018-12-05T15:41:26 — CDCl3
171.4
158.5
151.3
150.4
140.7
139.4
137.5
130.0
129.6
128.6
120.6
119.6
117.3
112.1
109.4
82.8
77.4 CDCl3
77.2 CDCl3
76.9 CDCl3
50.0
44.1
43.0
38.3
36.9
29.9
27.7
27.1
26.4
23.4
21.4
12.2
170 160 150 140 130 120 110 100 90 80 70 60 50 40 30 20 10 0
f1 (ppm)

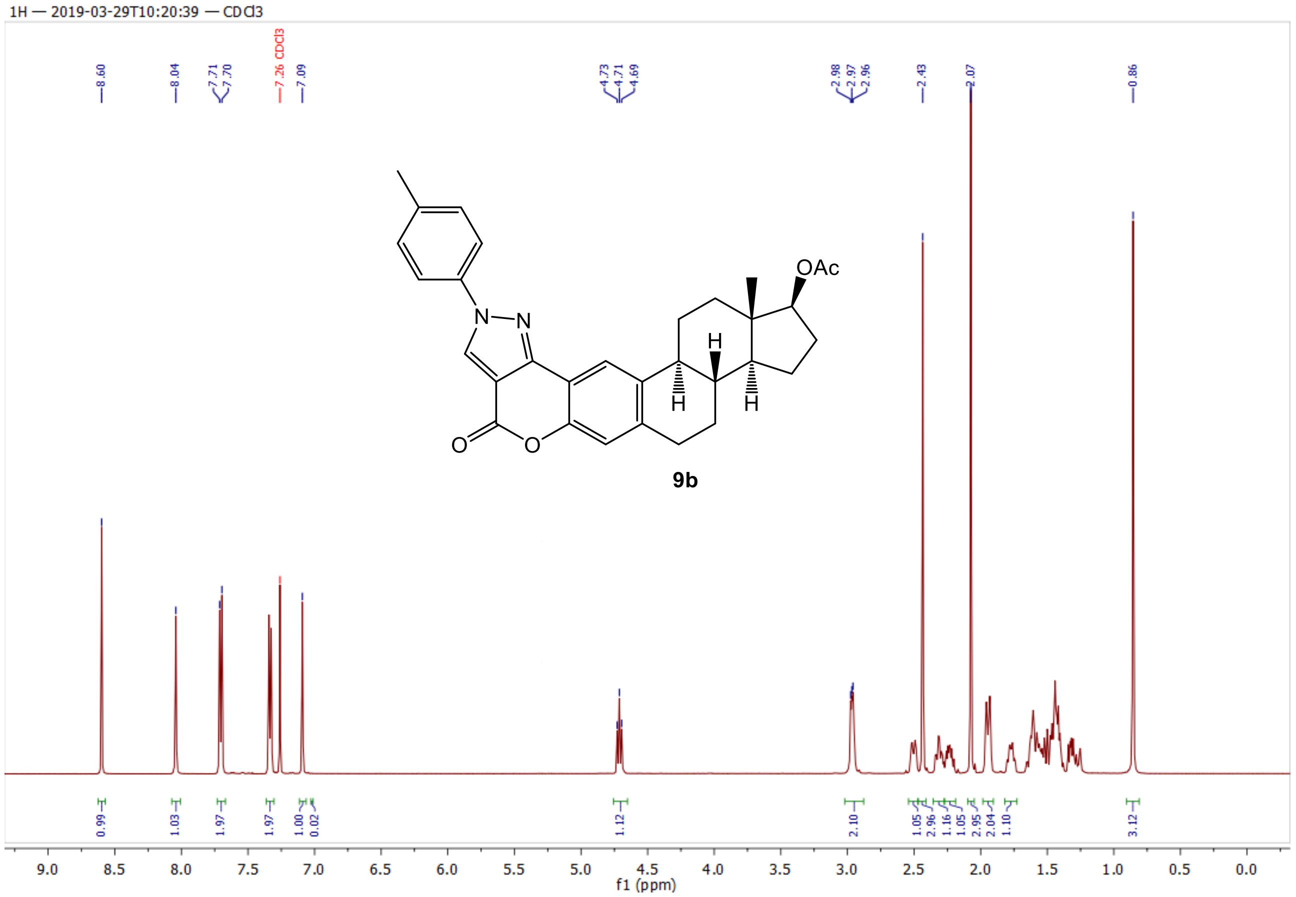
1H — 2019-03-29T10:20:39 — CDCl3
OAc
9b
f1 (ppm)

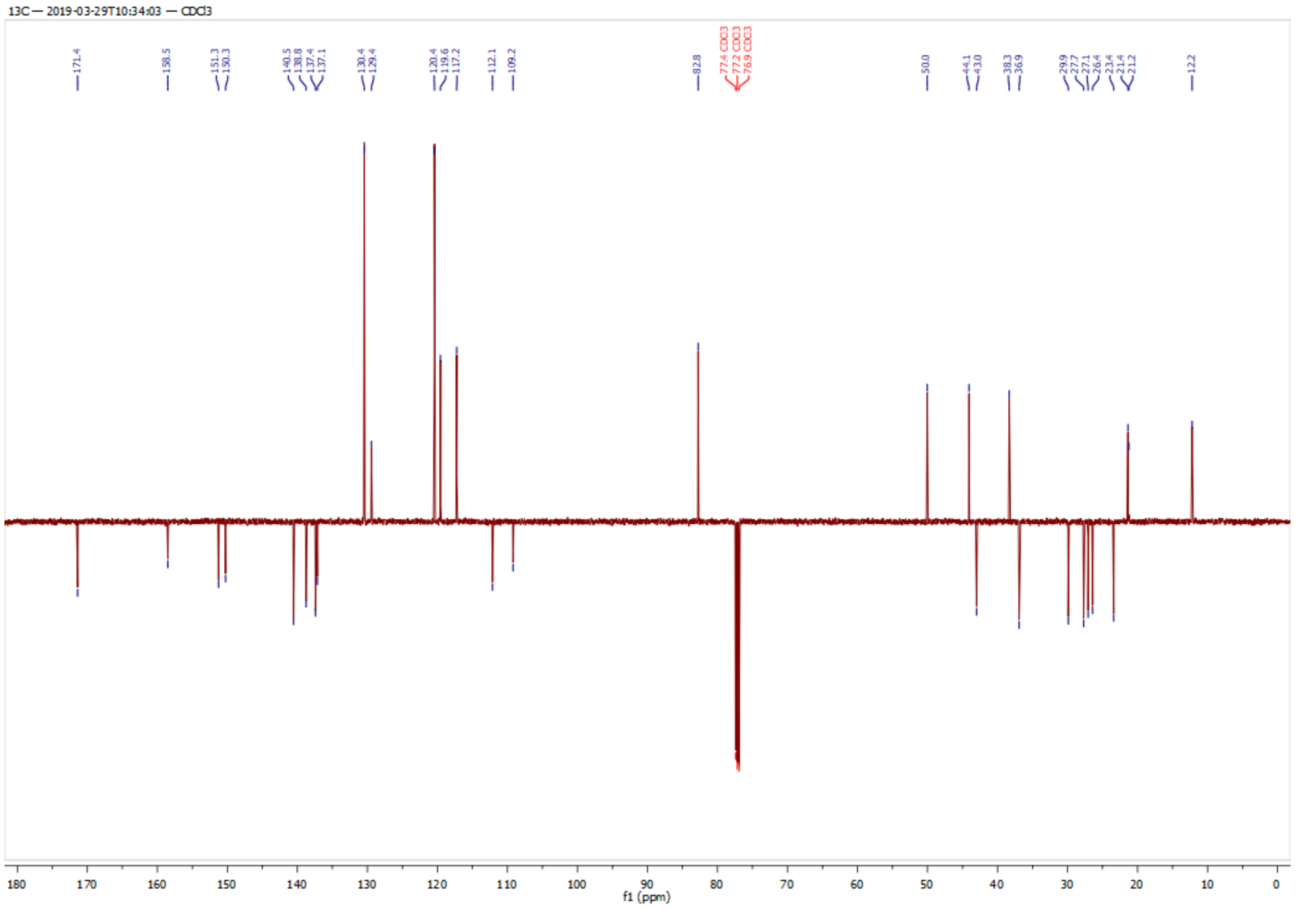
13C — 2019-03-29T10:34:03 — CDCl3
f1 (ppm)

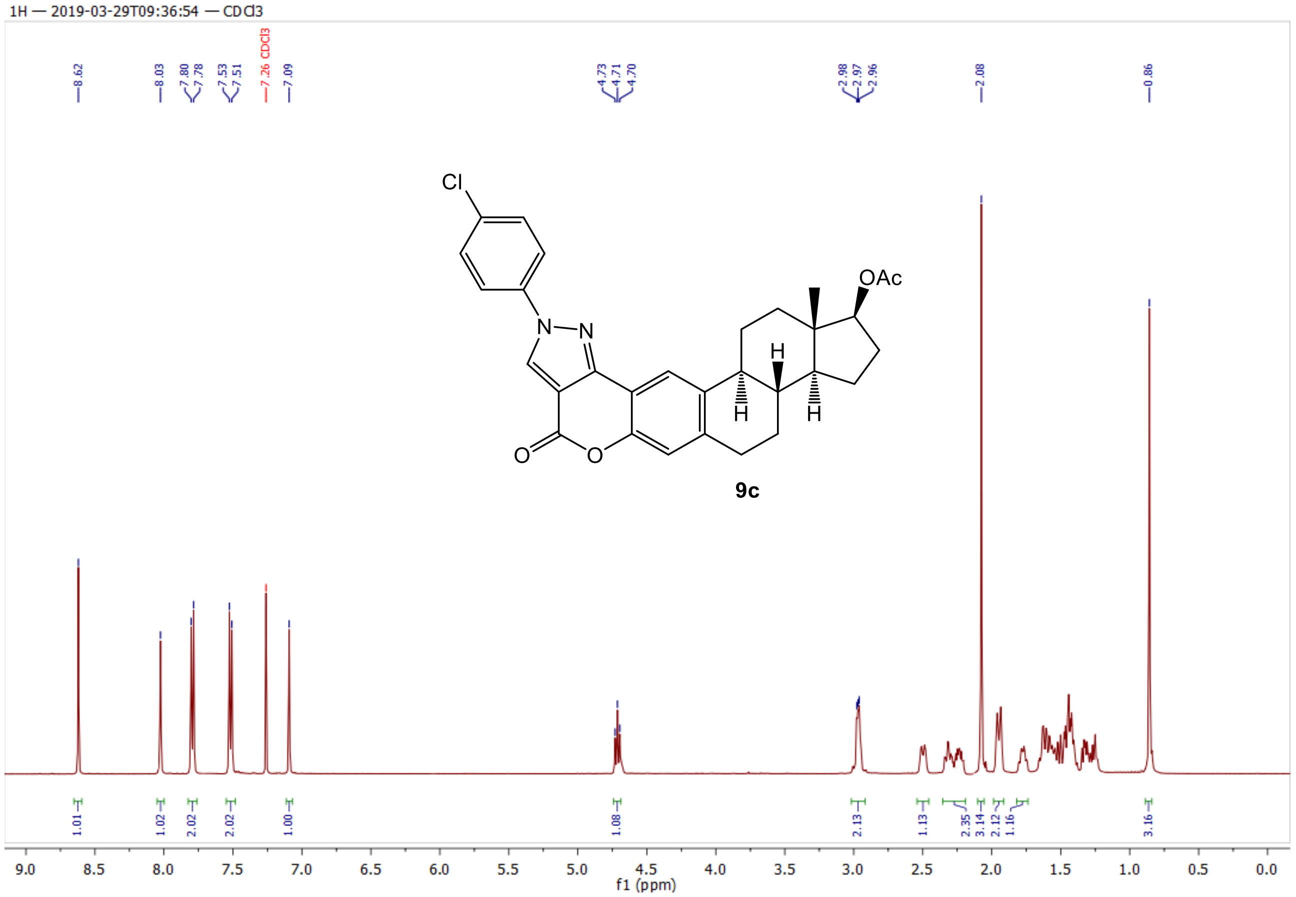
1H — 2019-03-29T09:36:54 — CDCl3
8.62
8.03
7.80
7.78
7.53
7.51
7.26 CDCl3
7.09
4.73
4.71
4.70
2.98
2.97
2.96
2.08
0.86
Cl
N
N
O
O
OAc
H
H
H
9c
1.01
1.02
2.02
2.02
1.00
1.08
2.13
1.13
2.35
3.14
2.12
1.16
3.16
f1 (ppm)

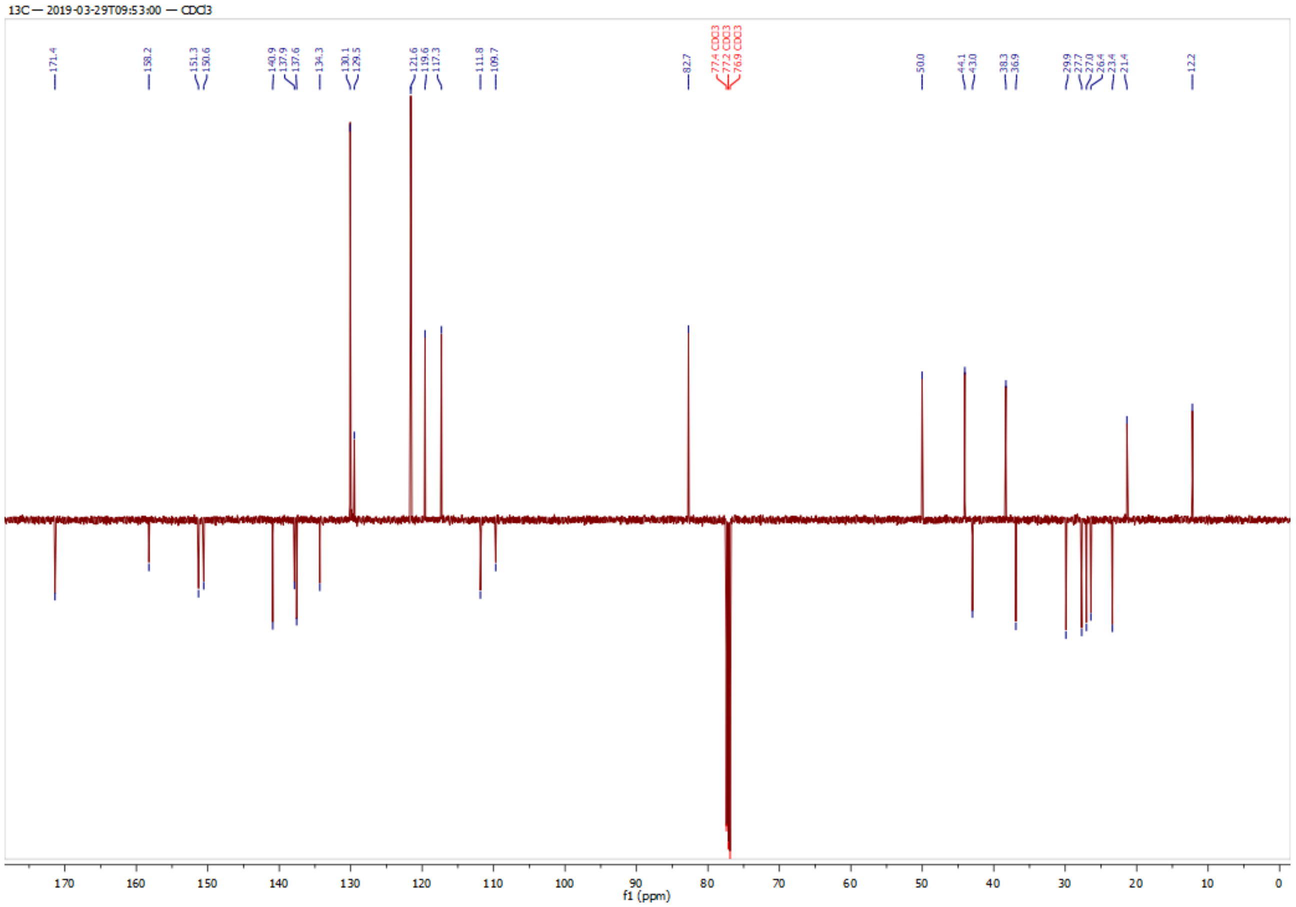
13C — 2019-03-29T09:53:00 — CDCl3
171.4
158.2
151.3
150.6
140.9
137.9
137.6
134.3
130.1
129.5
121.6
119.6
117.3
111.8
109.7
82.7
77.4 CDCl3
77.2 CDCl3
76.9 CDCl3
50.0
44.1
43.0
38.3
36.9
29.9
27.7
27.0
26.4
23.4
21.4
12.2
f1 (ppm)

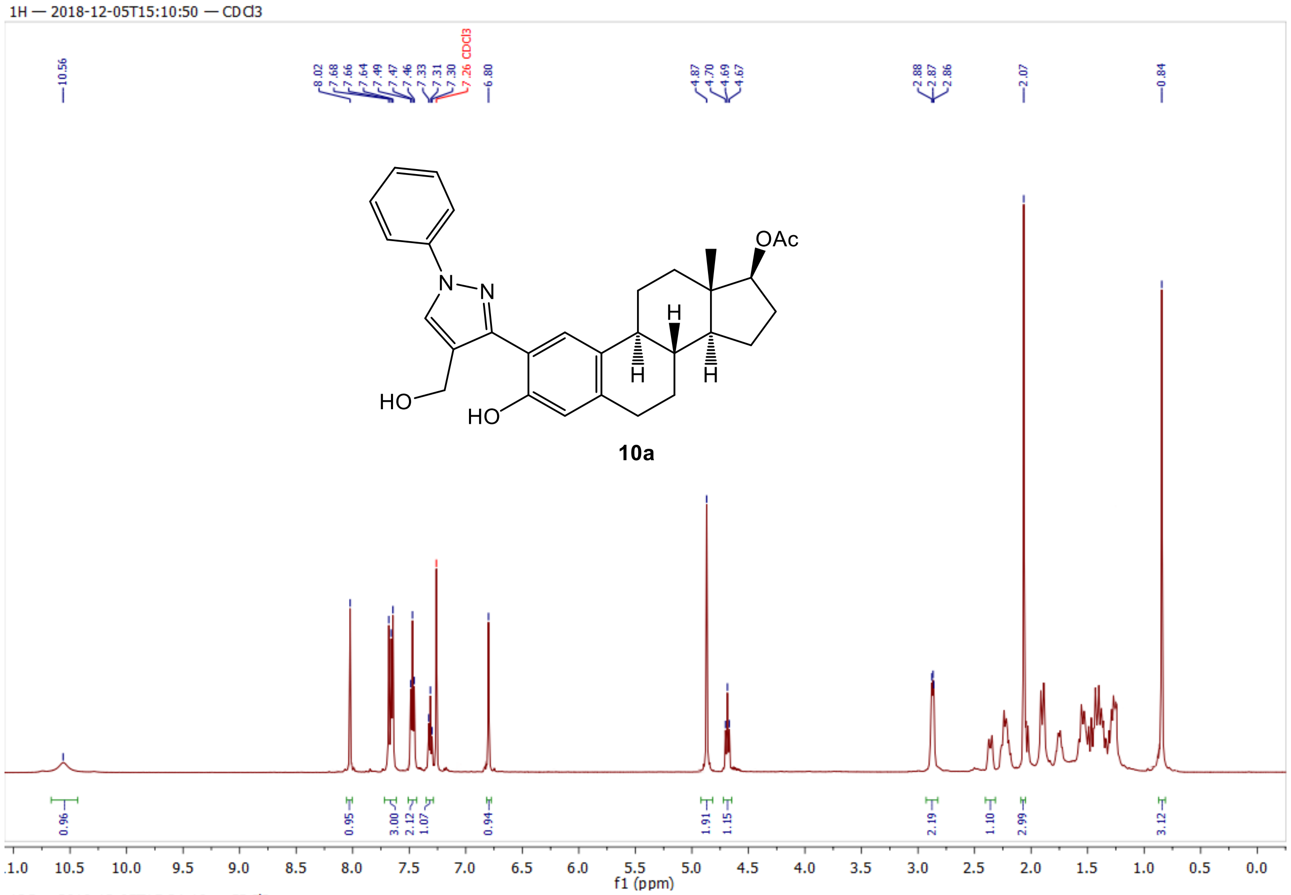
1H — 2018-12-05T15:10:50 — CDCl3
10.56
8.02 7.68 7.66 7.64 7.49 7.47 7.46 7.33 7.31 7.30 7.26 CDCl3 6.80
4.87 4.70 4.69 4.67
2.88 2.87 2.86
2.07
0.84
OAc
N
N
H
H
H
HO
HO
10a
0.96 0.95 3.00 2.12 1.07 0.94 1.91 1.15 2.19 1.10 2.99 3.12
f1 (ppm)

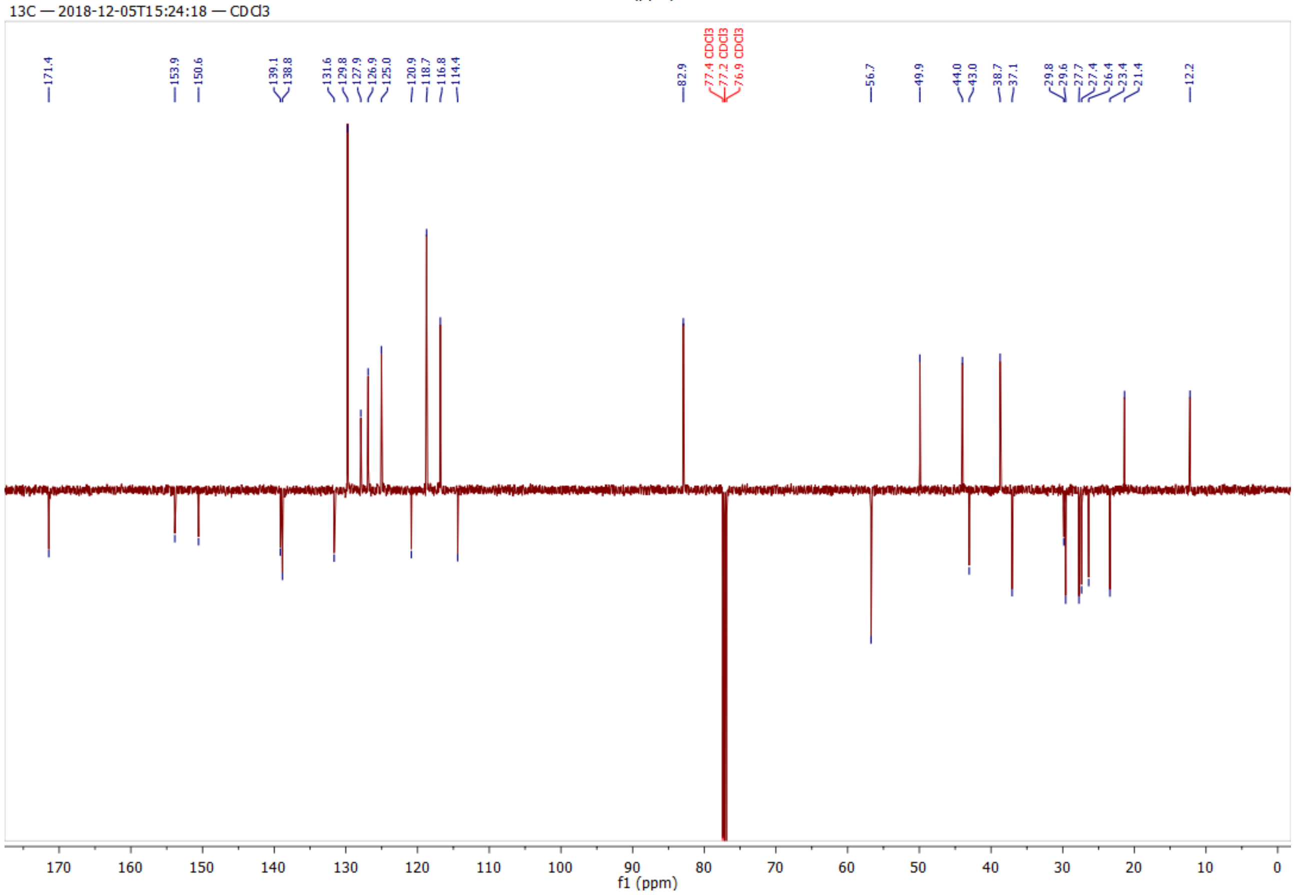
13C — 2018-12-05T15:24:18 — CDCl3
171.4
153.9 150.6
139.1 138.8
131.6 129.8 127.9 126.9 125.0
120.9 118.7 116.8 114.4
82.9
77.4 CDCl3 77.2 CDCl3 76.9 CDCl3
56.7
49.9
44.0 43.0
38.7 37.1
29.8 29.6 27.7 27.4 26.4 23.4 21.4
12.2
f1 (ppm)

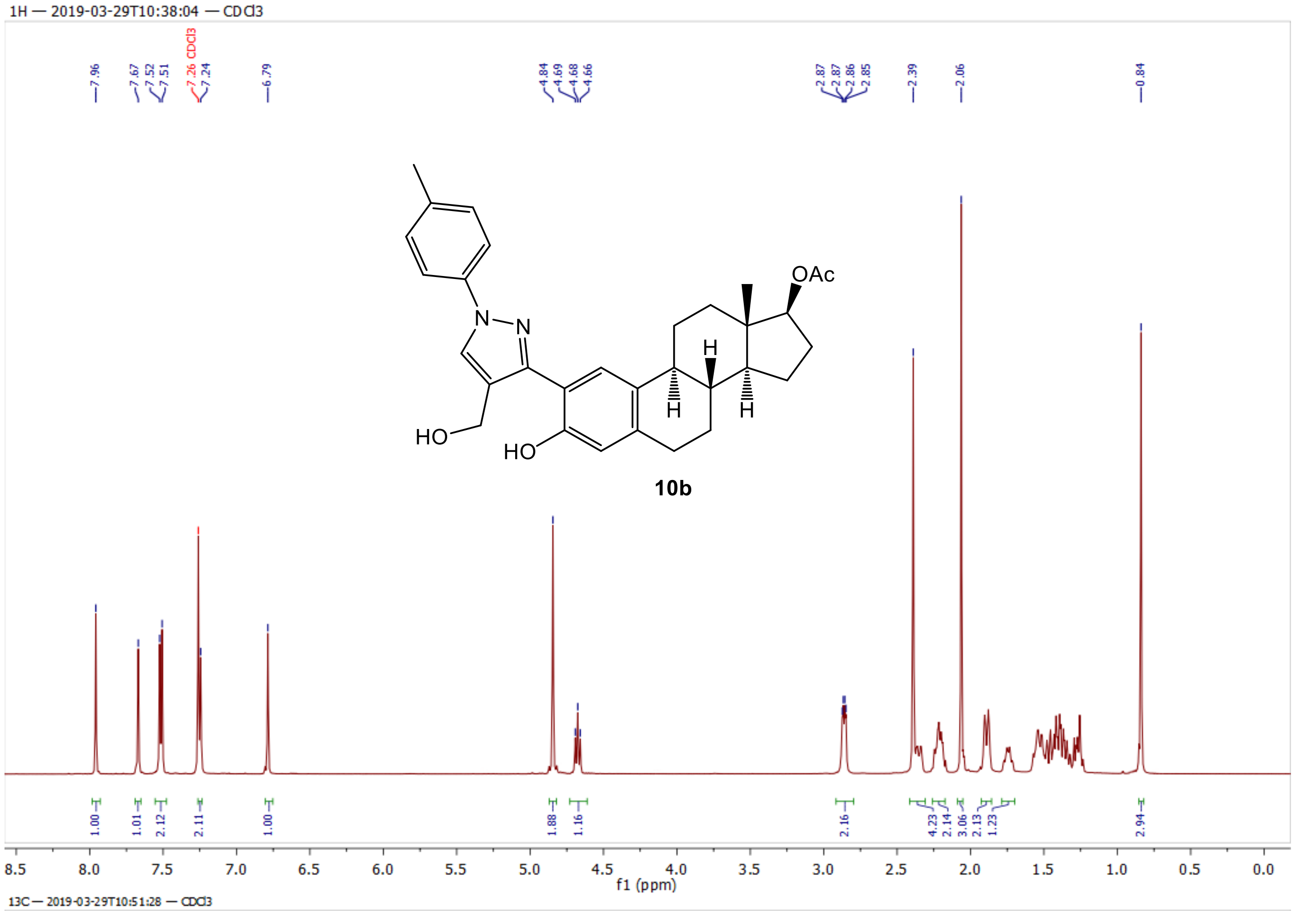
1H — 2019-03-29T10:38:04 — CDCl3
7.96
7.67
7.52
7.51
7.26 CDCl3
7.24
6.79
4.84
4.69
4.68
4.66
2.87
2.87
2.86
2.85
2.39
2.06
0.84
OAc
N
N
H
HO
HO
H
H
1.00
1.01
2.12
2.11
1.00
1.88
1.16
2.16
4.23
2.14
3.06
2.13
1.23
2.94
f1 (ppm)

10b

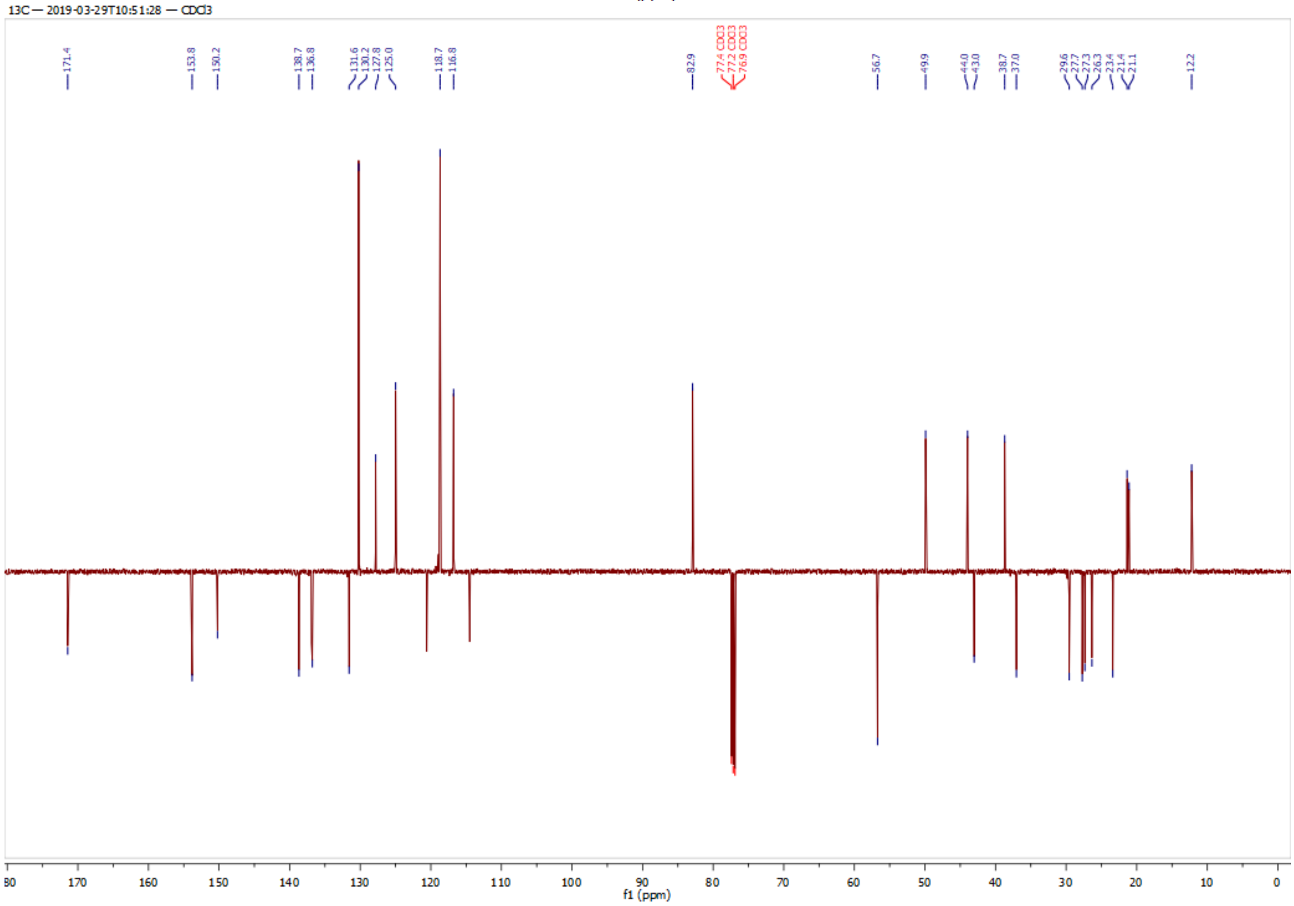
13C — 2019-03-29T10:51:28 — CDCl3
171.4
153.8
150.2
138.7
136.8
131.6
130.2
127.8
125.0
118.7
116.8
82.9
77.4 CDCl3
77.2 CDCl3
76.9 CDCl3
56.7
49.9
44.0
43.0
38.7
37.0
29.6
27.7
27.3
26.3
23.4
21.4
21.1
12.2
f1 (ppm)

1H — 2019-03-29T10:02:08 — DMSO

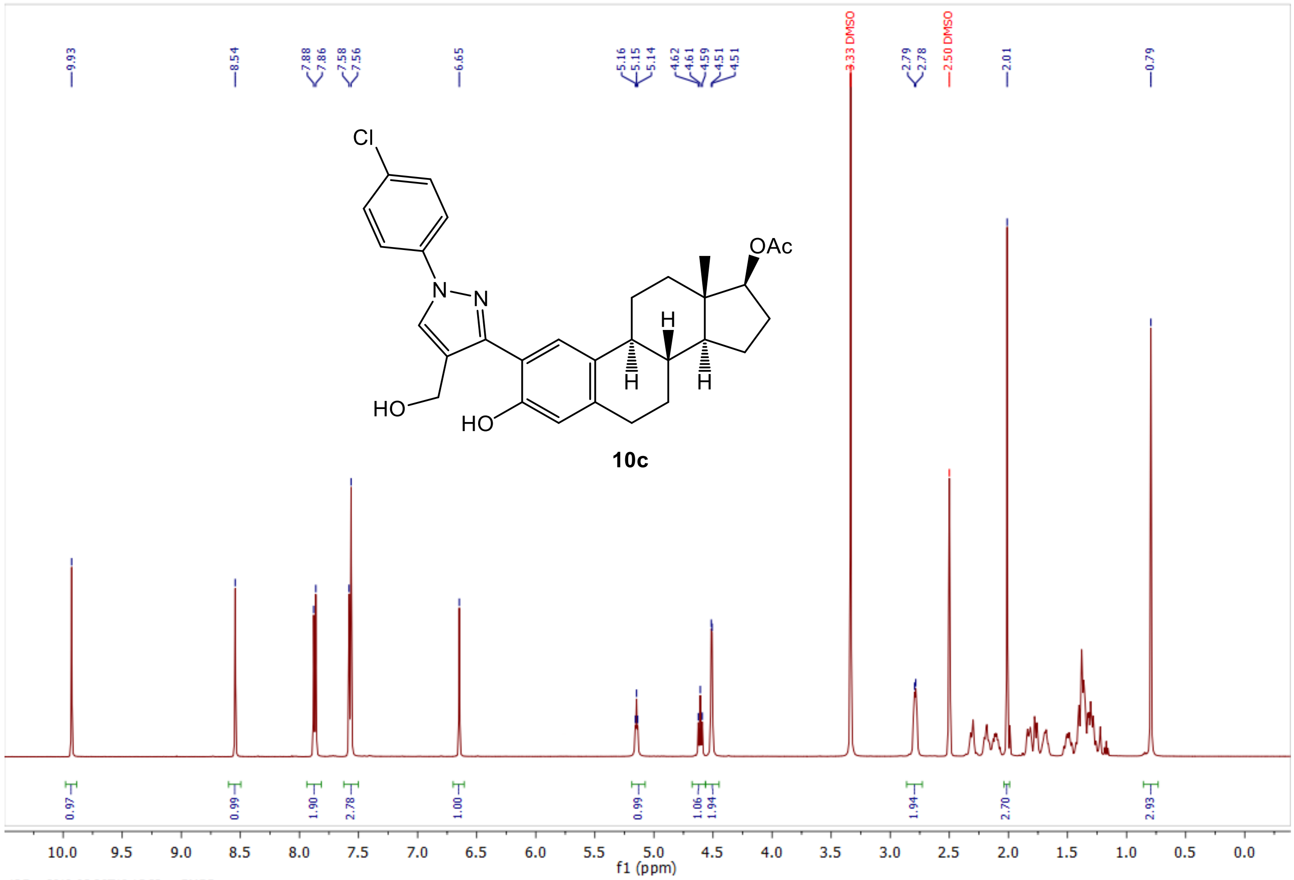

13C — 2019-03-29T10:15:33 — DMSO

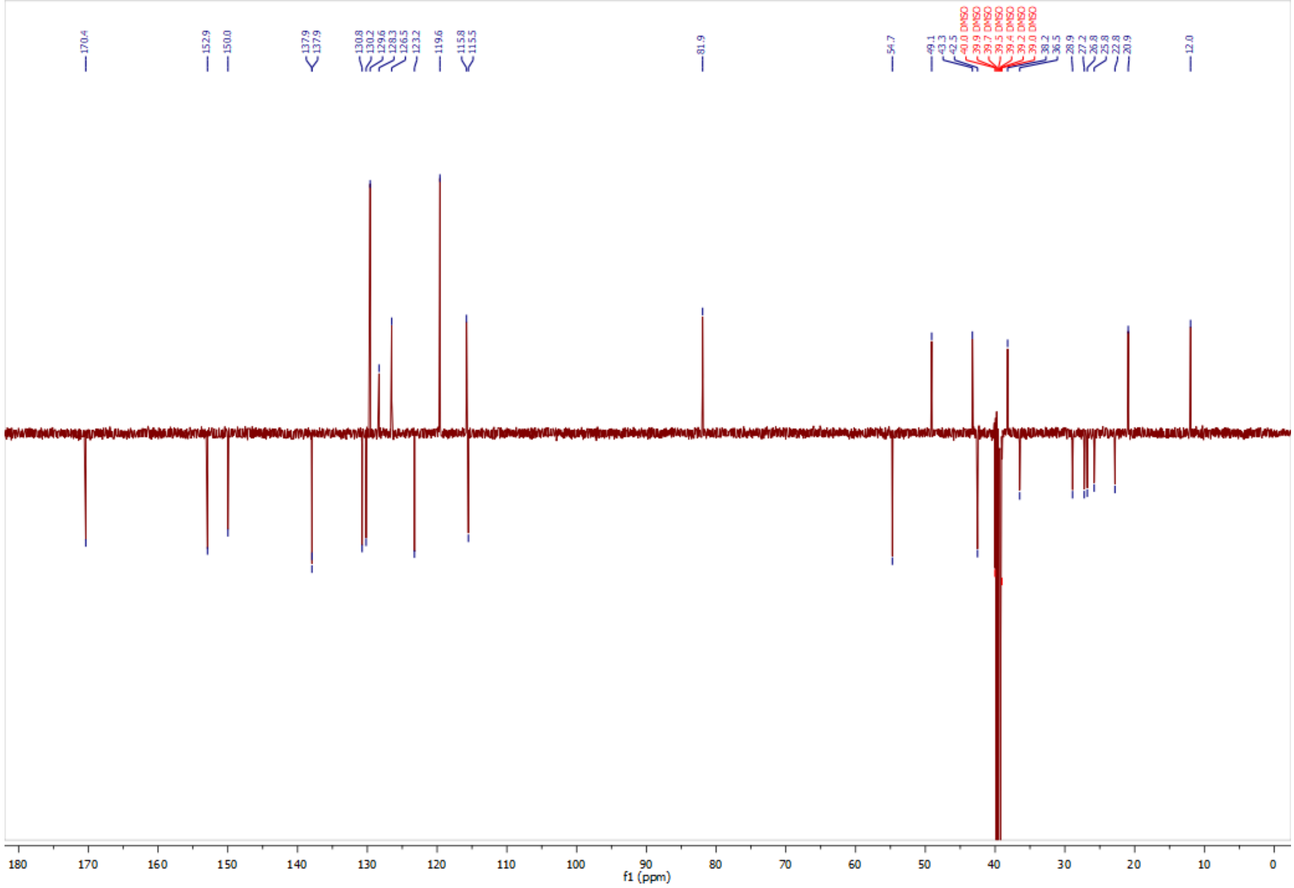

1H — 2020-01-18T05:57:11 — DMSO

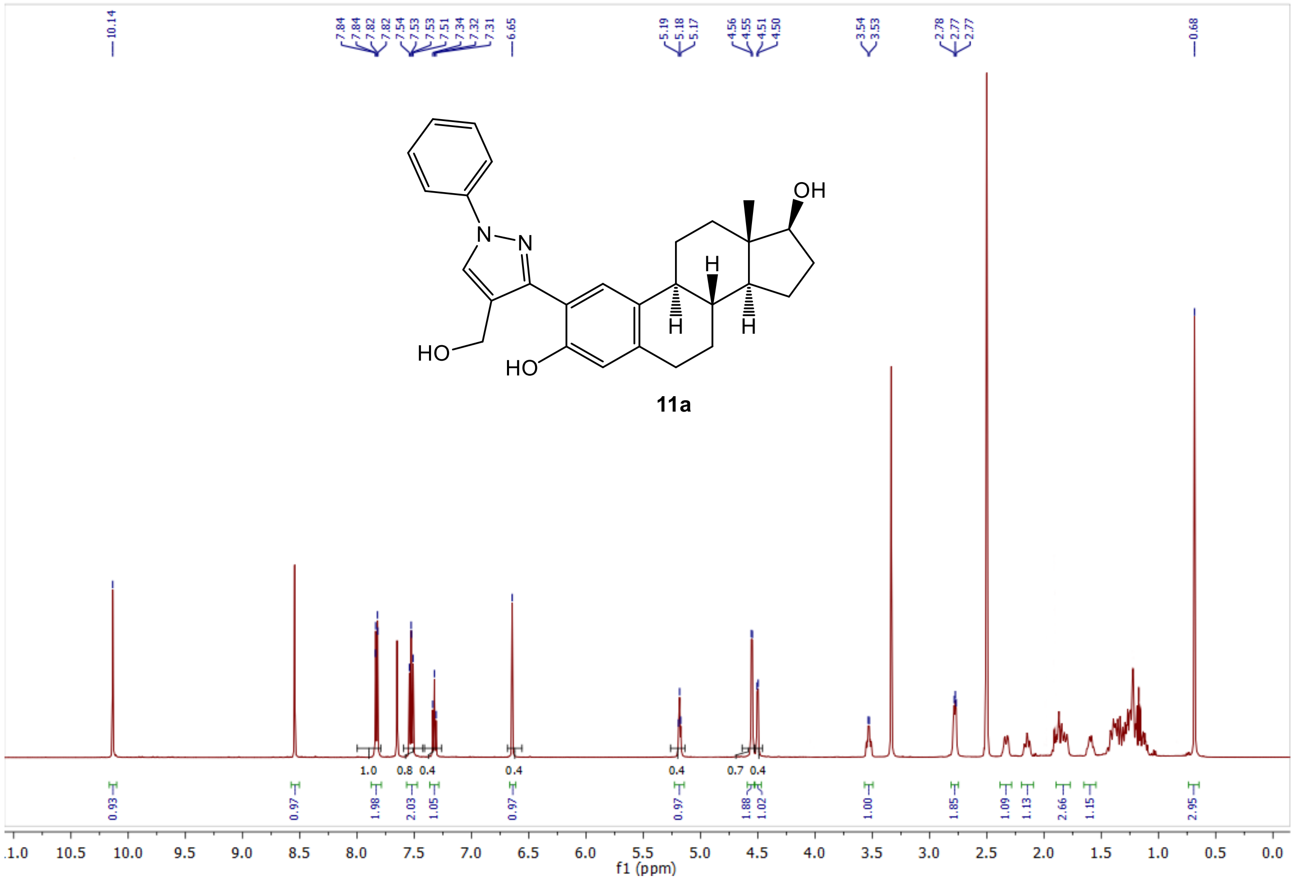

13C — 2020-01-18T06:10:54 — DMSO

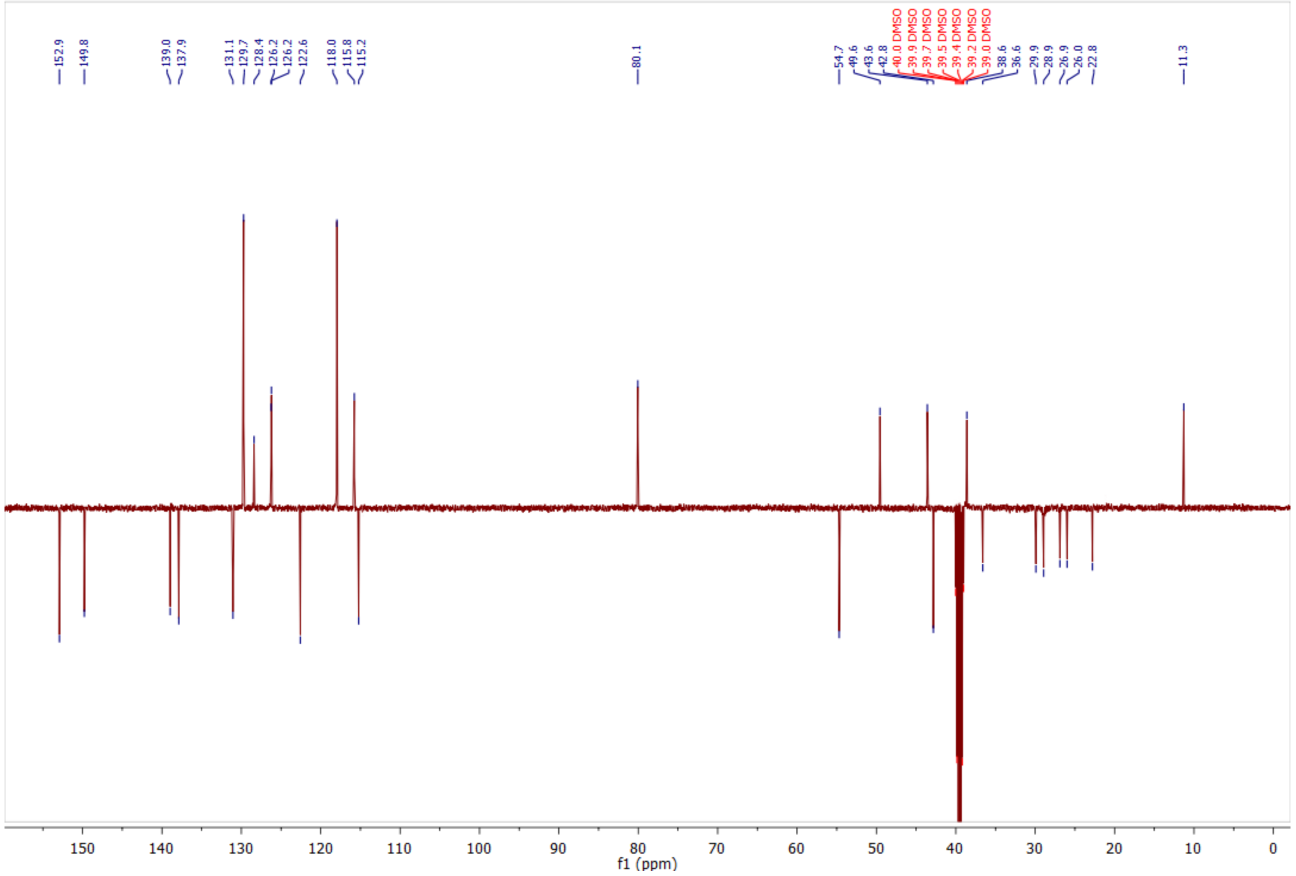

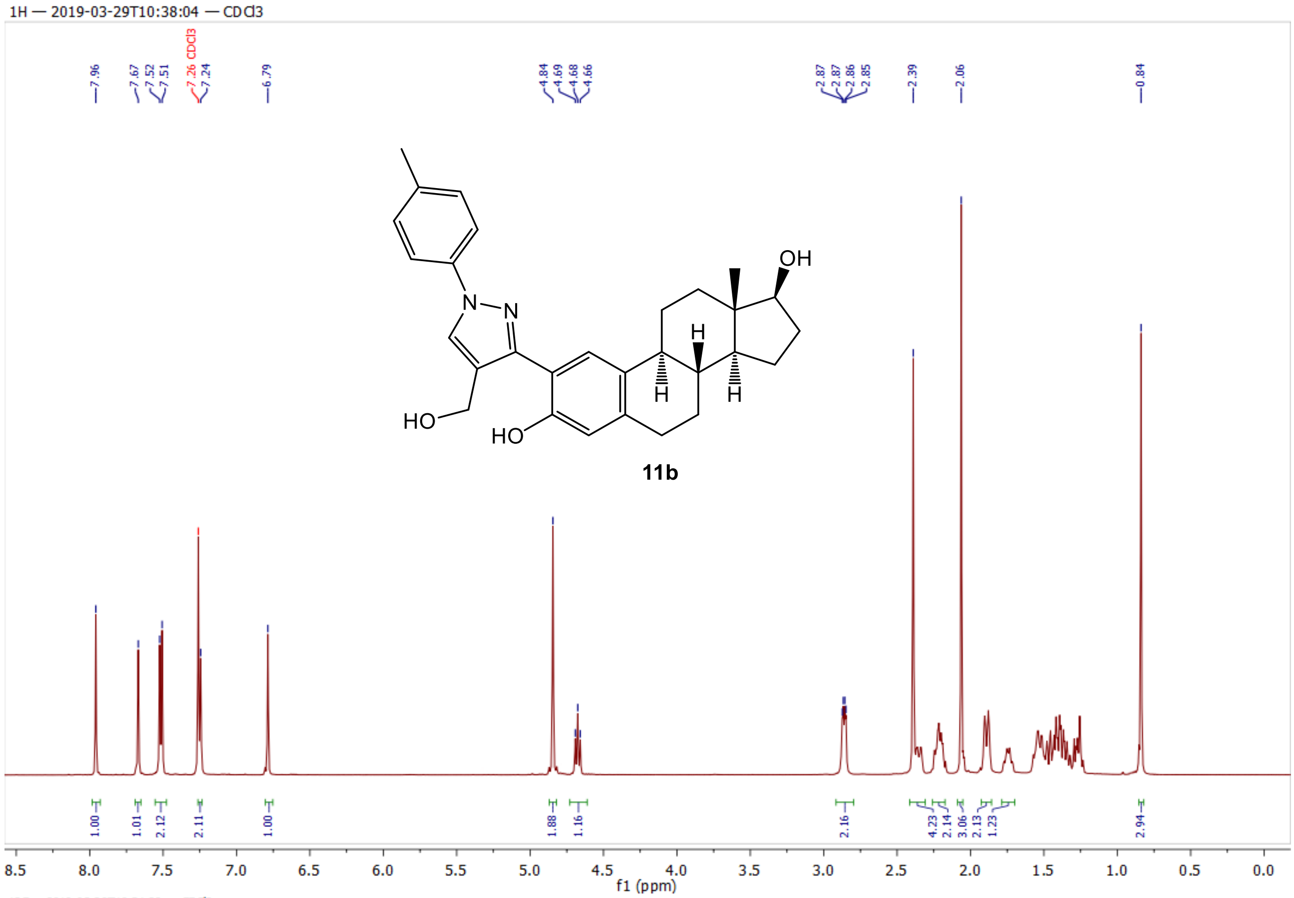
1H — 2019-03-29T10:38:04 — CDCl3
7.96
7.67
7.52
7.51
7.26 CDCl3
7.24
6.79
4.84
4.69
4.68
4.66
2.87
2.87
2.86
2.85
2.39
2.06
0.84
N
N
OH
H
H
H
HO
HO
11b
1.00
1.01
2.12
2.11
1.00
1.88
1.16
2.16
4.23
2.14
3.06
2.13
1.23
2.94
8.5 8.0 7.5 7.0 6.5 6.0 5.5 5.0 4.5 4.0 3.5 3.0 2.5 2.0 1.5 1.0 0.5 0.0
f1 (ppm)

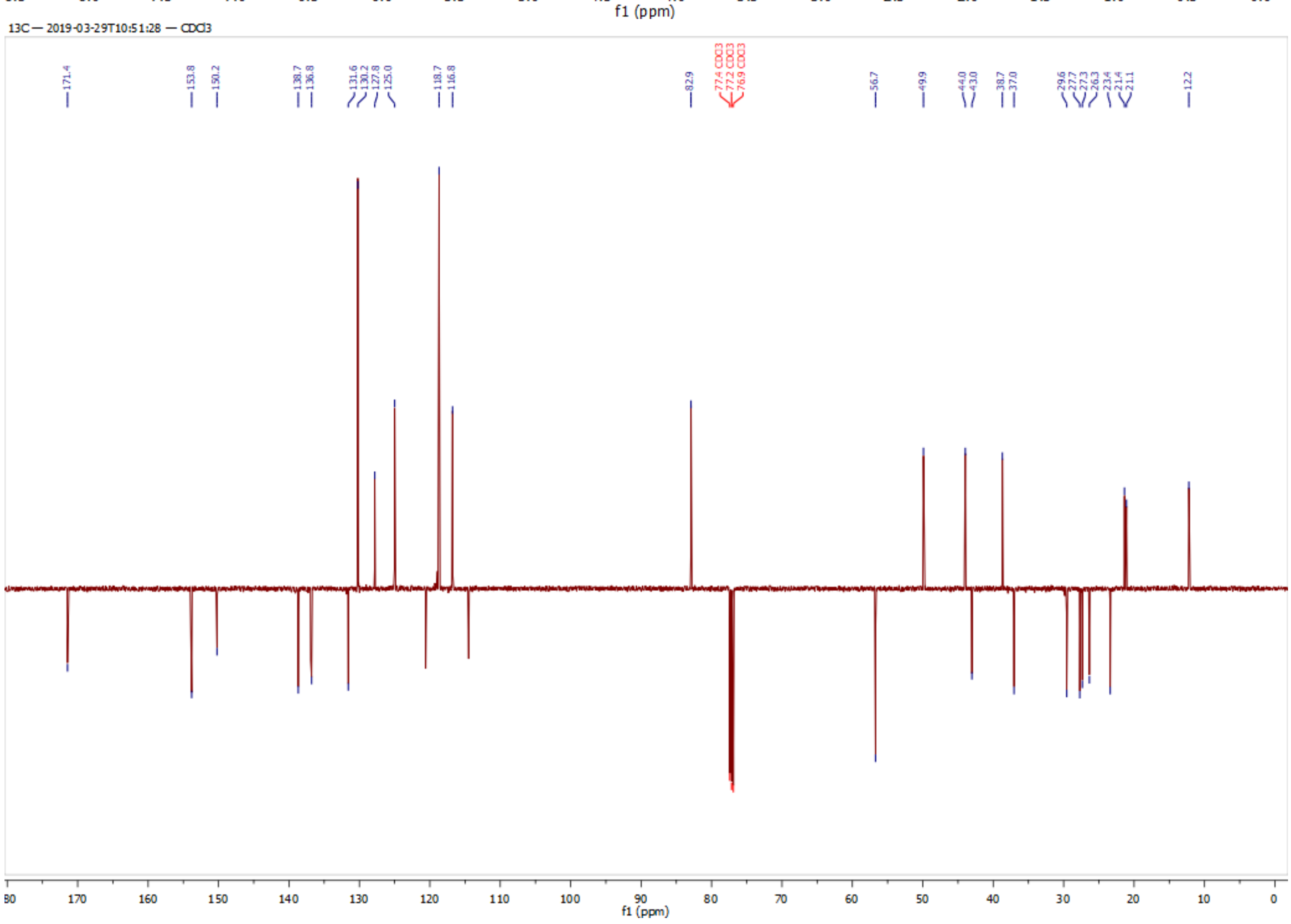
13C — 2019-03-29T10:51:28 — CDCl3
171.4
153.8
150.2
138.7
136.8
131.6
130.2
127.8
125.0
118.7
116.8
82.9
77.4 CDCl3
77.2 CDCl3
76.9 CDCl3
56.7
49.9
44.0
43.0
38.7
37.0
29.6
27.7
27.3
26.3
23.4
21.4
21.1
12.2
80 170 160 150 140 130 120 110 100 90 80 70 60 50 40 30 20 10 0
f1 (ppm)

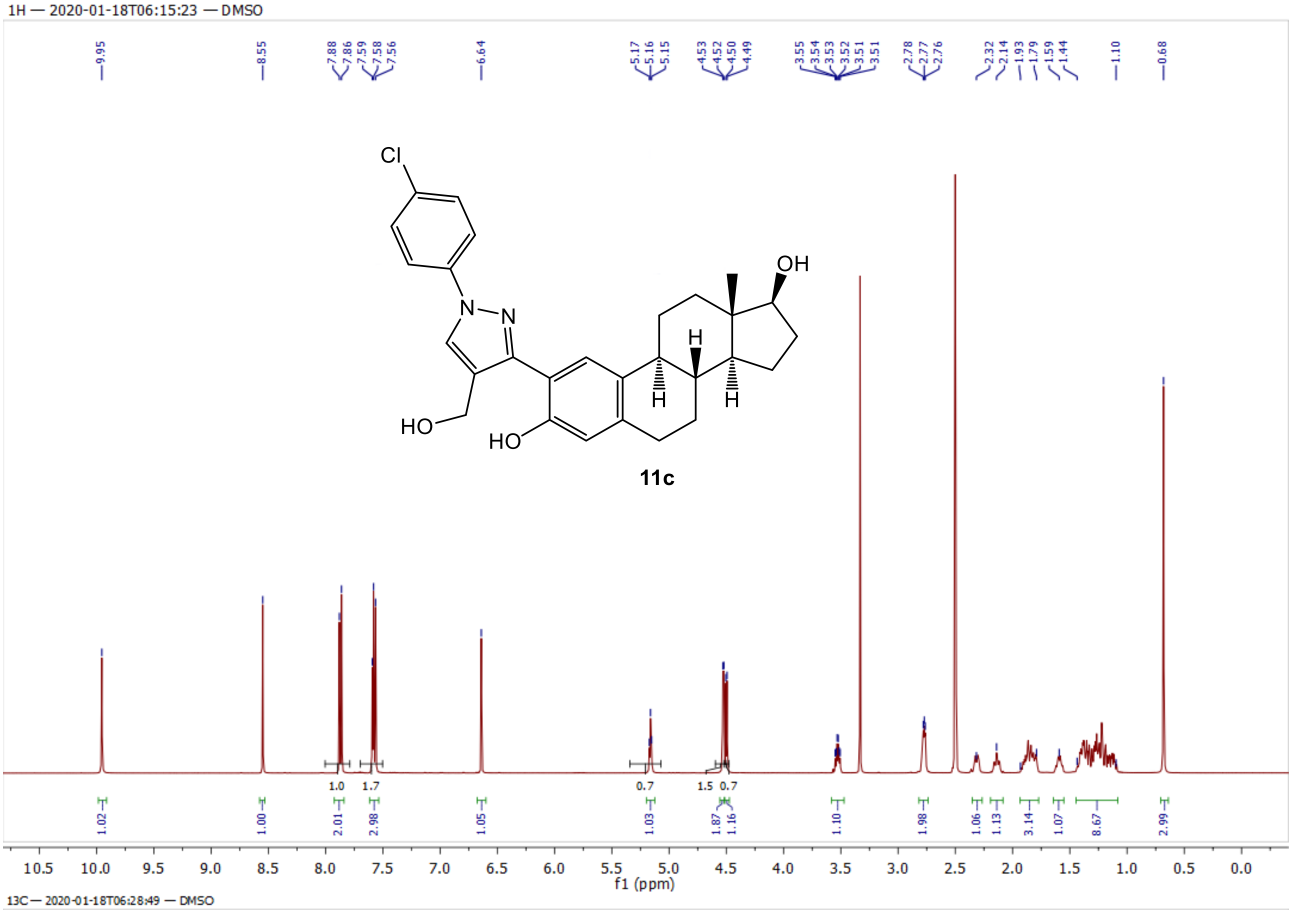
1H — 2020-01-18T06:15:23 — DMSO
9.95
8.55
7.88
7.86
7.59
7.58
7.56
6.64
5.17
5.16
5.15
4.53
4.52
4.50
4.49
3.55
3.54
3.53
3.52
3.51
3.51
2.78
2.77
2.76
2.32
2.14
1.93
1.79
1.59
1.44
1.10
0.68
Cl
N
N
OH
H
H
H
HO
HO
11c
1.0
1.7
0.7
1.5
0.7
1.02
1.00
2.01
2.98
1.05
1.03
1.87
1.16
1.10
1.98
1.06
1.13
3.14
1.07
8.67
2.99
f1 (ppm)

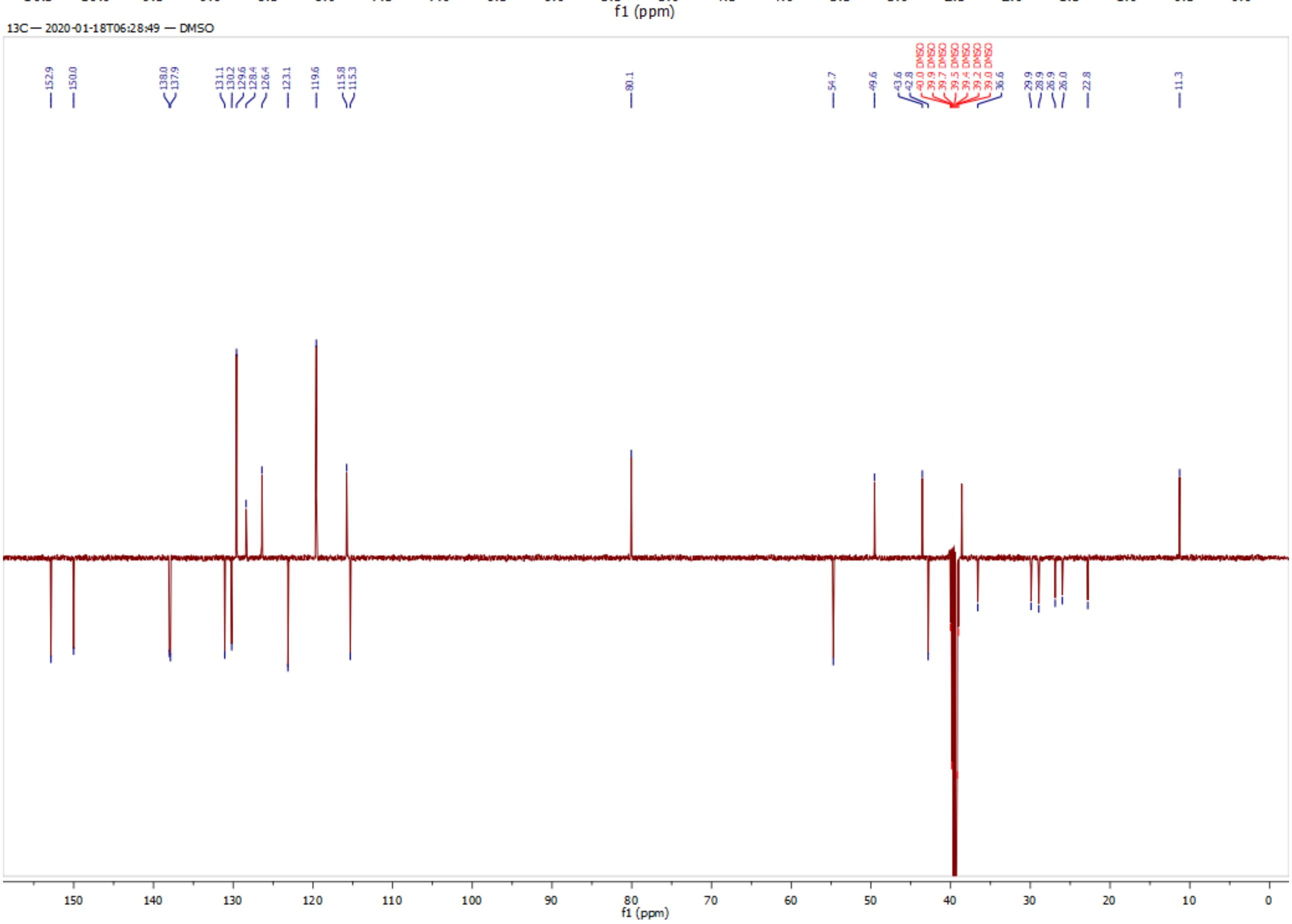
13C — 2020-01-18T06:28:49 — DMSO
152.9
150.0
138.0
137.9
131.1
130.2
129.6
128.4
126.4
123.1
119.6
115.8
115.3
80.1
54.7
49.6
43.6
42.8
DMSO
36.6
29.9
28.9
26.9
26.0
22.8
11.3
f1 (ppm)

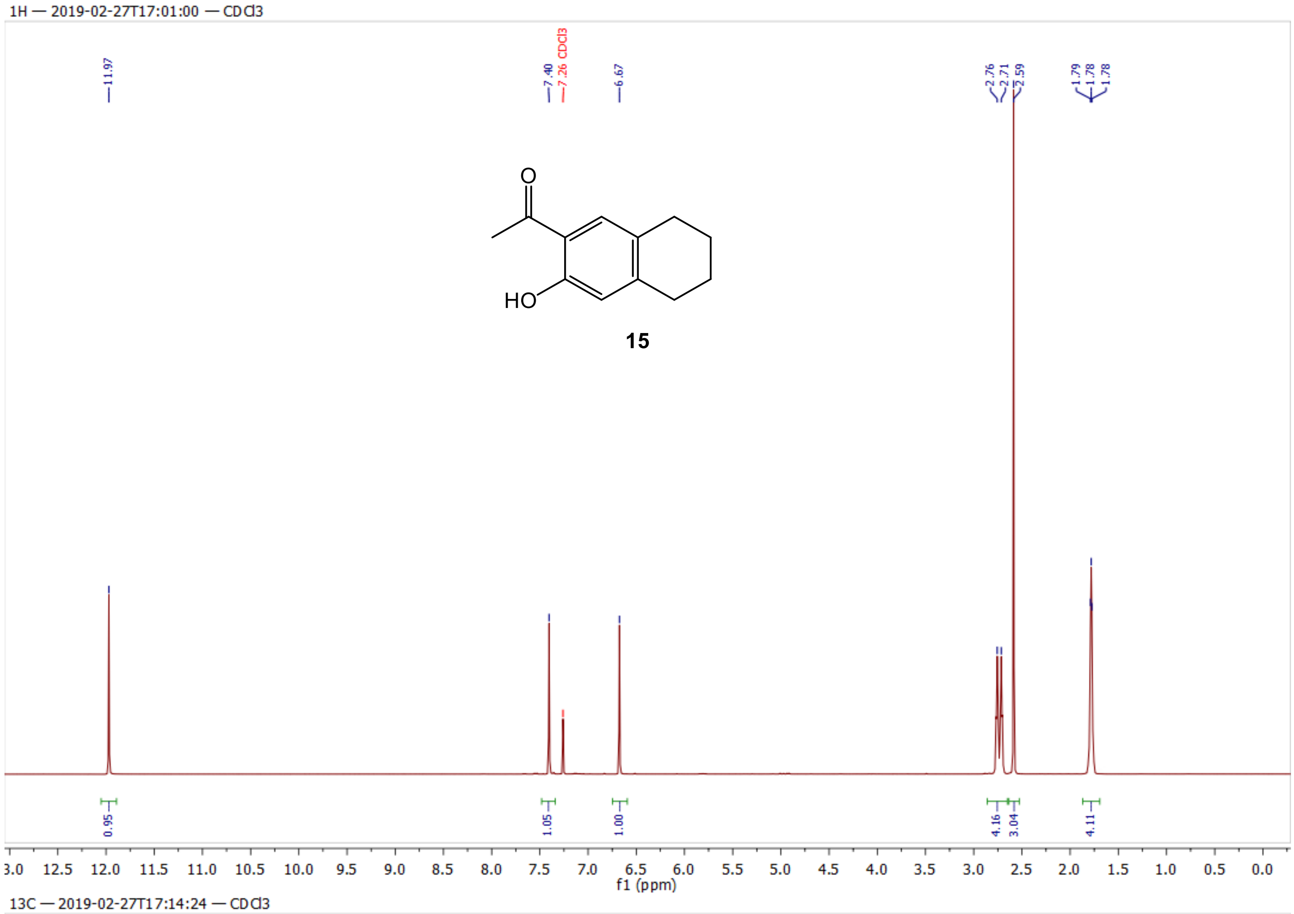
1H — 2019-02-27T17:01:00 — CDCl3
11.97
7.40
7.26 CDCl3
6.67
2.76
2.71
2.59
1.79
1.78
1.78
O
HO
15
0.95
1.05
1.00
4.16
3.04
4.11
f1 (ppm)

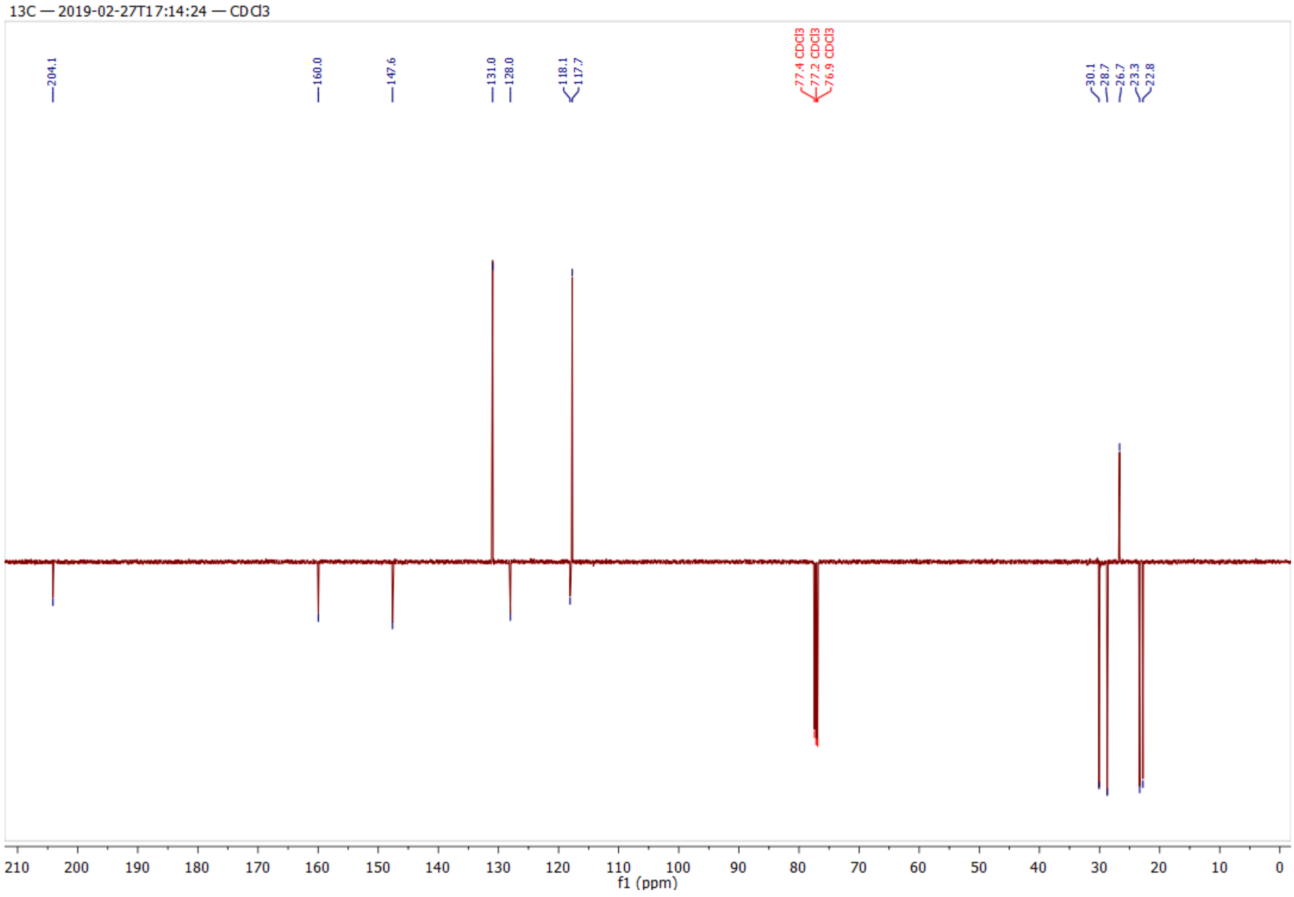
13C — 2019-02-27T17:14:24 — CDCl3
204.1
160.0
147.6
131.0
128.0
118.1
117.7
77.4 CDCl3
77.2 CDCl3
76.9 CDCl3
30.1
28.7
26.7
23.3
22.8
f1 (ppm)

1H — 2019-02-28T22:45:14 — CDCl3

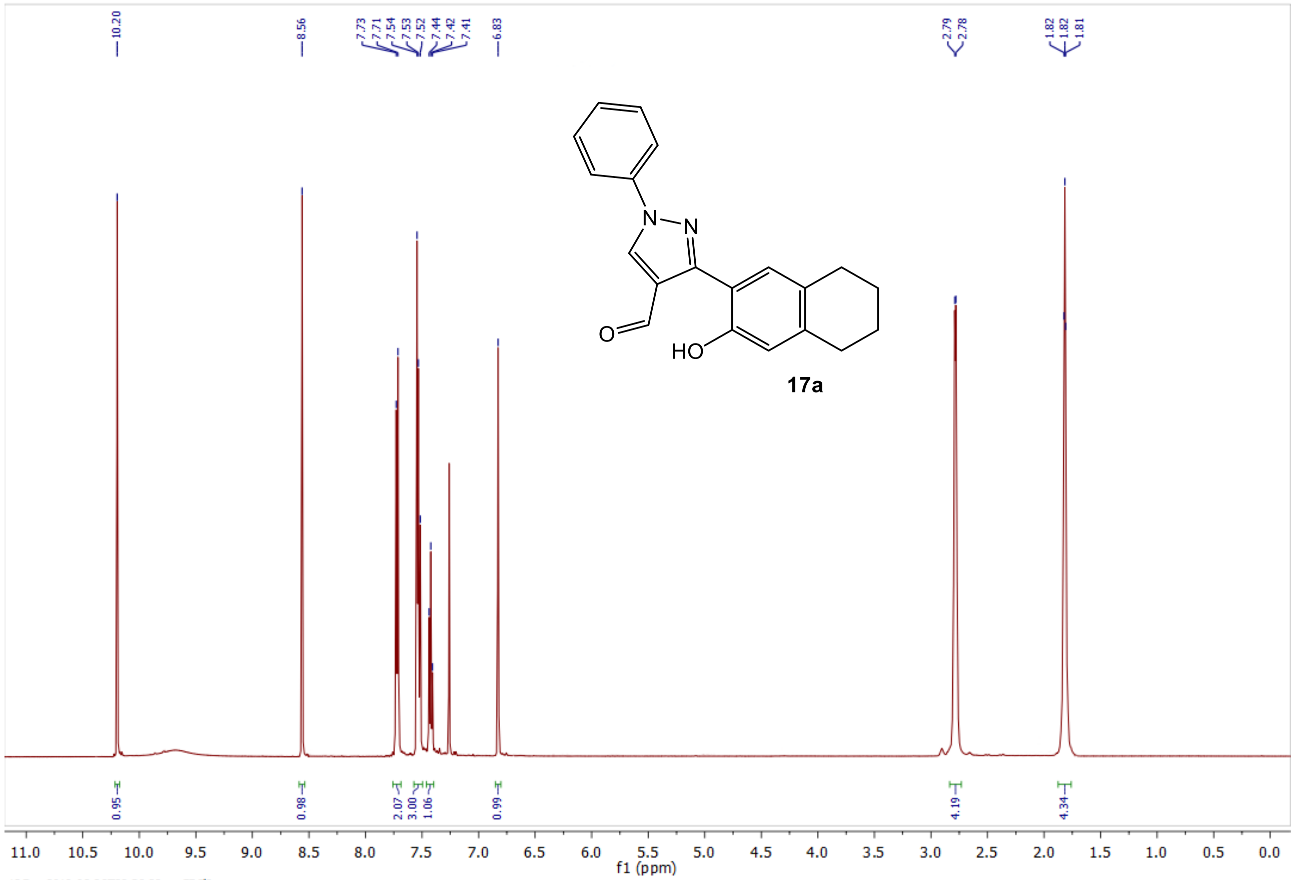

13C — 2019-02-28T22:58:38 — CDCl3

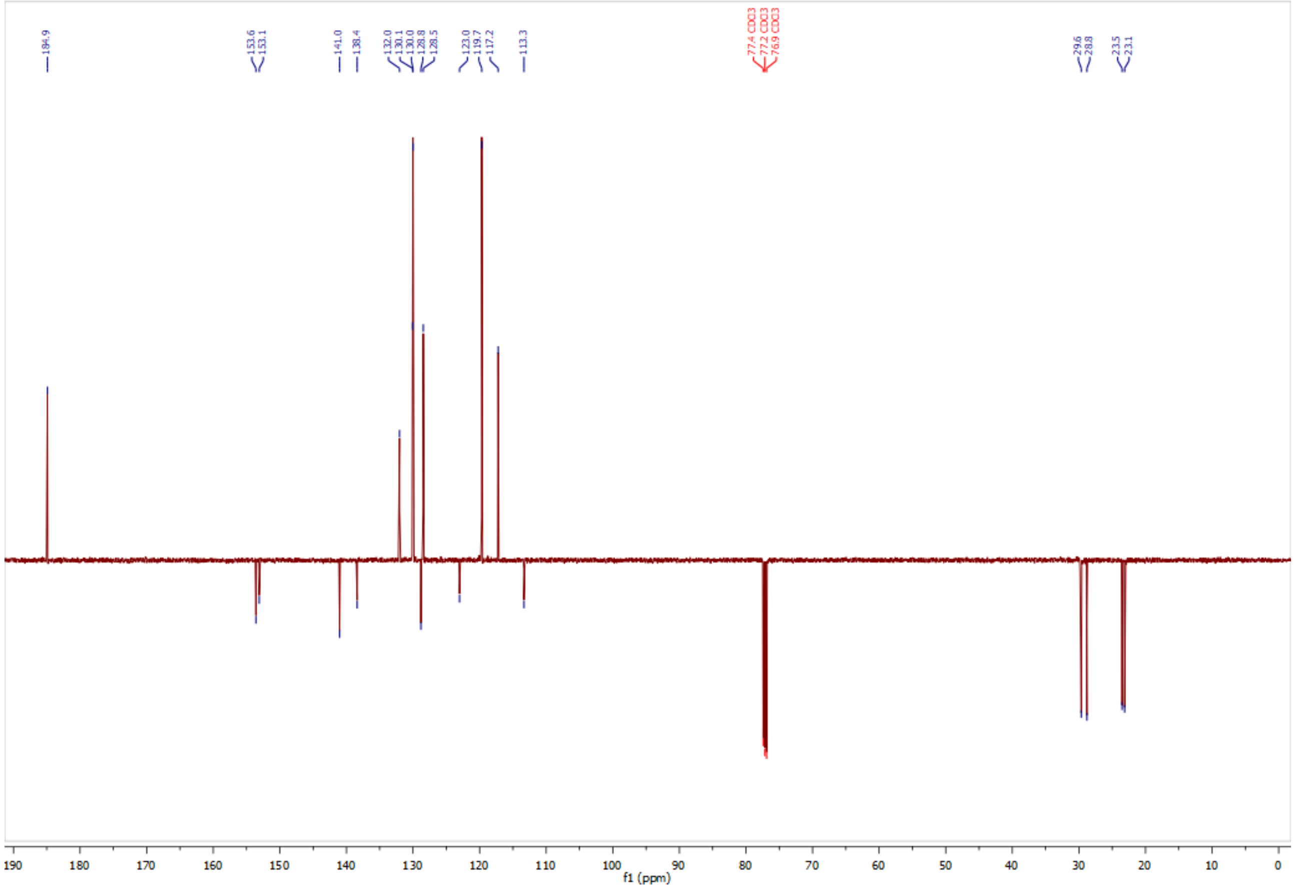

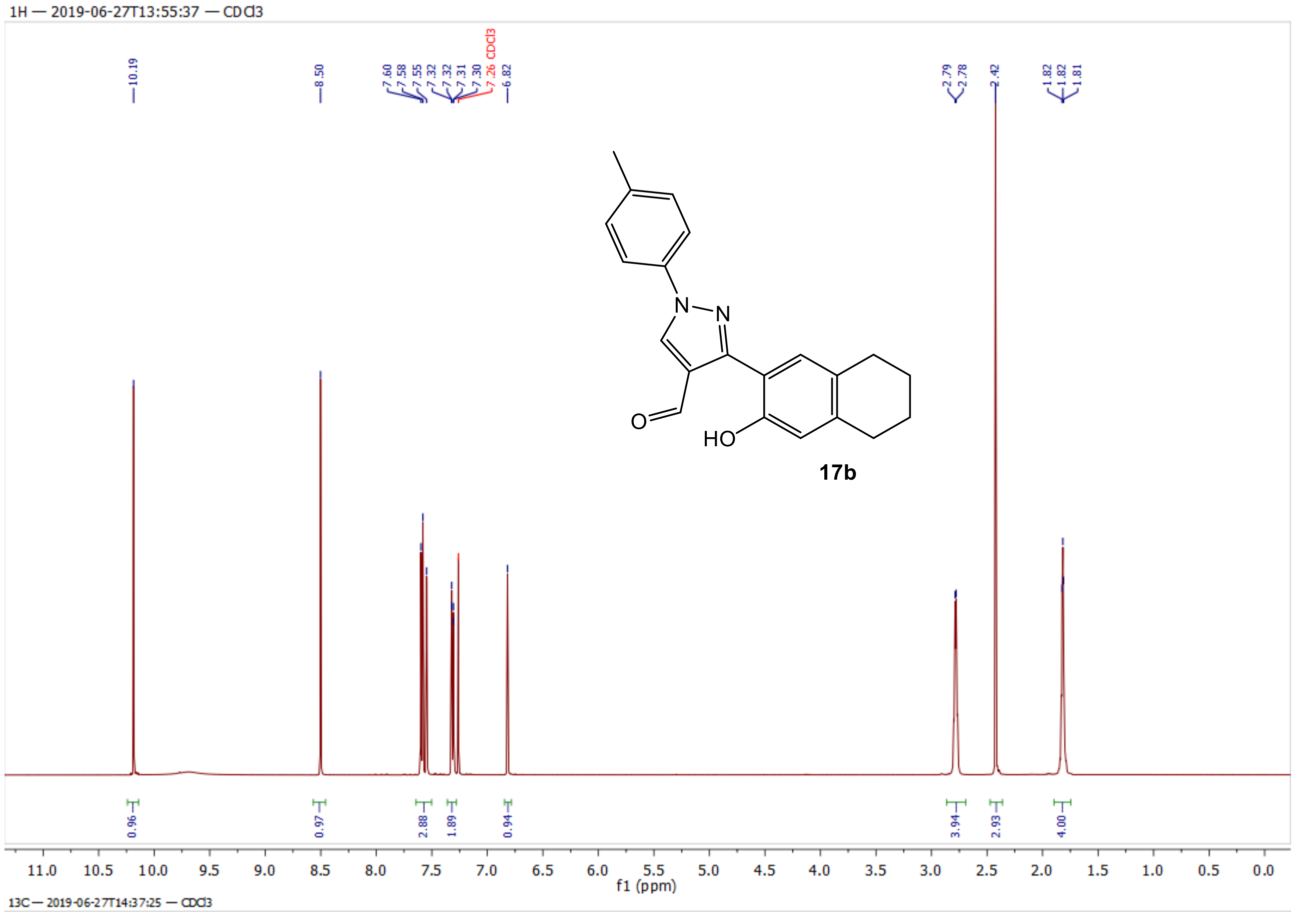
1H — 2019-06-27T13:55:37 — CDCl3
10.19
8.50
7.60
7.58
7.55
7.32
7.32
7.31
7.30
7.26 CDCl3
6.82
2.79
2.78
2.42
1.82
1.82
1.81
N
N
O
HO
17b
0.96
0.97
2.88
1.89
0.94
3.94
2.93
4.00
f1 (ppm)

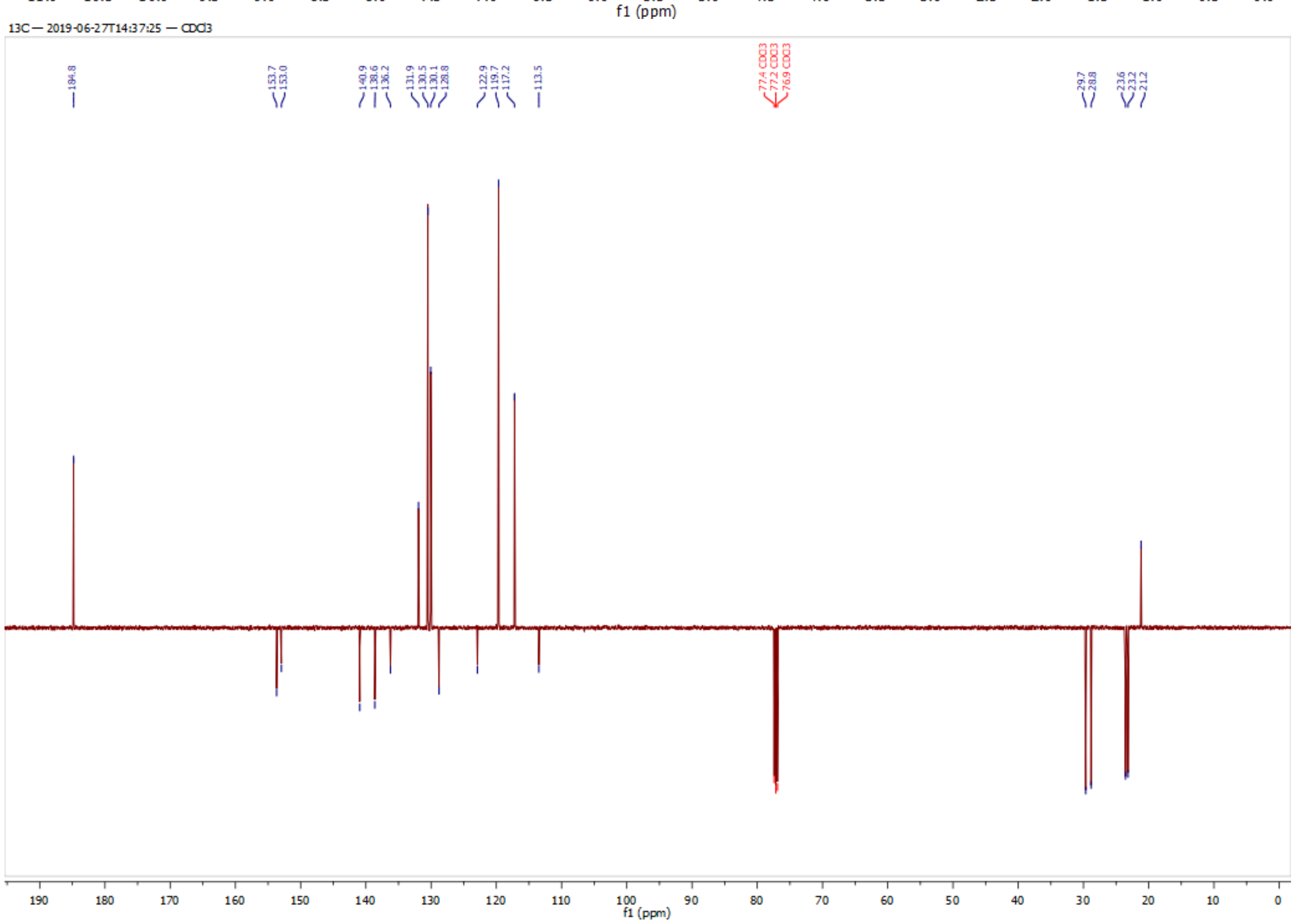
13C — 2019-06-27T14:37:25 — CDCl3
184.8
153.7
153.0
140.9
138.6
136.2
131.9
130.5
130.1
128.8
122.9
119.7
117.2
113.5
77.4 CDCl3
77.2 CDCl3
76.9 CDCl3
29.7
28.8
23.6
23.2
21.2
f1 (ppm)

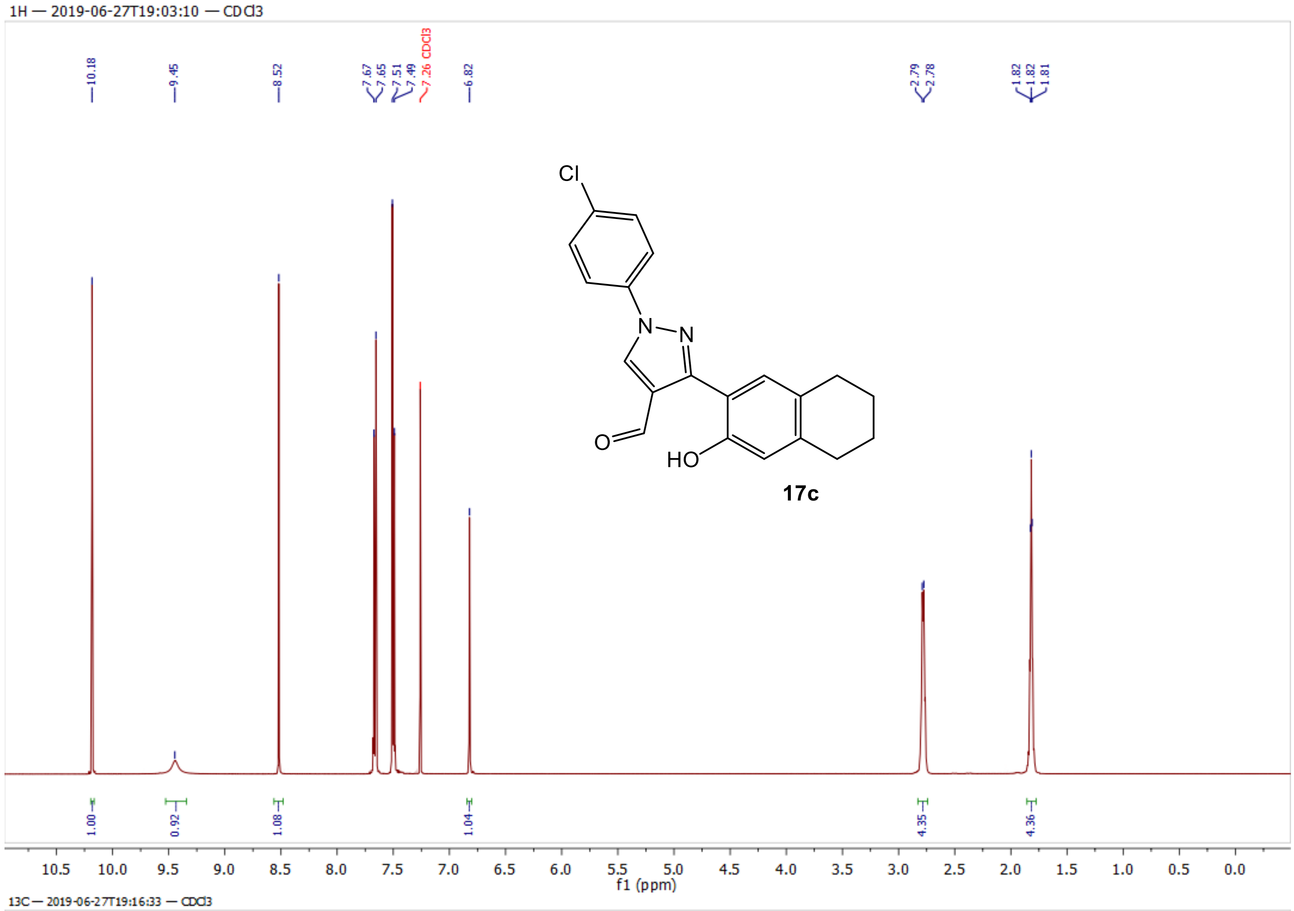
1H — 2019-06-27T19:03:10 — CDCl3
10.18
9.45
8.52
7.67
7.65
7.51
7.49
7.26 CDCl3
6.82
2.79
2.78
1.82
1.82
1.81
Cl
N
N
O
HO
17c
1.00
0.92
1.08
1.04
4.35
4.36
f1 (ppm)

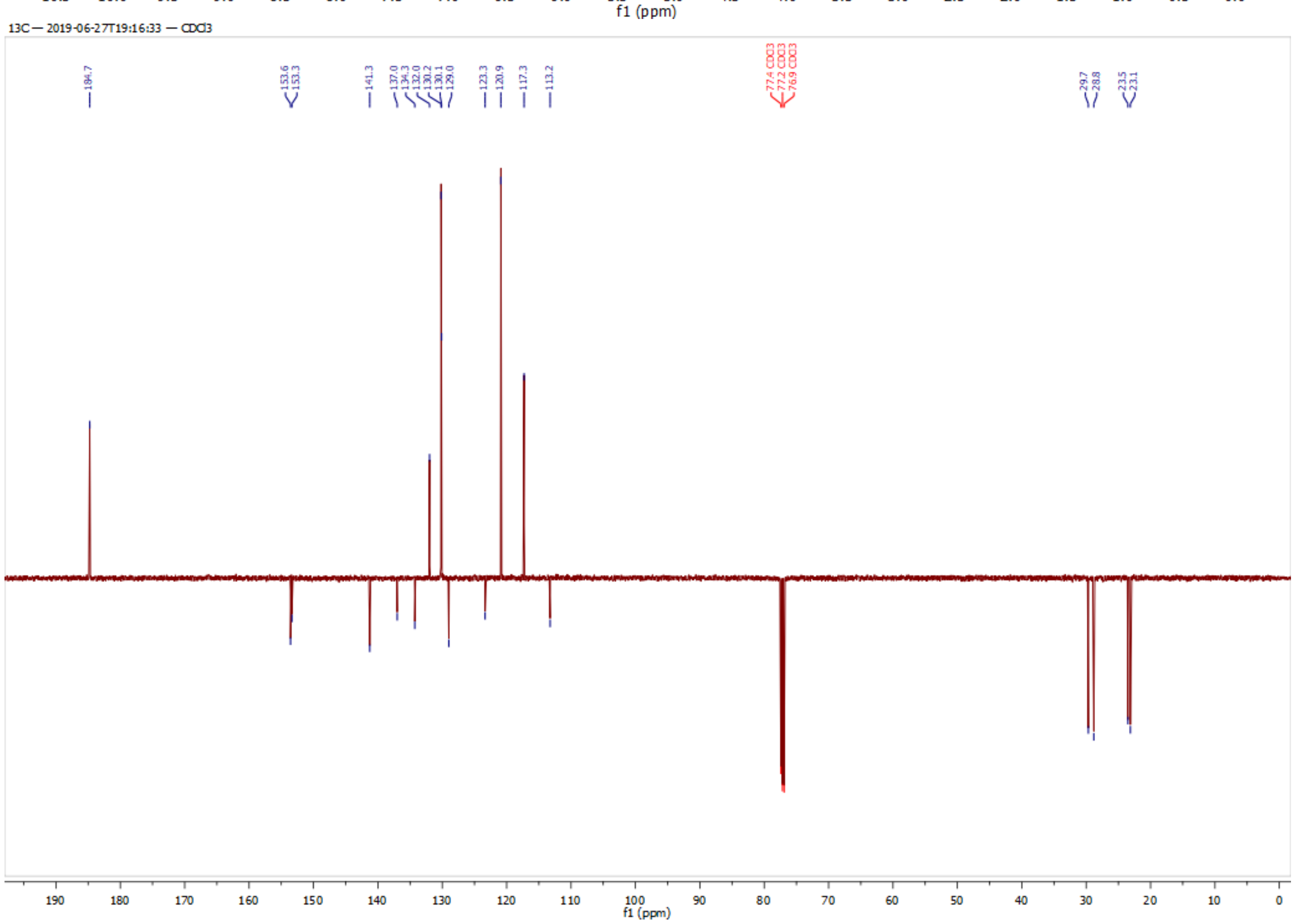
13C — 2019-06-27T19:16:33 — CDCl3
184.7
153.6
153.3
141.3
137.0
134.3
132.0
130.2
130.1
129.0
123.3
120.9
117.3
113.2
77.4 CDCl3
77.2 CDCl3
76.9 CDCl3
29.7
28.8
23.5
23.1
f1 (ppm)

1H — 2019-04-18T21:21:00 — CDCl3

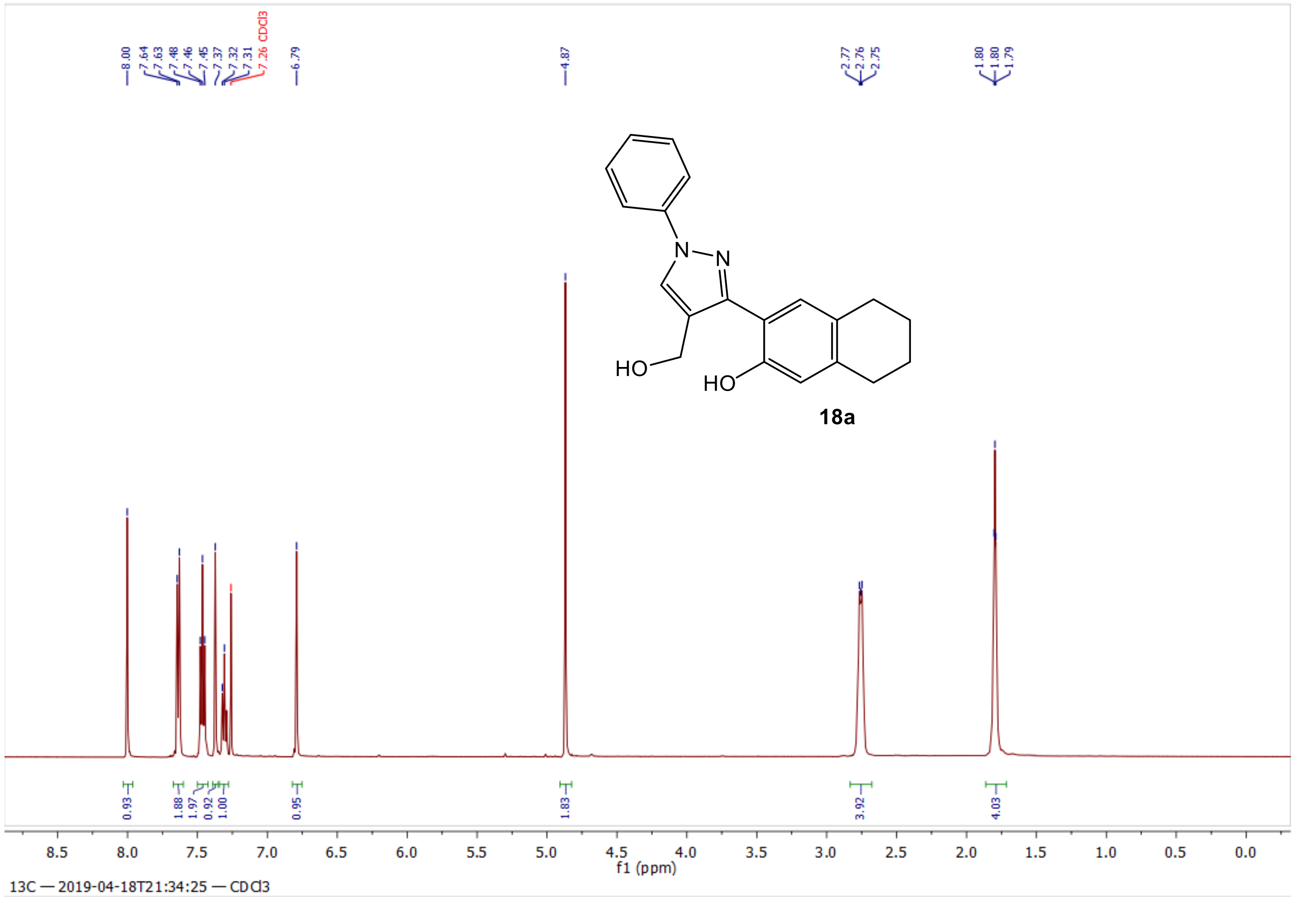

13C — 2019-04-18T21:34:25 — CDCl3

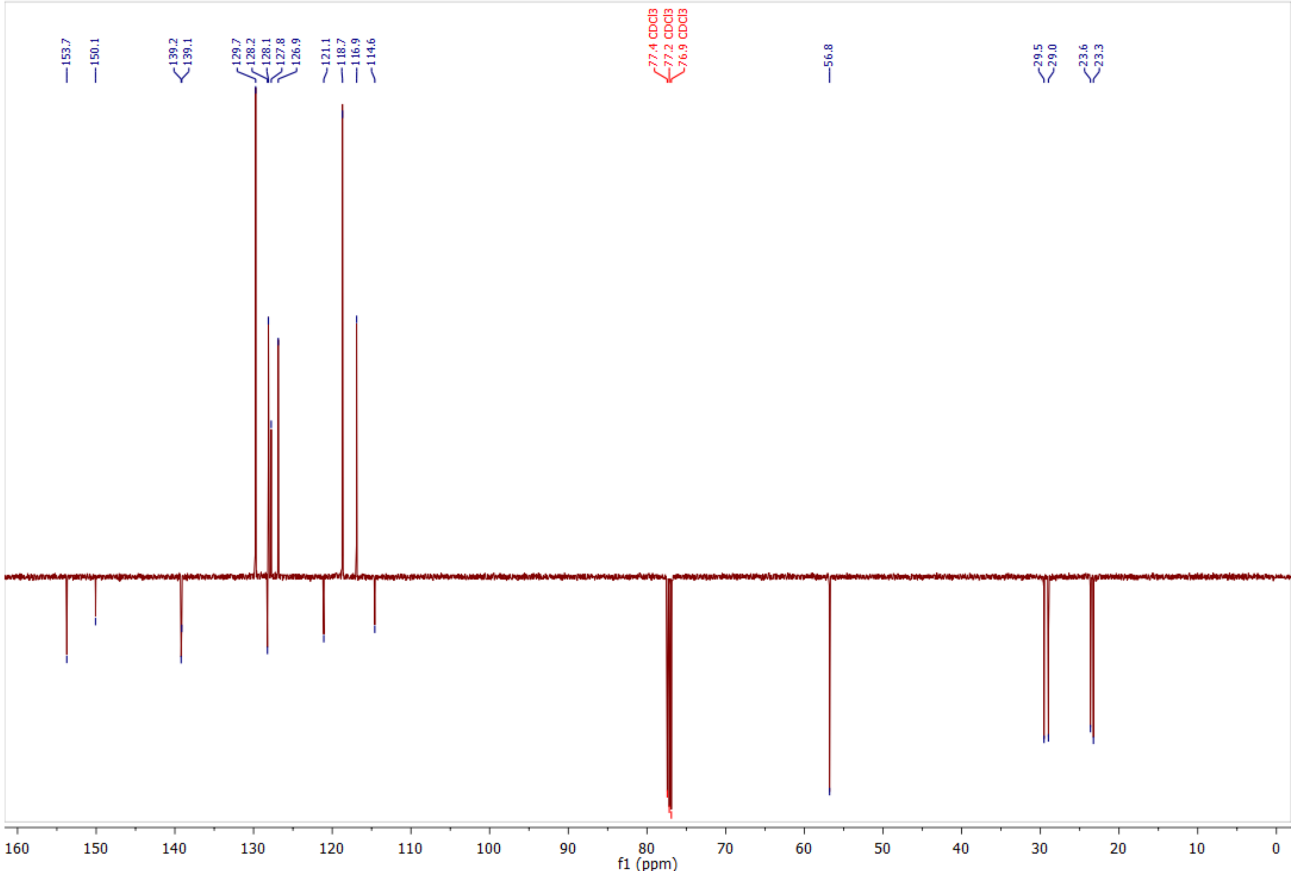

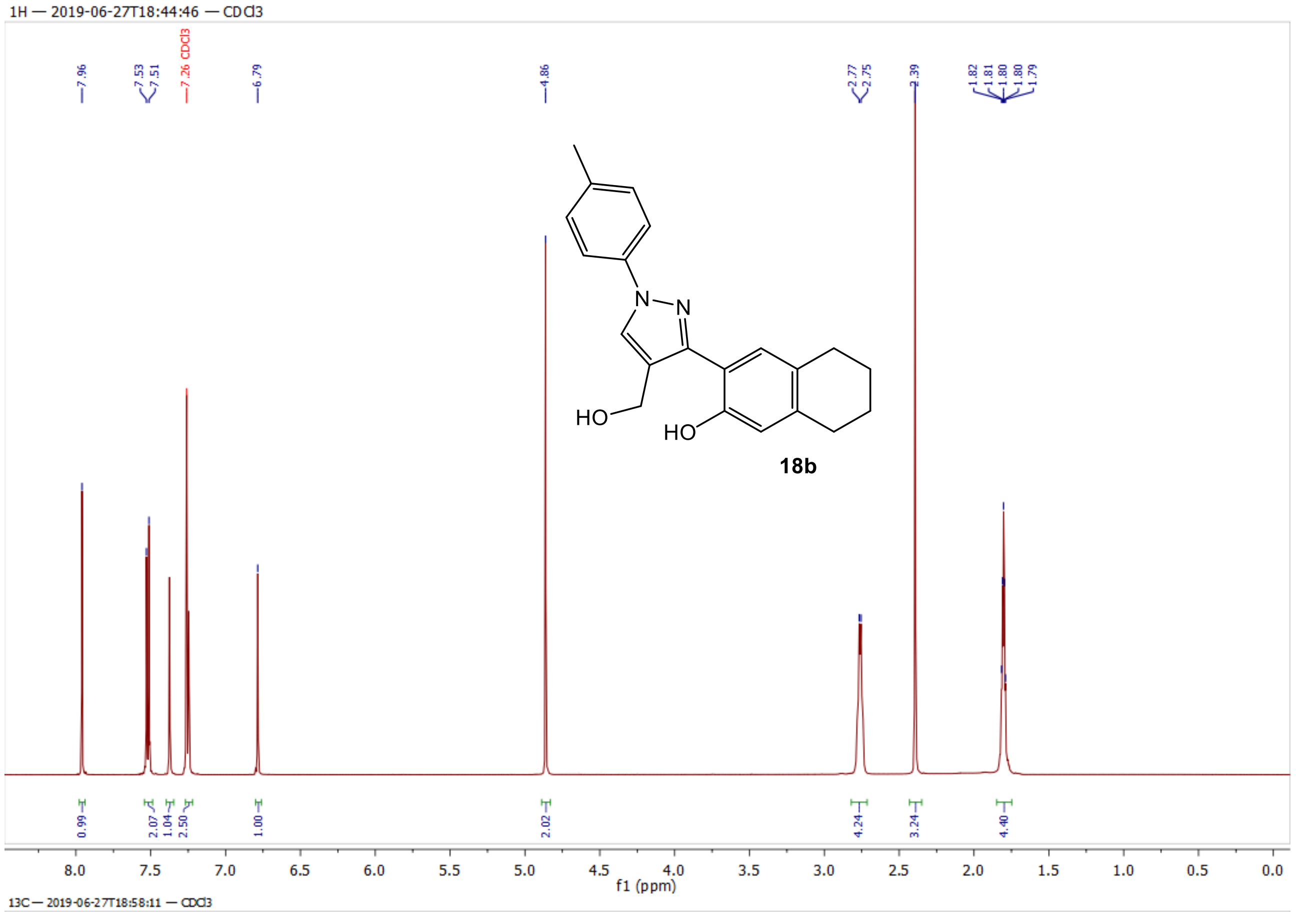
1H — 2019-06-27T18:44:46 — CDCl3
7.96
7.53
7.51
7.26 CDCl3
6.79
4.86
2.77
2.75
2.39
1.82
1.81
1.80
1.80
1.79
HO
HO
N
N
18b
0.99
2.07
1.04
2.50
1.00
2.02
4.24
3.24
4.40
f1 (ppm)

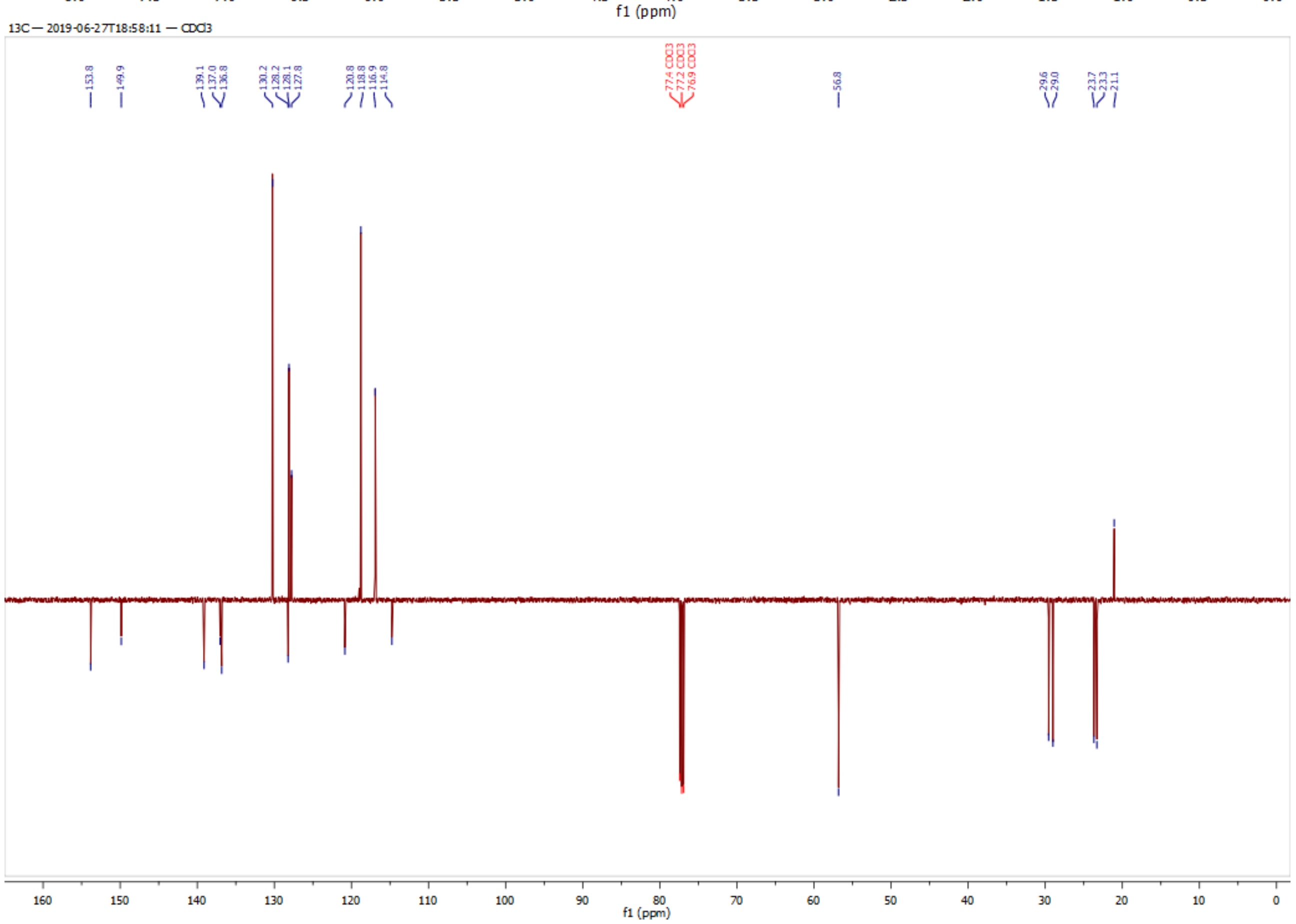
13C — 2019-06-27T18:58:11 — CDCl3
153.8
149.9
139.1
137.0
136.8
130.2
128.2
128.1
127.8
120.8
118.8
116.9
114.8
77.4 CDCl3
77.2 CDCl3
76.9 CDCl3
56.8
29.6
29.0
23.7
23.3
21.1
f1 (ppm)

1H — 2019-06-27T19:38:38 — CDCl3

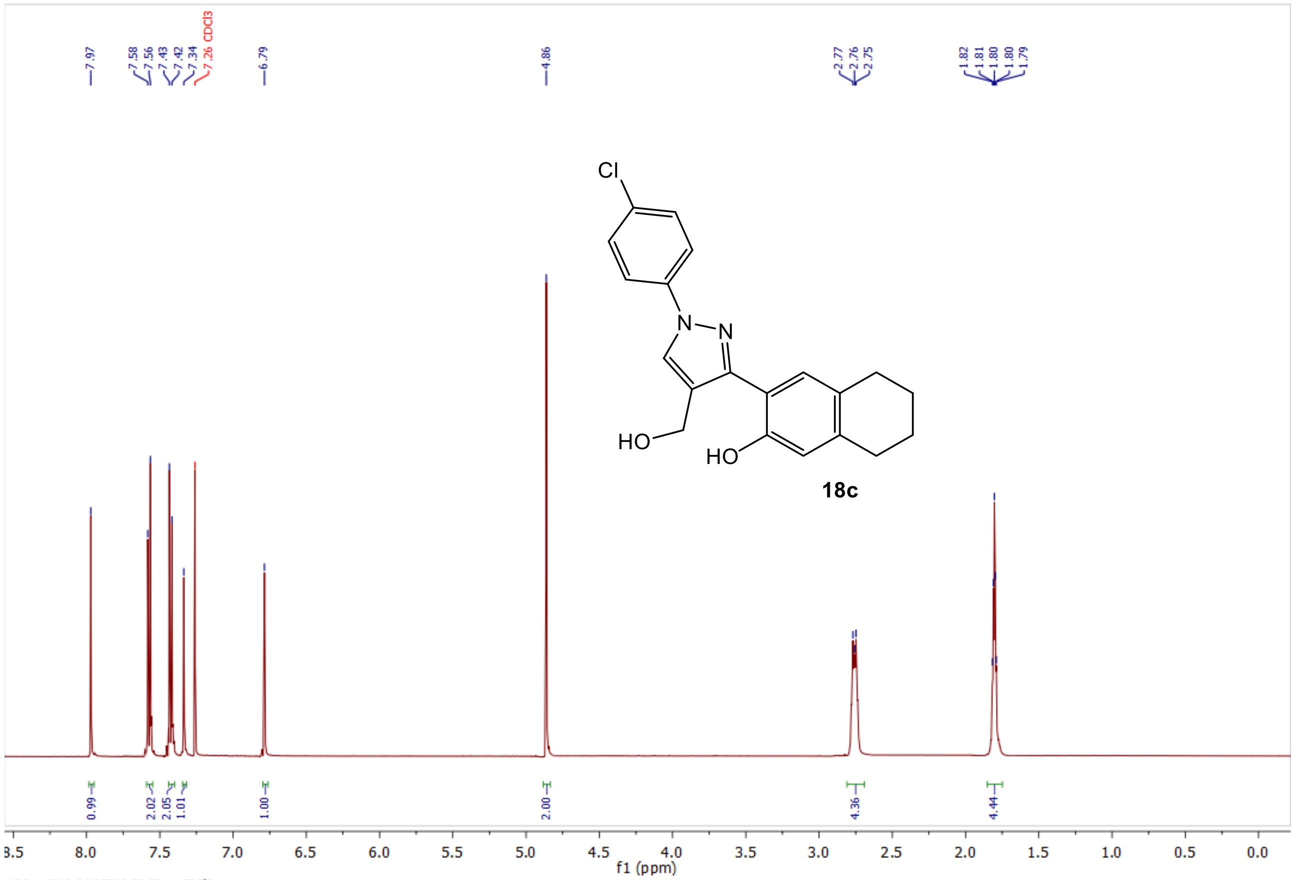

13C — 2019-06-27T19:52:03 — CDCl3

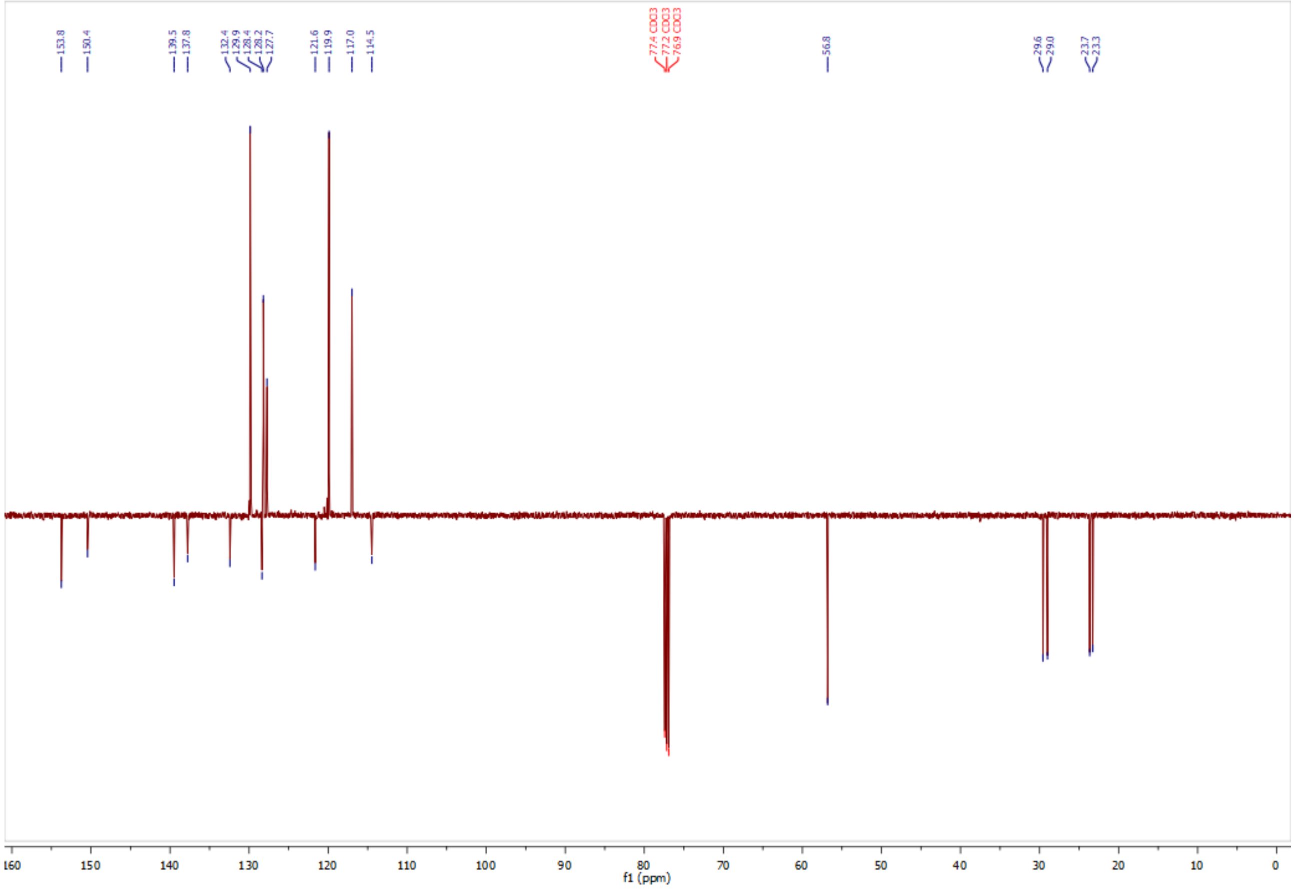

1H — 2019-04-18T21:03:39 — CDCl3

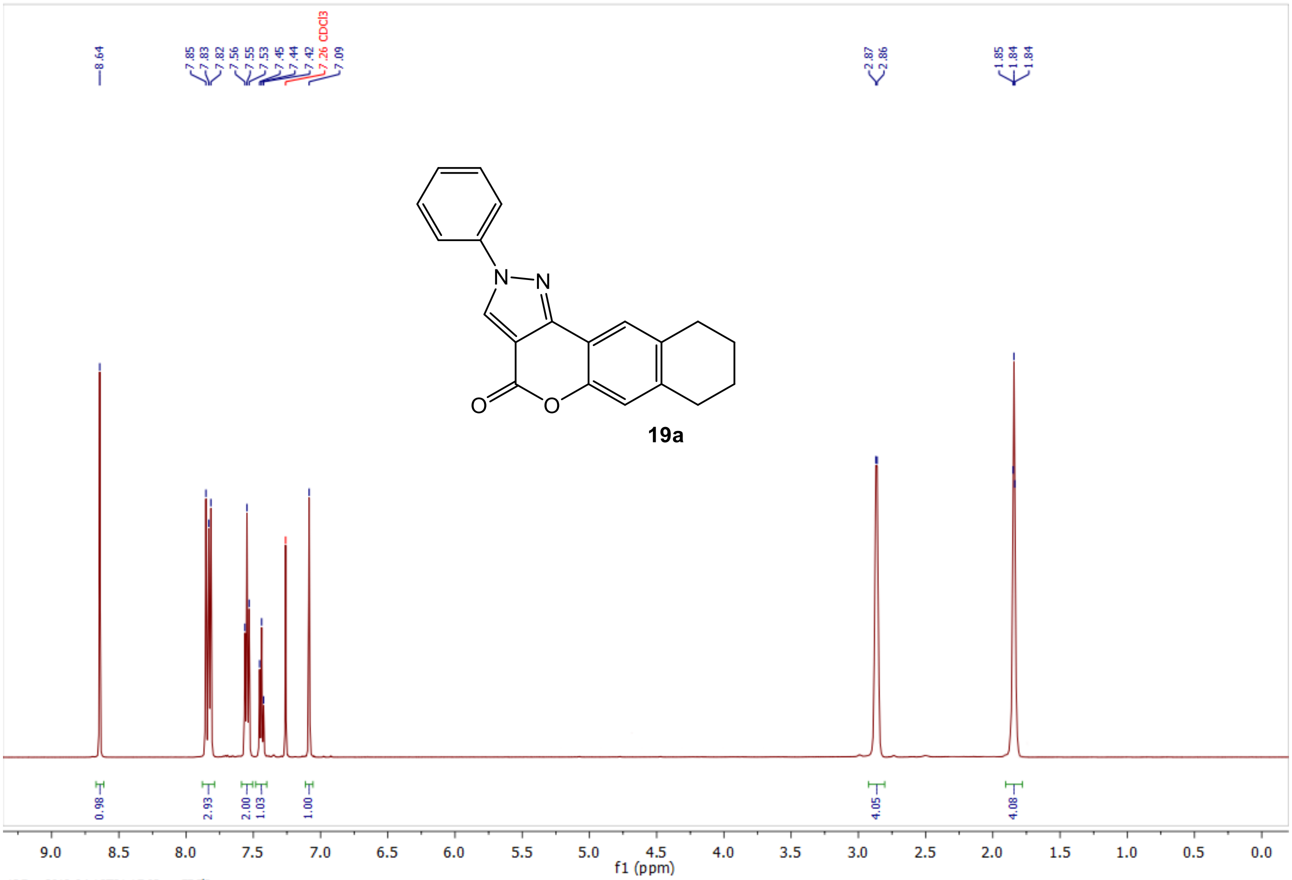

13C — 2019-04-18T21:17:03 — CDCl3

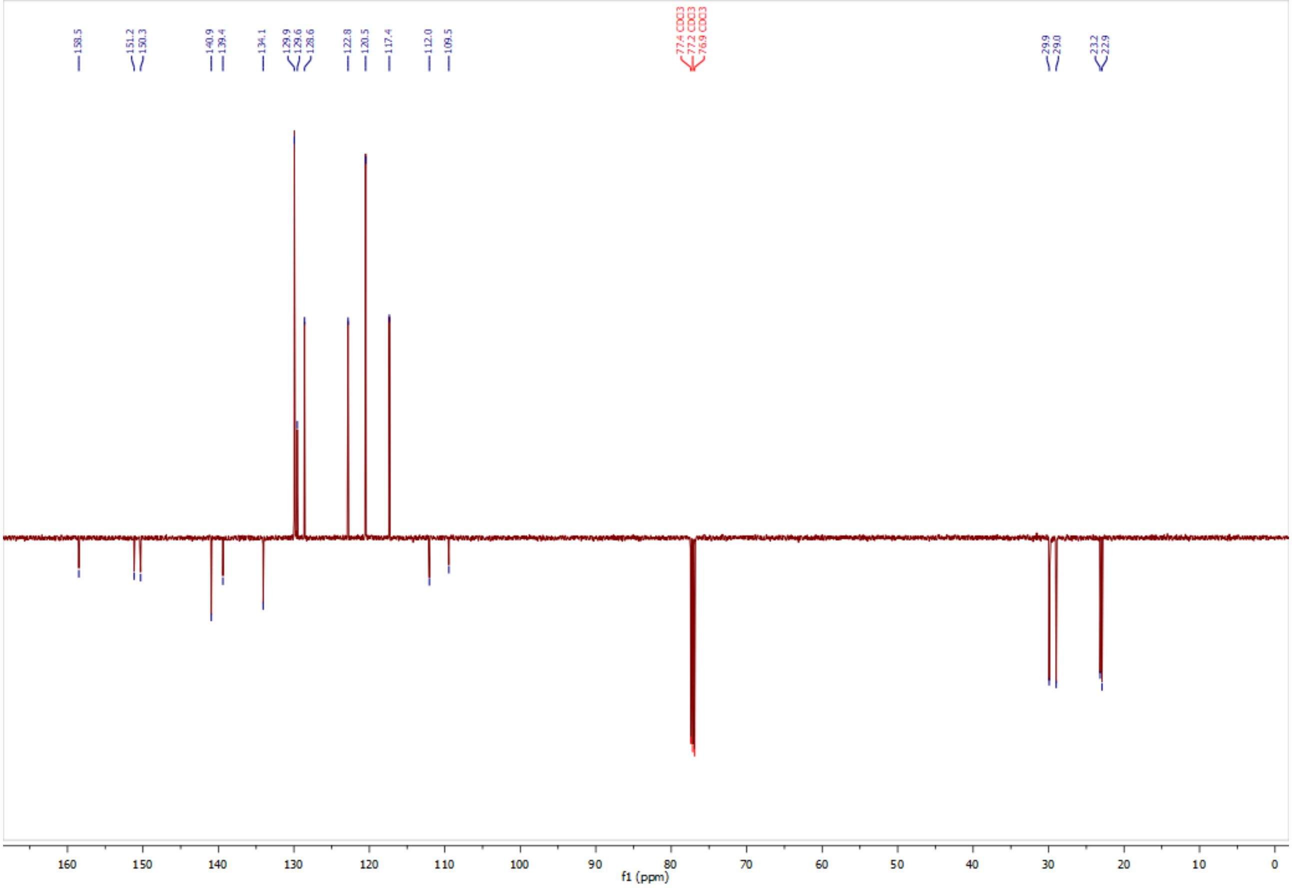

1H — 2019-06-27T18:24:57 — CDCl3

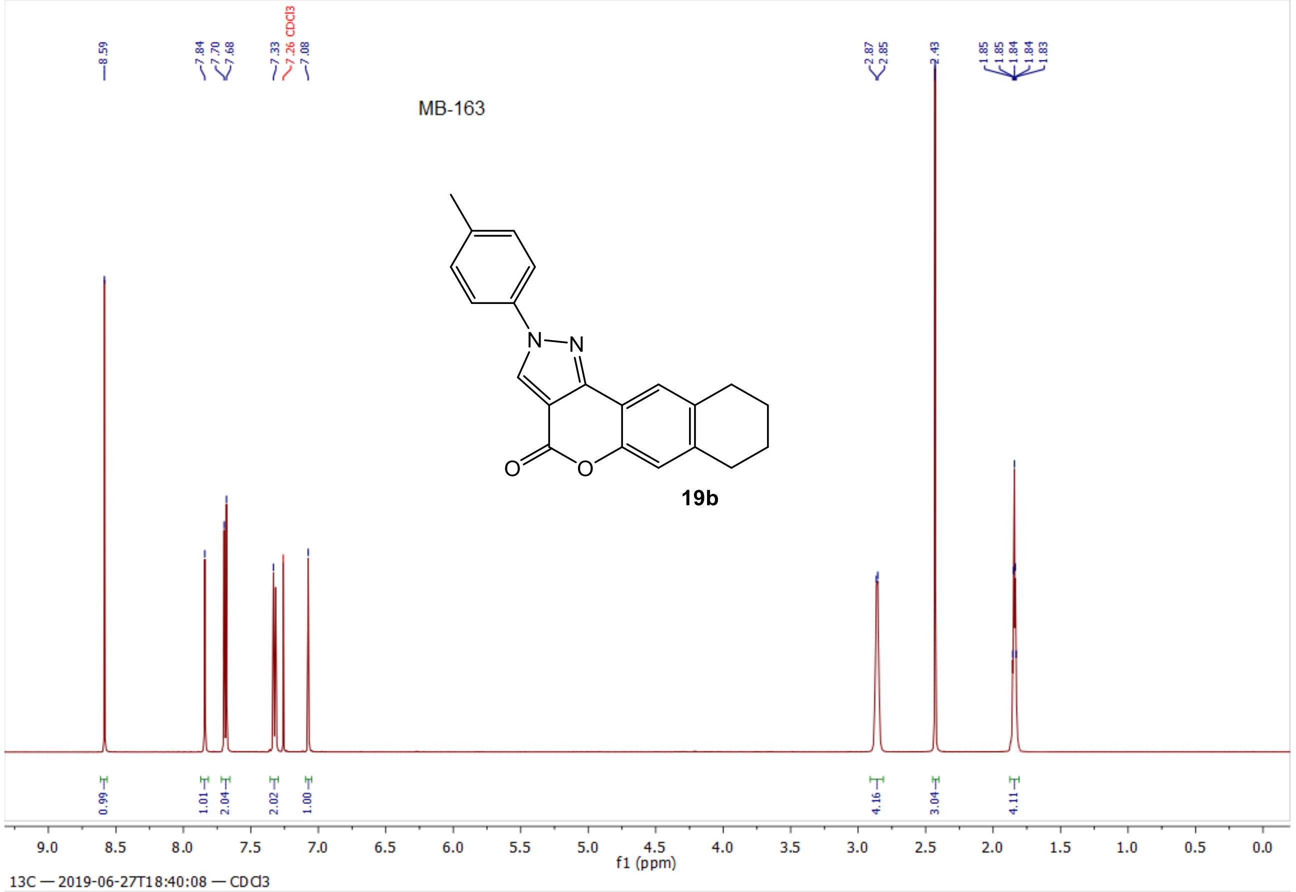

13C — 2019-06-27T18:40:08 — CDCl3

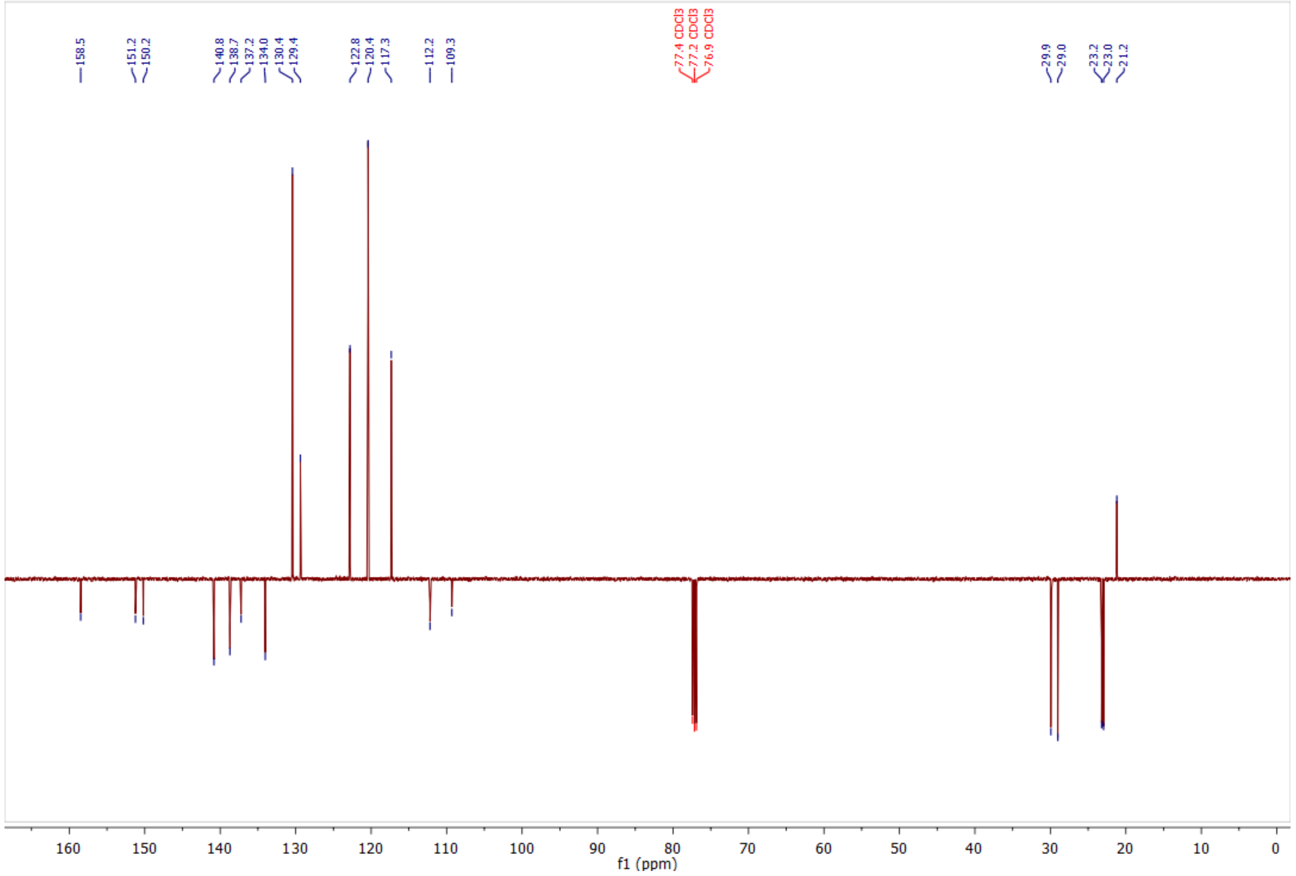

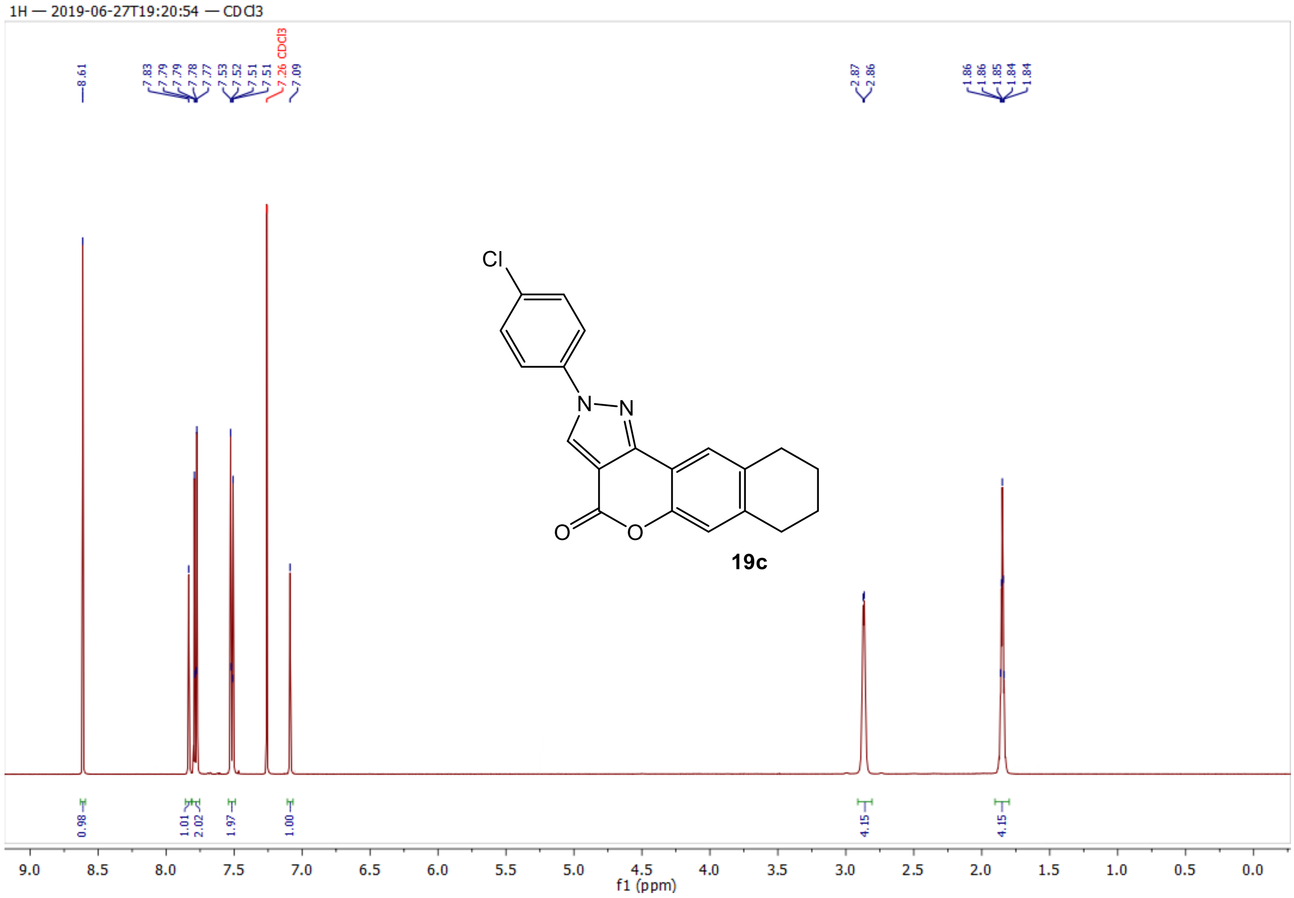
1H — 2019-06-27T19:20:54 — CDCl3
8.61
7.83
7.79
7.79
7.78
7.77
7.53
7.52
7.51
7.51
7.26 CDCl3
7.09
2.87
2.86
1.86
1.86
1.85
1.84
1.84
Cl
N
N
O
O
0.98
1.01
2.02
1.97
1.00
4.15
4.15
f1 (ppm)

19c

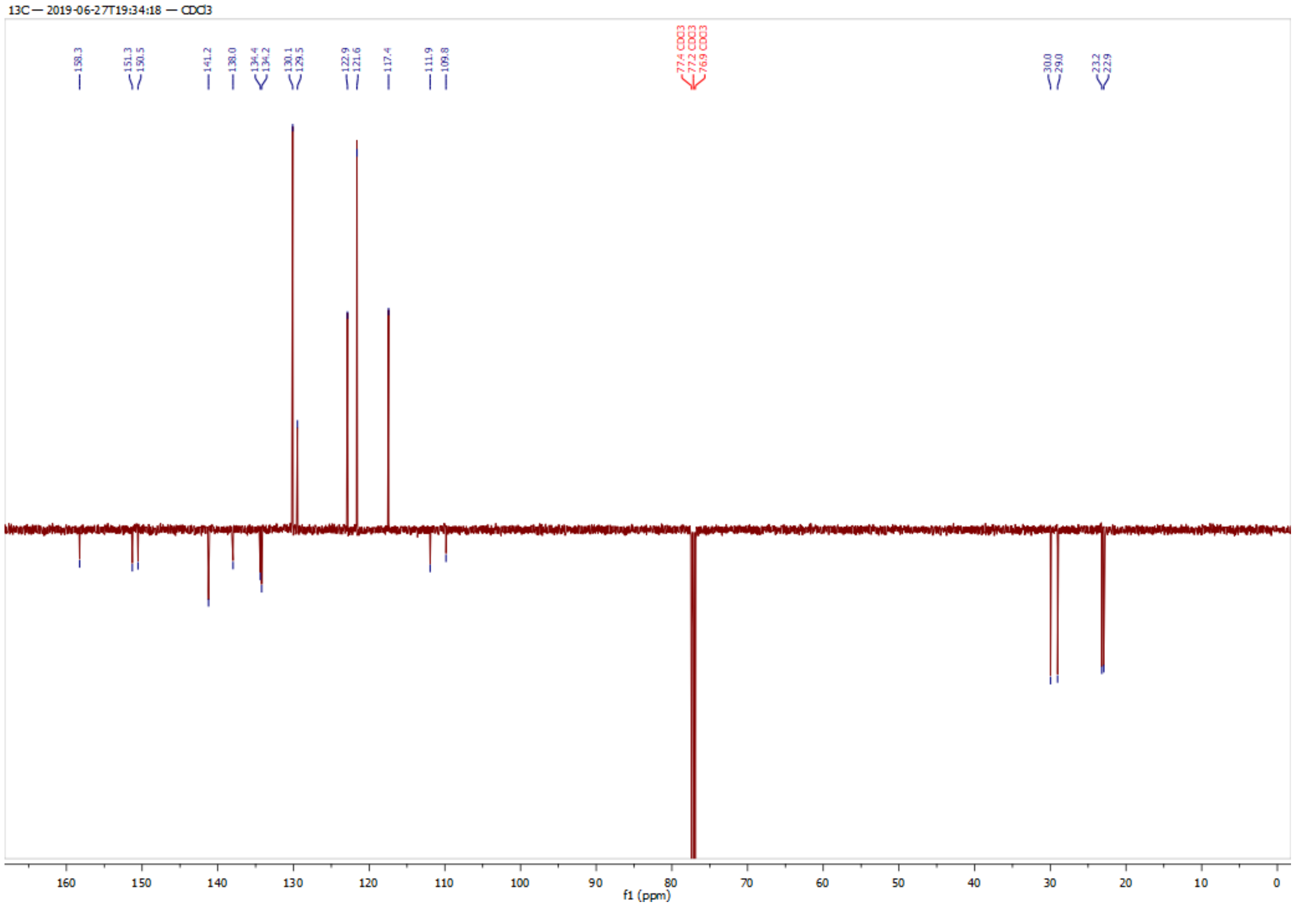
13C — 2019-06-27T19:34:18 — CDCl3
158.3
151.3
150.5
141.2
138.0
134.4
134.2
130.1
129.5
122.9
121.6
117.4
111.9
109.8
77.4 CDCl3
77.2 CDCl3
76.9 CDCl3
30.0
29.0
23.2
22.9
f1 (ppm)

Table S1: Mean±SD values of primary growth inhibitory screen (given as cell viability) used to construct the heat map

	**MRC-5**	**MCF-7**	**HeLa**	**PC-3**	**DU145**
**Control**	99.9±7.2	100±3.6	100.0±3.1	99.9±7.6	100.0±13.9
**4**	≥100±6.6	92.7±2.2	90.0±4.3	88.5±6.5	94.2±8.6
**5**	≥100±7	87.5±	99.6±3.5	79.0±3.8	100.6±6.5
**7a**	≥100±5.7	98.6±5.2	102.7±2.7	87.8±7.1	108.7±9.1
**7b**	100.4±5.1	≥100±4.3	100.1±2.1	98.2±6.5	98.7±15.6
**7c**	78.9±4.3	57.4±10.6	94.1±1.9	36.0±4.5	70.4±3.6
**8a**	99.9±3.3	100.9±4.8	100.4±1.6	54.9±3.0	108.8±4.1
**8b**	≥100±2.4	≥100±8.2	92.2±8.5	67.8±3.8	≥100±10.2
**8c**	≥100±3.3	≥100±5.3	83.1±6.2	94.8±4.0	≥100±5.0
**9a**	≥100±8.1	≥100±2.4	73.8±0.6	82.0±2.2	≥100±5.8
**9b**	≥100±4.2	≥100±0.8	68.0±0.4	86.2±2.4	≥100±4.5
**9c**	89.5±8.7	91.1±3.1	75.7±1.7	94.1±4.2	≥100±0.9
**10a**	93.6±1.7	73.5±7.2	83.2±2.0	63.6±17.8	≥100±15.8
**10b**	96.9±3.8	87.6±0.7	68.3±2.2	88.0±5.6	≥100±6.8
**10c**	96.4±10.1	63.2±2.2	72.4±4.2	81.6±6.8	77.3±6.3
**11a**	≥100±8.2	93.4±3.7	75.4±1.1	59.7±3.2	98.2±5.4
**11b**	≥100±2.2	80.4±2.9	70.1±4.5	74.3±2.3	97.5±10.1
**11c**	≥100±8.4	≥100±0.7	77.9±1.5	87.1±3.8	127.1±4.9
**15**	≥100±3.7	≥100±1.9	53.8±8.3	≥100±2.2	≥100±6.7
**17a**	≥100±7.3	≥100±2.3	53.7±3.3	71.0±20.9	≥100±6.4
**17b**	≥100±14.5	93.2±6.0	≥100±3.9	≥100±3.0	≥100±16.8
**17c**	≥100±4.0	77.5±4.6	≥100±4.0	61.9±2.8	80.3±10.8
**18a**	≥100±0.1	98.0±7.1	≥100±6.5	91.5±12.9	87.0±18.2
**18b**	≥100±2.6	≥100±5.3	≥100±8.7	99.6±7.7	45.3±2.5
**18c**	≥100±2.3	≥100±4.7	≥100±6.6	90.9±3.0	≥100±4.3
**19a**	≥100±3.0	≥100±3.9	≥100±8.7	95.4±1.3	21.6±0.5
**19b**	≥100±0	≥100±4.8	≥100±2.4	84.1±5.2	100.6±6.3
**19c**	≥100±2.5	≥100±3.8	≥100±8.3	79.1±7.8	≥100.6±10.8

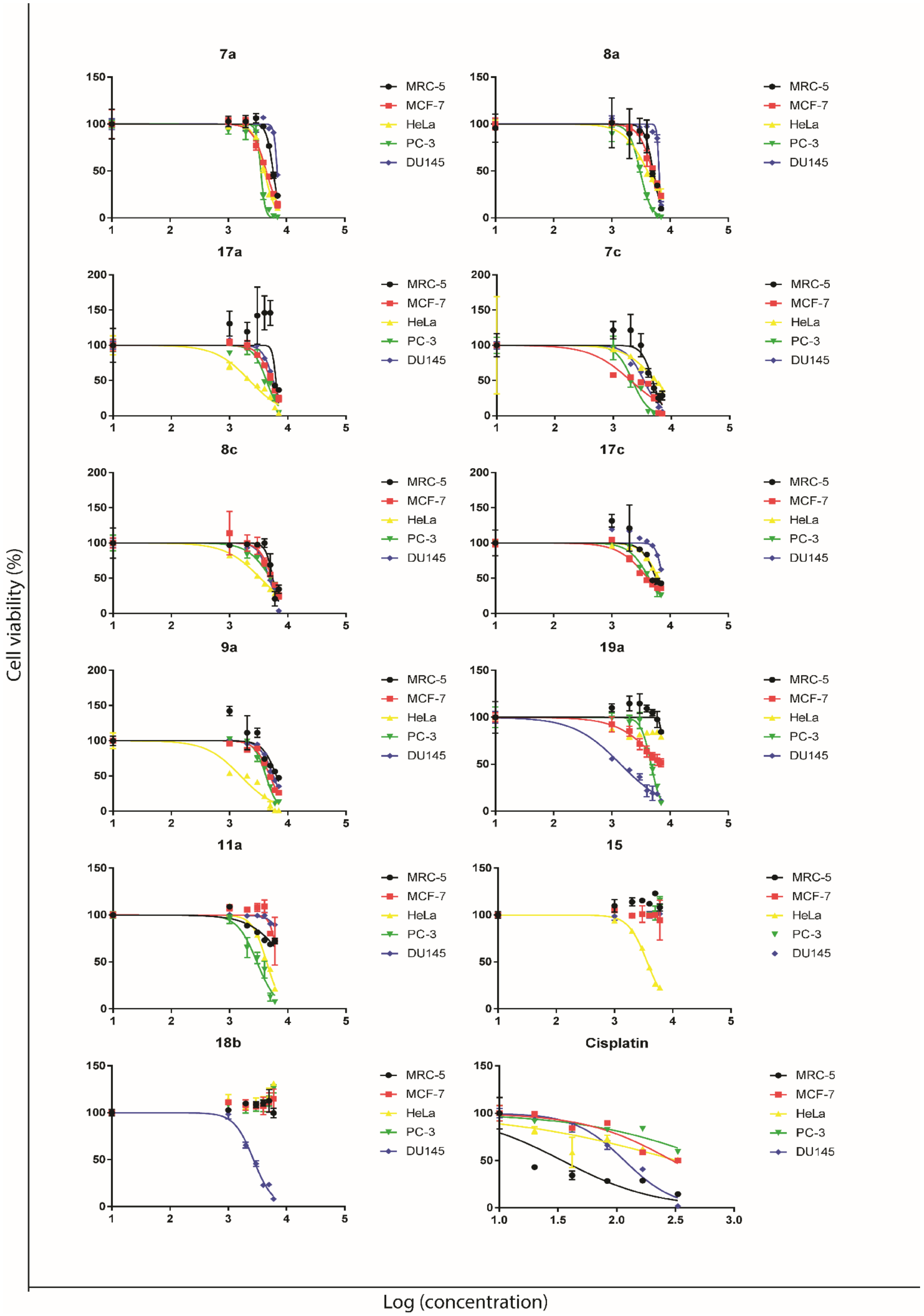

Figure S1: Dose response curves used to evaluate IC_{50} concentrations of selected compounds. X-axis represents nM concentrations of the test compound and μM concentration of cisplatin on logarithmic scale and Y-axis represents cell viability normalized to untreated control.
